# Design, Synthesis and Cytotoxicity of Thiazole-Based Stilbene Analogs as Novel DNA Topoisomerase IB Inhibitors

**DOI:** 10.3390/molecules27031009

**Published:** 2022-02-02

**Authors:** Jin-Chuan Liu, Bo Chen, Jia-Lin Yang, Jian-Quan Weng, Qian Yu, De-Xuan Hu

**Affiliations:** 1 College of Chemical Engineering, Zhejiang University of Technology, Hangzhou 310014, China; jcliu97@163.com (J.-C.L.); chenbo15957143452@163.com (B.C.); youngfoul@163.com (J.-L.Y.); 2 School of Clinical Pharmacy, Guangdong Pharmaceutical University, Guangzhou 510006, China; 3 School of Pharmaceutical Sciences, Sun Yat-sen University, Guangzhou 510006, China; 18923860722@163.com

**Keywords:** thiazole-based stilbene analogs, topoisomerase IB, inhibitor, cytotoxicity

## Abstract

A series of new thiazole-based stilbene analogs were designed, synthesized and evaluated for DNA topoisomerase IB (Top1) inhibitory activity. Top1-mediated relaxation assays showed that the synthesized compounds possessed variable Top1 inhibitory activity. Among them, (*E*)-2-(3-methylstyryl)-4-(4-fluorophenyl)thiazole (**8**) acted as a potent Top1 inhibitor with high Top1 inhibition of ++++ which is comparable to that of CPT. A possible binding mode of compound **8** with Top1–DNA complex was further provided by molecular docking. An MTT assay against human breast cancer (MCF-7) and human colon cancer (HCT116) cell lines revealed that the majority of these compounds showed high cytotoxicity, with IC_50_ values at micromolar concentrations. Compounds **8** and (*E*)-2-(4-tert-butylstyryl)-4-(4-fluorophenyl)thiazole (**11**) exhibited the most potent cytotoxicity with IC_50_ values of 0.78 and 0.62 μM against MCF-7 and HCT116, respectively. Moreover, the preliminary structure–activity relationships of thiazole-based stilbene analogs was also discussed.

## 1. Introduction

Nowadays, cancer is considered to be one of the most common diseases causing death and has been a huge health burden worldwide. According to the latest global data from the World Health Organization (WHO), the cancer burden rose to 19.3 million new cases and 10.0 million cancer deaths in 2020, and new cancer cases are estimated to increase to 30.2 million by 2040 [1]. Despite the invention of several therapeutic approaches, cancer treatment still faces major limitations, and great efforts are being made to develop selective, effective and safer drugs for cancer chemotherapy [2,3].

**Figure 1:**
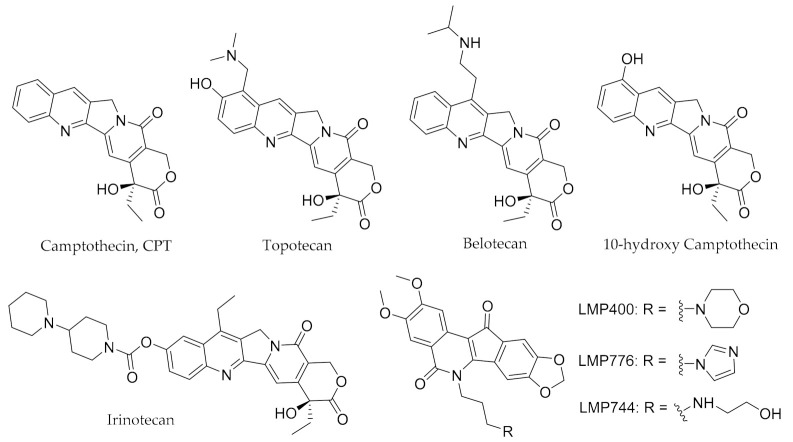
Chemical structures of representative camptothecins derivatives and non-camptothecin Top1 inhibitors.

DNA topoisomerase IB (Top1) is overexpressed among various cancer cell lines and is widely considered an essential nuclear enzyme that regulates DNA topology structure in several cellular metabolic processes including replication and transcription [4,5,6]. Therefore, Top1 can be used as an effective cellular target in anticancer agent discovery [7,8]. To date, the first known Top1 inhibitor camptothecin (CPT) and some structurally modified CPT derivatives, such as topotecan, irinotecan, belotecan and 10-hydroxy camptothecin (Figure 1) have been applied clinically or in clinical trials for cancer treatment [7,9]. Although some CPT-type Top1 inhibitors have been applied clinically, they still suffered from disadvantages such as poor chemical stability, drug resistance, gastrointestinal toxicity or serious side effects [7,10]. Consequently, in the past decades many investigations have focused on the discovery and development of novel non-CPT Top1 inhibitors [11,12,13]. It has been reported that several non-CPT Top1 inhibitors including the indenoisoquinolines LMP400 (indotecan), LMP776 (indimitecan) and LMP744 (Figure 1) have been used in clinical trials [14].

**Figure 2:**
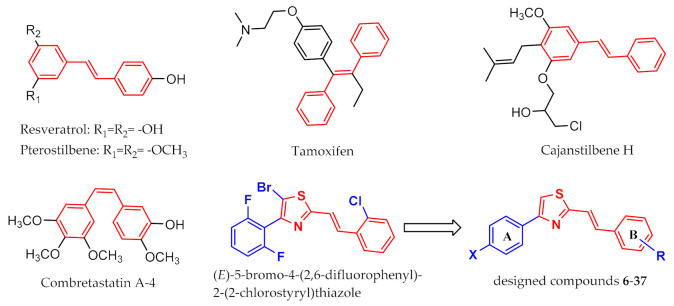
Chemical structures of several natural stilbene derivatives as anticancer agents, the stilbene analogue Top1 inhibitor in laboratory and designed compounds **6**-**37**.

Natural products are an important source of compounds for medicinal chemistry and drug development [15]. Natural stilbenes are phytoalexins produced by some plants in response to pathogen attack and other environmental stresses. Stilbenes have been widely studied for their diverse biological activities and potential therapeutic value [16,17,18]. In particular, extensive research has suggested that many stilbene derivatives such as resveratrol [19], pterostilbene [20], tamoxifen [21], cajanstilbene H [22] and combretastatin A-4 [23] exhibit anticancer activities. Furthermore, thiazoles are also considered to be an important bioactive scaffold due to their various biological effects including anticancer, antioxidant, anti-inflammatory, antimicrobial, antifungal, antimalarial and antiviral activities [24,25,26,27]. For the purpose of discovering novel drug leads, a series of stilbene analogs containing a thiazole moiety were designed and synthesized in our previous work, and the results of Top1-mediated relaxation assays showed that all of them displayed a degree of Top1 inhibitory activity [28]. Among them, (*E*)-5-bromo-4-(2,6-difluorophenyl)-2-(2-chlorostyryl)thiazole (Figure 2) showed a good Top1 inhibition of +++, which suggested that this novel scaffold could potentially lead to a new class of non-CPT Top1 inhibitors. In the present work, it is our purpose to expand the structural diversity of these types of stilbene analogs and to study their potential anticancer activity. Accordingly, a variety of stilbene analogs (**6**–**37**, Figure 2) composed of a 4-(4-halophenyl)thiazole and a substituted phenyl ring were designed and synthesized, and subsequently their Top1 inhibitory activity and cytotoxic activity against two different cancer cell lines were evaluated for anticancer potential. Additionally, the preliminary relationship between structure and activity and the study of their molecular docking are also discussed.

## 2. Results and Discussion

### 2.1. Synthesis

**Scheme 1:**
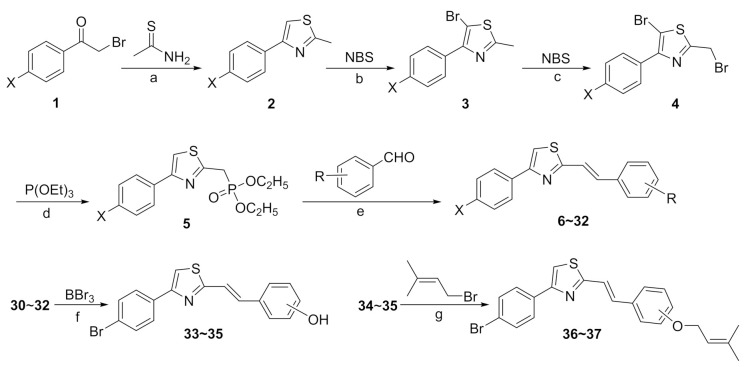
The synthesis of target compounds **6**~**37**. Reagents and conditions: (**a**) DMF, r.t., 5 h; (**b**) ethyl acetate, reflux, 3 h; (**c**) CH_3_CN, r.t., 4 h, 15 W LED; (**d**) reflux, 4 h; (**e**) NaOH, DMF, r.t., 5 h; (**f**) DCM, 0 °C ~ r.t., 6 h, N_2_ atomosphere; (**g**) K_2_CO_3_, anhydrous acetone, reflux, 2 h.

**Scheme 2:**
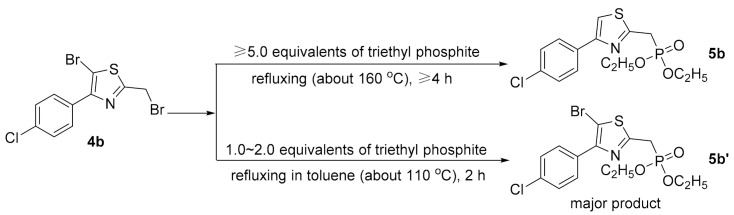
Different phosphonates **5b** and **5b’** depending on different reaction conditions in Arbuzov reaction.

As shown in Scheme 1, the target compounds **6**–**32** were all prepared in five steps including cyclization, bromination, Arbuzov reaction and Wittig–Horner reaction. First, the intermediate 4-(4-halophenyl)-2-methylthiazoles **2** were synthesized by reacting 2-bromo-1-(4-halophenyl)ethan-1-ones **1** with thioacetamide in DMF at room temperature. Subsequently, the 5-position and the benzylic position of the thiazole ring in intermediates **2** were sequentially brominated under different conditions to give 5-bromo-2-(bromomethyl)-4-(4-halophenyl)thiazoles **4**. The 5-position of the thiazole ring was firstly brominated by reacting with NBS via an electrophilic substitution mechanism, and then the obtained intermediates **3** further underwent free-radical benzylic bromination by treatment with NBS under light irradiation to give intermediates 4. According to our experimental results, the sole benzylic brominated product was almost impossible to obtain via a single benzylic bromination step due to the higher reactivity of the proton at the 5-position relative to that at the benzylic position, and the attempt to synthesize intermediates **4** in a one-pot process by using excess NBS only gave a low yield of intermediates **4**. Next, the reaction of intermediates **4** with triethyl phosphite via the Arbuzov reaction procedure gave the phosphonates **5**, which were directly used in the next step without further purification. Finally, the target compounds **6**–**32** were obtained by reacting the above crude phosphonates with various benzaldehydes via the Wittig–Horner reaction. Interestingly, the obtained compounds **6**–**32** were found to have no bromine atom at the 5-position of the thiazole ring. A reasonable explanation is that the bromine at the 5-position was reduced in the Arbuzov reaction procedure due to the known reducibility of triethyl phosphite [29]. Taking intermediate **4b** (X = Cl) as an example to confirm this result, the corresponding phosphonate **5b** bearing no bromine atom at the 5-position of thiazole was isolated and identified. We also found that, in the Arbuzov reaction process, different phosphonate products can be selectively obtained depending on different reaction conditions including the amount of triethyl phosphite, the reaction temperature and the reaction time, as shown in Scheme 2. In addition, considering that some natural hydroxylated or prenylated stilbenes are biologically active, several thiazole-based stilbenes containing hydroxyl or prenyl groups were synthesized to study the structure–activity relationships. Thus, compounds **30**–**32** underwent demethylation by treatment with BBr_3_ in a nitrogen atmosphere to give **33**–**35**, and then compounds **36** and **37** were prepared by reacting **34** and **35**, respectively, with prenyl bromide in the presence of potassium carbonate.

All the target compounds were identified and characterized by ^1^H-NMR, ^13^C-NMR and ESI-HRMS. In the ^1^H-NMR spectra of the target compounds, the signals of the vinylic CH proton connected to the thiazole ring were observed at around 6.93–7.38 ppm, and the peaks of the vinylic CH proton connected to the benzene ring appeared in the 7.10–7.64 ppm range, generally appearing as a doublet with a coupling constant *J* = 16.0 Hz or 16.5 Hz, which indicated that the target compounds are of the *E*-configuration [30]. In the ^13^C NMR spectra, the signals of the vinylic carbon adjacent to the thiazole ring could be found at around 119.3–124.6 ppm, and the peaks of the vinylic carbon adjacent to the benzene ring were observed in the range of 130.8–137.9 ppm. Furthermore, all the target compounds exhibited the M+H^+^ peak in the ESI-HRMS results, which is in agreement with its molecular formula. 

### 2.2. Top1 Inhibitory Activities

The synthesized compounds **6**–**37** were evaluated for Top1 inhibitory activity by measurement of the Top1-mediated DNA relaxation assay. The compounds were tested at 50 µΜ using CPT as positive control, and the results were semi-quantitatively expressed relative to CPT at 50 µΜ as follows: ++++, more than 80% activity; +++, between 40% and 79% activity; ++, between 10% and 39% activity; +, less than 10% activity. The Top1 inhibitory activity is summarized in Table 1. The results showed that all of the synthesized compounds **6**–**37** displayed a degree of Top1 inhibitory activity. Among them, some compounds showed strong Top1 inhibitory activity that was higher than or equipotent with the previously reported (*E*)-5-bromo-4-(2,6-difluorophenyl)-2-(2-chlorostyryl)thiazole, compound **8**, which exhibited an excellent Top1 inhibition of ++++, which is comparable to that of CPT. Compounds **10**, **11**, **13**, **15** and **19** showed a good Top1 inhibition of +++. In phenyl A, the 4-position halogen (X) remarkably affected the inhibition against Top1. Generally, the compounds with fluorine (**6**–**20**) exhibited higher Top1 inhibitory activity than those with chlorine or bromine, probably due to the high electronegativity of the F atom, which reduces the electron cloud density on the benzene ring and increases the π–π stacking interaction between the compound and the base. For instance, although it has the identical 3-Me substituent in phenyl B, compound **8** (X = F) showed a ++++ Top1 inhibitory activity, which was higher than that of **22** (X = Cl, ++) and **26** (X = Br, ++). In phenyl B, the introduction of substituents at the 3-position was beneficial for the Top1 inhibition, and the derivatives presented good tolerance of the 3-substituents, since compounds **8** (++++) and **13** (+++) with electron-donating -Me or -OMe and also compound **19** (+++) with electron-withdrawing -Br had good inhibition against Top1. For substituents at the 4-position, the strong electron-withdrawing -CF_3_ (**10**, +++) and -F (**15**, +++) increased the Top1 inhibitory activity compared to compound **6** (R = H), while the weak electron-withdrawing -Cl (**17**, +) and the electron-donating -Me (**9**, +) and -OMe (14, +) decreased the activity, with the exception of *tert*-Bu (**11**, +++). Compounds **7**, **12**, **16** and **18** exhibited equipotent Top1 inhibition with compound **6**, which indicated that the introduction of substituents at the 2-postion was unable to improve the Top1 inhibitory activity. Unexpectedly, the demethylation products **33**, **34** and **35** showed a weak Top1 inhibition of +, which indicated that the introduction of a hydroxyl group did not increase the Top1 inhibition relative to the compounds with a methoxy group (**30**, **31** and **32**). In addition, the compound with 3-O-prenyl (**36**) exhibited a higher Top1 inhibition of ++ than **31** (3-OMe) and **34** (3-OH), which indicated that the electron-donating O-prenyl at the 3-position was beneficial for the Top1 inhibition, whereas there was no obvious improvement in activity by introducing O-prenyl at the 4-position (**37**, +). To summarize, Top1-mediated assays revealed that the designed and synthesized compounds could be potential Top1 inhibitors, which is valuable in anticancer agent discovery.

**Figure 3:**
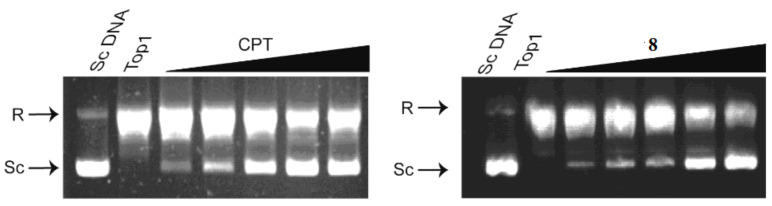
The representive gels for Top1-mediated relaxation assay. Lane 1, pBR322 DNA alone; Lane 2, pBR322 DNA and Top1 without compound; Lanes 3–7, pBR322 DNA, Top1 and compounds at 0.2, 1, 5, 25, 125 μM, respectively. R, relaxed DNA; Sc, supercoiled DNA.

As previously mentioned, compound **8** had the best inhibition activity against Top1, and therefore was selected for further investigation. A semi-quantitative Top1-mediated relaxation assay of compound **8** was performed at concentrations of 0.2, 1, 5, 25 and 125 μM. As shown in Figure 3, compound **8** had strong, dose-dependent inhibitory activity. At high concentration (125 μM), compound **8** showed stronger inhibitory activity than CPT, with 75% of supercoiled DNA remaining; under the same conditions only 58% of supercoiled DNA remained for CPT. At 25 μM, compound 8 displayed equipotent Top1 inhibition with CPT. In contrast, CPT showed higher inhibitory activity at low tested concentrations (0.2, 1 and 5 μM).

**Figure 4:**
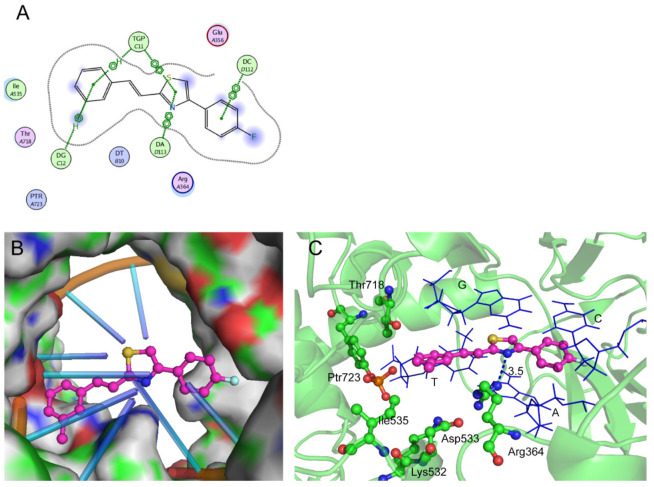
Hypothetical binding mode of **8** in a ternary complex with DNA and Top1 (derived from PDB ID: 1K4T). 2D-ligand interactions (**A**), ball-and-stick model (**B**) and the 3D-ligand interactions (**C**) of **8** and the accepter in the binding pocket. All distances are measured from heavy atom to heavy atom. The carbon atoms of **8** are shown in purple, and the base pairs are displayed as blue lines.

In addition, molecular docking was performed to provide a possible binding mode of compound **8** with the Top1–DNA complex. Compound **8** was docked into the topotecan binding pocket of the Top1–DNA–topotecan ternary complex (PDB ID: 1K4T). The energy-minimized, top-ranked pose of 8 in the ternary complex is displayed in Figure 4. The fluorobenzene scaffold turns into a suitable pocket which binds to the DNA cleavage site (Figure 4A,B) and stacks with the DNA via a strong π–π stack along with the thiazole scaffold and the methylbenzene. What is more, the methylbenzene motif is anchored to the hydrophobic region with Ile535 via van der Waals interactions. Importantly, the N atom of the thiazole forms a hydrogen bond to Arg364 of 3.5 Å (Figure 4C), which is an important contact for the Top1 inhibitory activity. The hydrogen bond between compound **8** and Arg364 and the strong π–π stack with the base play an essential role in the activity. The simulated results generated a reasonable binding mode of compound **8**, further strengthening our concept of this rational design.

### 2.3. Cytotoxicity

The cytotoxicity of the desired compounds was assessed through MTT assay against two human cancer cell lines, human breast cancer (MCF-7) and colon cancer (HCT116), and one normal cell line, human embryonic kidney 293T (HEK293T). CPT and etoposide (VP-16) were used as positive controls. The compounds were incubated with cells for 72 h in a five-dose assay ranging from 0.01 to 100 µΜ. After the drug treatments, MTT solution was added to test the percentage growth of tumor cells. The IC_50_ values, defined as the inhibitor concentration that inhibits growth by 50%, are summarized in Table 2. 

It was found that most of the compounds showed high cytotoxicity, with IC_50_ values at micromolar concentrations. Compounds **8**, **11**, **13**, **15**, **23** and **28** showed strong cytotoxicity against MCF-7 with IC_50_ values of less than 10.0 µΜ, among which compound **8** displayed the highest cytotoxicity (IC_50_ = 0.78 µΜ) with a high Top1 inhibition (++++). Compounds **8**, **11**, **13**, **23**, **26**, **28**, **33** and **35** exhibited high cytotoxicity against HCT116 with IC_50_ values of less than 5.0 µΜ, and compound **11** showed the highest cytotoxicity (IC_50_ = 0.62 µΜ) with a Top1 inhibition of +++. In general, the fluorine-substituted derivatives (**6**–**20**) showed higher cytotoxicity against the two human cancer cell lines than the corresponding chlorinated and brominated derivatives, which indicated that the presence of a fluorine atom at the 4-position in phenyl A is preferred. Compounds **11**, **23** and **28**, with 4-*tert*-Bu in phenyl B, showed high cytotoxicity against MCF-7 (6.31 µΜ for **11**, 7.22 µΜ for **23** and 3.78 µΜ for **28**) and HCT116 (0.62 µΜ for **11**, 2.87 µΜ for **23** and 1.39 µΜ for **28**), although among them the compounds **23** and **28** displayed a weak Top1 inhibition of +, implying that the tert-Bu at the 4-position is beneficial for the cytotoxicity. Compounds with a hydroxy group (**33**, **34** and **35**) showed increased cytotoxicity against the two human cancer cell lines compared to the compounds with a methoxy group (**30**, **31** and **32**), which implied that the introduction of a hydroxy group could improve the cytotoxicity. In addition, the prenylation of **34** and **35** dramatically decreased their cytotoxicity, and the corresponding prenylated products (**36** and **37**) showed low cytotoxicity, with IC_50_ values of more than 100 µΜ. The cytotoxicities of representative compounds 8, 11, 13, 23 and 28 against HEK293T were evaluated, and these compounds were found to possess a degree of cytotoxicity against HEK293T, with IC_50_ values of more than 10.0 µΜ, while CPT showed strong cytotoxicity against HEK293T with an IC_50_ value of 0.10 µΜ.

## 3. Experimental Section

### 3.1. Chemistry

#### 3.1.1. General Information

The major chemical reagents for synthesis were commercial and were used without further purification. 2-Bromo-1-(4-halophenyl)ethan-1-ones **1** were prepared from the corresponding 1-(4-halophenyl)ethan-1-ones according to the procedure reported in the literature [31]. All reactions were carried out under an air atmosphere in dried glassware. The reaction progress was monitored by TLC on silica gel GF254, and spots were visualized by exposure to UV light (254 nm). Melting points were determined using an X-4 apparatus without correction. The purity of the target compounds was recorded on a HPLC-10AT VP Plus (Shimadzu, Tokyo, Japan) equipped with an automatic injector, a 254 nm UV/Vis detector and a C18 analytical column. The analyses were conducted in a two-phase isocratic system with a constant concentration of 70% acetonitrile and 30% H_2_O at 25 °C with a flow rate of 0.7 mL/min. NMR spectra were acquired on a Bruker AVANCE III instrument (500 MHz for ^1^H-NMR and 125 MHz for ^13^C-NMR) using TMS as an internal standard and CDCl_3_ or DMSO-*d_6_* as the solvent. The high-resolution mass spectra (HRMS) were determined on a Shimadzu LCMS-IT-TOF mass spectrometer equipped with an electrospray ionization (ESI) ion source. 

#### 3.1.2. Synthesis of 4-(4-Halophenyl)-2-methylthiazole **2**

Thioacetamide (1.66 g, 22 mmol) was added to a solution of 2-bromo-1-(4-halophenyl)ethanone **1** (20 mmol) in DMF (40 mL), and the mixture was then stirred for 5 h at room temperature. After that, 100 mL H_2_O was added, and the mixture stirred for 30 min; the resulting precipitate was filtered and washed with water to give 4-(4-halophenyl)-2-methylthiazole **2** as a white solid. 4-(4-Fluorophenyl)-2-methylthiazole (**2a**): 3.56 g, yield 92.0%, MP: 183–184 °C (184–185 °C, [32]). ^1^H NMR (500 MHz, CDCl_3_) *δ* 7.86 (dd, J = 8.9, 5.4 Hz, 2H), 7.25 (s, 1H), 7.11 (t, J = 8.7 Hz, 2H), 2.78 (s, 3H). 4-(4-Chlorophenyl)-2-methylthiazole (**2b**): 4.02 g, yield 95.8%, MP: 121–123 °C (122–123 °C, [33]). ^1^H NMR (500 MHz, CDCl_3_) *δ* 7.73 (d, J = 7.5 Hz, 2H), 7.64 (s, 1H), 7.54 (d, J = 7.5 Hz, 2H), 2.85 (s, 3H). 4-(4-Bromophenyl)-2-methylthiazole (**2c**): 4.74 g, yield 93.3%, MP: 132–133 °C (134 °C, [34]). ^1^H NMR (500 MHz, CDCl_3_) *δ* 7.70 (d, J = 7.5 Hz, 2H), 7.66 (s, 1H), 7.55 (d, J = 7.5 Hz, 2H), 2.85 (s, 3H).

#### 3.1.3. Synthesis of 5-Bromo-4-(4-halophenyl)-2-methylthiazole **3**

A mixture of 4-(4-halophenyl)-2-methylthiazole **2** (20 mmol) and NBS (3.92 g, 22 mmol) in ethyl acetate (40 mL) was heated to reflux for 3 h, then the reaction mixture was cooled to room temperature and washed with water. The organic layer was dried over anhydrous Na_2_SO_4_, filtered and then evaporated to give crude **3**, which was then recrystallized from anhydrous ethanol to give pure **3** as a yellow solid. 5-Bromo-4-(4-fluorophenyl)-2-methylthiazole (**3a**): 4.36 g, yield 80.0%. ^1^H NMR (500 MHz, CDCl_3_) δ 7.89 (dd, *J* =10.0, 5.0 Hz, 2H), 7.12 (t, *J* = 8.7 Hz, 2H), 2.70 (s, 3H). ^13^C NMR (125 MHz, CDCl_3_) δ 165.7, 163.6 (*J* = 247.5 Hz), 151.0, 130.5 (*J* = 21.2 Hz), 129.6, 115.4 (dd, *J* = 21.2, 3.7 Hz), 101.9, 19.5. 5-Bromo-4-(4-chlorophenyl)-2-methylthiazole (**3b**): 4.66 g, yield 80.8%. ^1^H NMR (500 MHz, CDCl_3_) δ 7.88–7.84 (m, 2H), 7.42–7.39 (m, 2H), 2.69 (s, 3H). ^13^C NMR (125 MHz, CDCl_3_) δ 165.8, 150.7, 134.3, 131.9, 129.8, 128.5, 102.4, 19.6. 5-Bromo-4-(4-bromophenyl)-2-methylthiazole (**3c**): 5.42 g, yield 81.5%. ^1^H NMR (500 MHz, CDCl_3_) δ 7.82 (d, *J* = 8.5 Hz, 2H), 7.58 (d, *J* = 8.5 Hz, 2H), 2.71 (s, 3H). ^13^C NMR (125 MHz, CDCl_3_) δ 165.9, 150.8, 132.3, 131.5, 130.1, 122.6, 102.5, 19.6. 

#### 3.1.4. Synthesis of 5-Bromo-2-(bromomethyl)-4-(4-halophenyl)thiazole **4**

A mixture of 5-bromo-4-(4-halophenyl)-2-methylthiazole **3** (20 mmol) and NBS (3.92 g, 22 mmol) in acetonitrile (60 mL) was stirred and irradiated by a 15 W LED for 4 h at room temperature. After that, ethyl acetate (100 mL) was added and the mixture was sequentially washed with saturated sodium bicarbonate solution and water. The organic layer was evaporated, and the resultant residue was recrystallized from anhydrous ethanol to give **4** as a yellow solid. 5-Bromo-2-(bromomethyl)-4-(4-fluorophenyl)thiazole (**4a**): 3.80 g, yield 54.0%. ^1^H NMR (500 MHz, CDCl_3_) δ 7.89 (dd, *J* = 10.0, 5.5 Hz, 2H), 7.13 (t, *J* = 8.7 Hz, 2H), 4.69 (s, 2H). ^13^C NMR (125 MHz, CDCl_3_) δ 165.5, 163.8 (*J* = 247.5 Hz), 151.6, 130.5 (*J* = 7.5 Hz), 129.0, 115.4 (*J* = 38.7 Hz), 105.6, 29.7. 5-Bromo-2-(bromomethyl)-4-(4-chlorophenyl)thiazole (**4b**): 4.8 g, yield 65.2%. ^1^H NMR (500 MHz, CDCl_3_) δ 7.86 (d, *J* =8.6 Hz, 2H), 7.41 (d, *J* = 8.6 Hz, 2H), 4.67 (s, 2H). ^13^C NMR (125 MHz, CDCl_3_) δ 165.4, 151.1, 134.5, 131.1, 129.6, 128.4, 105.9, 26.3. 5-Bromo-2-(bromomethyl)-4-(4-bromophenyl)thiazole (**4c**): 4.98 g, yield 60.4%. ^1^H NMR (500 MHz, CDCl_3_) δ 7.81 (d, *J* = 8.6 Hz, 2H), 7.58 (d, *J* = 8.6 Hz, 2H), 4.69 (s, 2H). ^13^C NMR (125 MHz, CDCl_3_) δ 165.7, 151.4, 131.8, 131.6, 130.1, 123.0, 106.2, 26.4. 

#### 3.1.5. Synthesis of Diethyl ((4-(4-Halophenyl)thiazol-2-yl)methyl)phosphonate **5**

A mixture of 5-bromo-2-(bromomethyl)-4-(4-halophenyl)thiazole **4** (10 mmol) and triethyl phosphite (10 mL, 57.9 mmol) was heated under reflux for 4 h. After that, the excess triethyl phosphite was evaporated to give phosphonate **5** as a brown oil, which was used in the next step without any purification. 

#### 3.1.6. General Synthetic Procedure for Compounds **6**–**32**

The above phosphonate **5**, substituted benzaldehyde (12 mmol) and NaOH (0.48 g, 12 mmol) were dissolved in 20 mL of anhydrous DMF. The resultant mixture was stirred at room temperature for 5 h. 50 mL of H_2_O was added and stirred for an additional 0.5 h, then extracted with chloroform (3 × 30 mL). After that, the organic layer was dried using anhydrous Na_2_SO_4._, then evaporated, and the residue was purified by silica gel column chromatography (petroleum ether/ethyl acetate: 5/1) to give the target products **6**–**32** (Figures S1–S32).

*(E)-4-(4-fluorophenyl)-2-styrylthiazole (**6**)*. Yellow solid, yield 51.2%, MP: 129–130 °C, purity: 98.6%. ^1^H NMR (500 MHz, CDCl_3_) *δ* 7.93 (dd, *J* = 9.0, 5.5 Hz, 2H), 7.58 (d, *J* = 7.0 Hz, 2H), 7.49 (d, *J* = 16.0 Hz, 1H), 7.42 (t, *J* = 7.0 Hz, 2H), 7.39–7.32 (m, 3H), 7.14 (t, *J* = 9.0 Hz, 2H); ^13^C NMR (125 MHz, CDCl_3_) *δ* 167.0, 163.8 (d, *J* = 250.0 Hz), 155.2, 135.8, 134.7, 130.7 (d, *J* = 3.75 Hz), 129.0, 128.9, 128.2 (d, *J* = 12.5 Hz), 127.2, 121.5, 115.8 (d, *J* = 25.0 Hz), 111.8. HRMS (ESI) calculated C_17_H_13_FNS [M+H]^+^ 282.0674, found 282.0764.

*(E)-2-(2-methylstyryl)-4-(4-fluorophenyl)thiazole (**7**).* Yellow solid, yield 62.4%, MP: 74–75 °C, purity: 99.1%. ^1^H NMR (500 MHz, CDCl_3_) *δ* 7.94 (dd, *J* = 8.5, 5.5 Hz, 2H), 7.76 (d, *J* = 16.0 Hz, 1H), 7.69–7.62 (m, 1H), 7.33 (s, 1H), 7.31–7.21 (m, 4H), 7.16 (t, *J* = 9.0 Hz, 2H), 2.51 (s, 3H); ^13^C NMR (125 MHz, CDCl_3_) *δ* 167.2, 162.8 (d, *J* = 250.0 Hz), 155.2, 136.6, 134.7, 132.3, 130.7, 128.9, 128.2 (d, *J* = 8.8 Hz), 126.4, 125.7, 122.5, 115.7 (d, *J* = 25.0 Hz), 111.7, 19.9. HRMS (ESI) calculated C_18_H_15_BrFNS [M+H]^+^ 296.0834, found 296.0888. 

*(E)-2-(3-methylstyryl)-4-(4-fluorophenyl)thiazole(**8**).* Yellow solid, yield 49.5%, MP: 148–149 °C, purity: 98.9%. ^1^H NMR (500 MHz, CDCl_3_) *δ* 7.93 (dd, *J* = 8.5, 5.5 Hz, 2H), 7.46 (d, *J* = 16.0 Hz, 1H), 7.39 (d, *J* = 8.5 Hz, 2H), 7.35 (d, *J* = 16.0 Hz, 1H), 7.33 (s, 1H), 7.31 (t, *J* = 7.5 Hz, 1H), 7.21–7.09 (m, 3H), 2.41 (s, 3H); ^13^C NMR (125 MHz, CDCl_3_) *δ* 167.0, 162.8 (d, *J* = 237.5 Hz), 155.1, 138.5, 135.6, 134.8, 130.7 (d, *J* = 3.8 Hz), 129.8, 128.8, 128.2 (d, *J* = 12.5 Hz), 127.8, 124.3, 121.3, 115.6 (d, *J* = 25.0 Hz), 111.6, 21.4. HRMS (ESI) calculated C_18_H_15_FNS [M+H]^+^ 296.0831, found 296.0883. 

*(E)-2-(4-methylstyryl)-4-(4-fluorophenyl)thiazole (**9**).* Yellow solid, yield 62.3%, MP: 164–166 °C, purity: 98.5%.; ^1^H NMR (500 MHz, CDCl_3_) *δ* 7.92 (t, *J* = 6.0 Hz, 2H), 7.53–7.39 (m, 3H), 7.32 (t, *J* = 6.0 Hz, 2H), 7.22 (d, *J* = 7.5 Hz, 2H), 7.14 (t, *J* = 8.5 Hz, 2H), 2.40 (s, 3H); ^13^C NMR (125 MHz, CDCl_3_) *δ* 167.2, 162.8 (d, *J* = 237.5 Hz), 155.1, 139.2, 134.7, 133.0, 130.7 (d, *J* = 3.8 Hz), 129.6, 128.2 (d, *J* = 12.5 Hz), 127.1, 120.6, 115.6 (d, *J* = 25.0 Hz), 111.5, 21.4. HRMS (ESI) calculated C_18_H_15_FNS [M+H]^+^ 296.0831, found 296.0890. 

*(E)-2-(4-(trifluoromethyl)styryl)-4-(4-fluorophenyl)thiazole (**10**).* Yellow solid, yield 66.4%, MP: 111–113 °C, purity: 97.4%. ^1^H NMR (500 MHz, CDCl_3_) *δ* 7.93 (dd, *J* = 9.0, 5.5 Hz, 2H), 7.66 (s, 4H), 7.51 (d, *J* = 16.5 Hz, 1H), 7.41 (d, *J* = 16.5 Hz, 2H), 7.15 (t, *J* = 8.5 Hz, 2H); ^13^C NMR (125 MHz, CDCl_3_) *δ* 165.9, 162.9 (d, *J* =250.0 Hz), 155.5, 139.2, 132.7, 130.5 (d, *J* = 3.8 Hz), 128.2 (d, *J* = 8.8 Hz), 127.2, 125.9 (q, *J* = 3.8 Hz), 123.7, 115.8 (d, *J* = 12.5 Hz), 112.4. HRMS (ESI) calculated C_18_H_12_F_4_NS [M+H]^+^ 350.0548, found 350.0599.

*(E)-2-(4-tert-butylstyryl)-4-(4-fluorophenyl)thiazole(**11**).* Yellow solid, yield 61.6%, MP: 146–148 °C, purity: 96.8%. ^1^H NMR (500 MHz, CDCl_3_) *δ* 7.93 (dd, *J* = 8.5, 5.5 Hz, 2H), 7.53 (d, *J* = 8.5 Hz, 2H), 7.44 (m, 3H), 7.34 (d, *J* = 15.0 Hz, 2H), 7.14 (t, *J* = 8.5 Hz, 2H), 1.37 (s, 9H); ^13^C NMR (125 MHz, CDCl_3_) *δ* 167.2, 162.8 (d, *J* = 250.0 Hz), 155.1, 152.4, 134.6, 132.9, 130.7 (d, *J* = 3.8 Hz), 128.2 (d, *J* = 12.5 Hz), 126.9, 125.9, 120.7, 115.7 (d, *J* = 12.5 Hz), 111.5, 34.8, 31.2. HRMS (ESI) calculated C_21_H_21_FNS [M+H]^+^ 338.1300, found 338.1381. 

*(E)-2-(2-methoxystyryl)-4-(4-fluorophenyl)thiazole (**12**).* Yellow solid, yield 55.9%, MP: 73–74 °C, purity: 99.3%. ^1^H NMR (500 MHz, CDCl_3_) *δ* 7.93 (dd, *J* = 9.0, 5.5 Hz, 2H), 7.80 (d, *J* = 16.0 Hz, 1H), 7.61 (dd, *J* = 8.0, 1.5 Hz, 1H), 7.47 (d, *J* = 16.0 Hz, 1H), 7.36–7.29 (m, 2H), 7.14 (t, *J* = 8.5 Hz, 2H), 7.01 (t, *J* = 7.5 Hz, 1H), 6.94 (d, *J* = 8.5 Hz, 1H), 3.93 (s, 3H); ^13^C NMR (125 MHz, CDCl_3_) *δ* 167.8, 162.7 (d, *J* = 237.5 Hz), 157.5, 154.9, 130.8 (d, *J* = 12.5 Hz), 130.0, 128.1 (d, *J* = 7.5 Hz), 127.6, 124.6, 122.2, 120.8, 115.5 (d, *J* = 12.5 Hz), 111.4, 111.0, 55.4. HRMS (ESI) calculated C_18_H_15_FNOS [M+H]^+^ 312.0780, found 312.0830. 

*(E)-2-(3-methoxystyryl)-4-(4-fluorophenyl)thiazole (**13**).* Yellow solid, yield 57.6%, MP: 115–116 °C, purity: 98.2%. ^1^H NMR (500 MHz, CDCl_3_) *δ* 7.92 (dd, *J* = 9.0, 5.5 Hz, 2H), 7.44 (d, *J* = 16.0 Hz, 1H), 7.37–7.28 (m, 3H), 7.19–7.08 (m, 4H), 6.91 (dd, *J* = 8.0, 2.0 Hz, 1H), 3.85 (s, 3H); ^13^C NMR (125 MHz, CDCl_3_) *δ* 166.6, 162.7 (d, *J* = 235.7 Hz), 159.9, 155.0, 137.0, 134.4, 130.6 (d, *J* = 12.5 Hz), 129.7, 128.1 (d, *J* = 12.5 Hz), 121.6, 119.8, 115.5 (d, *J* = 25.0 Hz), 114.8, 112.0, 111.7, 55.1. HRMS (ESI) calculated C_18_H_15_FNOS [M+H]^+^ 312.0780, found 312.0836. 

*(E)-2-(4-methoxystyryl)-4-(4-fluorophenyl)thiazole (**14**).* Yellow solid, yield 64.9%, MP: 124–125 °C, purity: 97.7%. ^1^H NMR (500 MHz, CDCl_3_) *δ* 7.92 (dd, *J* = 8.5, 5.5 Hz, 2H), 7.52 (d, *J* = 9.0 Hz, 2H), 7.43 (d, *J* = 16.0 Hz, 1H), 7.31 (s, 1H), 7.23 (d, *J* = 16.0 Hz, 1H), 7.14 (t, *J* = 9.0 Hz, 2H), 6.94 (d, *J* = 8.5 Hz, 2H), 3.86 (s, 3H); ^13^C NMR (125 MHz, CDCl_3_) *δ* 167.4, 162.7 (d, *J* = 250.0 Hz), 160.4, 155.0, 134.3, 130.8 (d, *J* = 2.5 Hz), 128.6, 128.5, 128.2 (d, *J* =12.5 Hz), 119.4, 115.6 (d, *J* = 25.0Hz), 114.4, 111.2, 55.4. HRMS (ESI) calculated C_18_H_15_FNOS [M+H]^+^ 312.0880, found 312.0835.

*(E)-2-(4-fluorostyryl)-4-(4-fluorophenyl)thiazole (**15**).* Yellow solid, yield 54.0%, MP: 147–149 °C, purity: 97.8%. ^1^H NMR (500 MHz, CDCl_3_) *δ* 7.92 (dd, *J* = 8.5, 5.5 Hz, 2H), 7.54 (dd, *J* = 8.5, 5.5 Hz, 2H), 7.44 (d, *J* = 16.5 Hz, 1H), 7.34 (s, 1H), 7.26 (d, *J* = 16.5 Hz, 1H), 7.12 (dt, *J* = 21.5, 8.5 Hz, 4H); ^13^C NMR (125 MHz, CDCl_3_) *δ* 166.6, 163.0 (dd, *J* = 212.5, 37.5 Hz), 155.2, 133.1, 131.9 (d, *J* = 3.5 Hz), 130.6 (d, *J* = 3.5 Hz), 128.78 (d, *J* =12.5 Hz), 128.2 (d, *J* = 12.5 Hz), 121.21, 116.0 (d, *J* = 12.5 Hz), 115.7 (d, *J* = 25.0 Hz), 111.7. HRMS (ESI) calculated C_17_H_12_F_2_NS [M+H]^+^ 300.0580, found 300.0642. 

*(E)-2-(2-chlorostyryl)-4-(4-fluorophenyl)thiazole(****16****).* Yellow solid, yield 63.5%, MP: 97–98 °C, purity: 98.4%. ^1^H NMR (500 MHz, CDCl_3_) *δ* 7.93 (t, 2H), 7.87 (d, *J* = 16.5 Hz, 1H), 7.73 (d, *J* = 7.5 Hz, 1H), 7.44 (d, *J* = 7.5 Hz, 1H), 7.41–7.26 (m, 4H), 7.15 (t, *J* = 8.0 Hz, 2H); ^13^C NMR (125 MHz, CDCl_3_) *δ* 166.6, 162.9 (d, *J* = 237.5 Hz), 155.4, 134.0, 133.9, 130.6 (d, *J* = 12.5 Hz), 130.5, 130.1, 129.8, 128.2 (d, *J* = 7.5 Hz), 127.1, 126.8, 125.8, 124.0, 115.7 (d, *J* = 25.0 Hz), 112.2. HRMS (ESI) calculated C_17_H_12_ClFNS [M+H]^+^ 316.0285, found 316.0357.

*(E)-2-(4-chlorostyryl)-4-(4-fluorophenyl)thiazole (**17**).* Yellow solid, yield 53.3%, MP: 171–173 °C, purity: 98.0%. ^1^H NMR (500 MHz, CDCl_3_) *δ* 7.92 (dd, *J* = 9.0, 5.5 Hz, 2H), 7.50 (d, *J* = 8.5 Hz, 2H), 7.44 (d, *J* = 16.5 Hz, 1H), 7.40–7.35 (m, 3H), 7.32 (d, *J* = 16.5 Hz, 1H), 7.14 (t, *J* = 9.0 Hz, 2H); ^13^C NMR (125 MHz, CDCl_3_) *δ* 166.5, 162.9 (d, *J* = 250.0 Hz), 155.4, 134.7, 134.3, 133.2, 130.7 (d, *J* = 12.5 Hz), 129.2, 128.3, 128.3 (d, *J* =12.5 Hz), 122.0, 115.8 (d, *J* = 12.5 Hz), 112.0. HRMS (ESI) calculated C_17_H_11_ClFNS [M+H]^+^ 316.0285, found 316.0359. 

*(E)-2-(2-bromostyryl)-4-(4-fluorophenyl)thiazole (**18**).* Yellow solid, yield 49.9%, MP: 94–95 °C, purity: 97.1%. ^1^H NMR (500 MHz, CDCl_3_) *δ* 7.92 (dd, *J* = 8.5, 5.5 Hz, 2H), 7.82 (d, *J* = 16.5 Hz, 1H), 7.70 (dd, *J* = 7.5, 1.5 Hz, 1H), 7.63 (dd, *J* = 8.0, 1.0 Hz, 1H), 7.41–7.27 (m, 3H), 7.14 (m, 4H); ^13^C NMR (125 MHz, CDCl_3_) *δ* 166.5, 162.8 (d, *J* = 250 Hz), 155.3, 135.6, 133.3, 133.2, 130.9, 130.6 (d, *J* = 2.5 Hz), 130.0, 128.2 (d, *J* = 12.5 Hz), 127.7, 127.0, 124.5, 124.1, 115.7 (d, *J* = 25 Hz), 112.2. HRMS (ESI) calculated C_17_H_12_BrFNS [M+H]^+^ 359.9780, found 359.9854. 

*(E)-2-(3-bromostyryl)-4-(4-fluorophenyl)thiazole (**19**).* Yellow solid, yield 50.8%, MP: 142–144 °C, purity: 97.5%. ^1^H NMR (500 MHz, CDCl_3_) *δ* 7.92 (dd, *J* = 8.5, 5.5 Hz, 2H), 7.72 (s, 1H), 7.47 (t, *J* = 7.5 Hz, 2H), 7.41 (d, *J* = 16.5 Hz, 1H), 7.37 (s, 1H), 7.33 (d, *J* = 16.5 Hz, 1H), 7.27 (t, *J* = 8.0 Hz, 1H), 7.14 (t, *J* = 8.5 Hz, 2H); ^13^C NMR (125 MHz, CDCl_3_) δ 166.1, 162.8 (d, *J* = 250.0 Hz), 155.4, 137.9, 132.8, 131.7, 130.6 (d, *J* = 12.5 Hz), 130.4, 129.9, 128.2 (d, *J* = 12.5 Hz), 125.7, 123.1, 122.7, 115.8, 115.7 (d, *J* = 25.0 Hz), 112.2. HRMS (ESI) calculated C_17_H_12_BrFNS [M+H]^+^ 359.9780, found 359.9857. 

*(E)-2-(2,4-Dichlorostyryl)-4-(4-fluorophenyl)thiazole (**20**).* Yellow solid, yield 49.3%, MP: 131–133 °C, purity: 97.0%. ^1^H NMR (500 MHz, CDCl_3_) *δ* 7.92 (dd, *J* = 8.5, 5.5 Hz, 2H), 7.78 (d, *J* = 16.0 Hz, 1H), 7.64 (d, *J* = 8.5 Hz, 1H), 7.45 (d, *J* = 1.5 Hz, 1H), 7.39 (s, 1H), 7.33 (d, *J* = 16.0 Hz, 1H), 7.30–7.26 (m, 1H), 7.14 (t, *J* = 9.0 Hz, 2H); ^13^C NMR (125 MHz, CDCl_3_) *δ* 166.1, 162.9 (d, *J* = 250.0 Hz), 155.5, 134.9, 134.5, 132.5, 130.5 (d, *J* = 2.5 Hz), 129.9, 129.2, 128.2 (d, *J* = 8.8 Hz), 127.6, 127.5, 124.3, 115.7 (d, *J* = 25.0 Hz), 112.4. HRMS (ESI) calculated C_17_H_11_Cl_2_FNS [M+H]^+^ 349.9895, found 349.9967. 

*(E)-4-(4-chlorophenyl)-2-(2-methylstyryl)thiazole (**21**).* Yellow solid, yield 59.8%, MP: 83–84 °C, purity: 97.7%. ^1^H NMR (500 MHz, CDCl_3_) *δ* 7.90 (d, *J* = 8.5 Hz, 2H), 7.75 (d, *J* = 16.5 Hz, 1H), 7.67–7.63 (m, 1H), 7.45–7.39 (m, 3H), 7.27–7.21 (m, 3H), 7.28 ((d, *J* = 16.0 Hz, 1H), 2.50 (s, 3H); ^13^C NMR (125 MHz, CDCl_3_) δ 167.3, 155.0, 136.6, 134.6, 134.0, 132.9, 132.5, 130.7, 128.9, 127.6, 126.4, 125.7, 122.5, 112.3, 19.9. HRMS (ESI) calculated C_18_H_15_ClNS [M+H]^+^ 3312.0535, found 312.0615.

*(E)-4-(4-chlorophenyl)-2-(3-methylstyryl)thiazole (**22**).* Yellow solid, yield 55.6%, MP: 133–134 °C, purity: 99.0%. ^1^H NMR (500 MHz, CDCl_3_) *δ* 7.91–7.86 (m, 1H), 7.85–7.79 (m, 1H), 7.46 (d, *J* = 16.5 Hz, 1H), 7.44–7.41 (m, 1H), 7.39 (d, *J* = 7.0 Hz, 3H), 7.34–7.29 (m, 2H), 7.19 (dd, *J* = 14.5, 7.0 Hz, 1H), 7.01–6.88 (m, 1H), 2.40 (d, *J* = 8.0 Hz, 3H); 13C NMR (125 MHz, CDCl_3_) *δ* 167.1, 154.9, 138.5, 135.6, 135.0, 134.0, 132.9, 129.9, 128.9, 128.8, 127.9, 127.7, 127.6, 124.3, 121.2, 112.3, 21.4. HRMS (ESI) calculated C_18_H_15_ClNS [M+H]^+^ 312.0535, found 312.0618.

*(E)-4-(4-chlorophenyl)-2-(4-(tert-butyl)styryl)thiazole (**23**).* Yellow solid, yield 51.2%, MP: 74–75 °C, purity: 98.2%. ^1^H NMR (500 MHz, CDCl_3_) *δ* 7.91–7.87 (m, 2H), 7.52 (d, *J* = 8.0 Hz, 2H), 7.47 (d, *J* = 16.5 Hz, 1H), 7.45–7.41 (m, 4H), 7.39 (s, 1H), 7.33 (d, *J* = 16.0 Hz, 1H), 1.36 (s, 9H); ^13^C NMR (125 MHz, CDCl_3_) *δ* 167.3, 154.9, 152.4, 134.7, 134.0, 132.9, 128.9, 127.7, 127.0, 125.9, 120.7, 112.2, 100.0, 34.8, 31.2. HRMS (ESI) calculated C_21_H_21_ClNS [M+H]^+^ 354.1005, found 354.1086.

*(E)-4-(4-chlorophenyl)-2-(2-methoxystyryl)thiazole (**24**).* Yellow solid, yield 67.1%, MP: 93–95 °C, purity: 99.4%. ^1^H NMR (500 MHz, CDCl_3_) *δ* 7.91–7.87 (m, 2H), 7.79 (d, *J* = 16.5 Hz, 1H), 7.60 (dd, *J* = 7.5, 1.5 Hz, 1H), 7.46 (d, *J* = 16.0 Hz, 1H), 7.44–7.40 (m, 2H), 7.37 (s, 1H), 7.35–7.31 (m, 1H), 7.01 (t, *J* = 7.5 Hz, 1H), 6.95 (d, *J* = 8.0 Hz, 1H), 3.94 (s, 3H); ^13^C NMR (125 MHz, CDCl_3_) δ 168.0, 157.6, 154.8, 133.9, 133.0, 130.2, 128.9, 127.7, 124.6, 122.2, 120.8, 112.2, 111.1, 55.5. HRMS (ESI) calculated C_18_H_15_ClNOS [M+H]^+^ 328.0485, found 328.0571.

*(E)-4-(4-chlorophenyl)-2-(2-chlorostyryl)thiazole (**25**).* Yellow solid, yield 63.2%, MP: 109–110 °C, purity: 98.1%. ^1^H NMR (500 MHz, CDCl_3_) *δ* 7.91–7.88 (m, 2H), 7.80–7.77 (m, 1H), 7.73 (dd, *J* = 7.5, 2.0 Hz, 1H), 7.52–7.49 (m, 1H), 7.44 (d, *J* = 4.5 Hz, 2H), 7.38 (d, *J* = 2.5 Hz, 1H), 7.36 (d, *J* = 10.5 Hz, 1H), 7.30 (d, *J* = 8.0 Hz, 2H); ^13^C NMR (125 MHz, CDCl_3_) δ 166.6, 155.2, 134.1, 134.0, 133.8, 132.8, 132.0, 130.6, 130.1, 129.8, 128.9, 127.6, 127.1, 126.8, 123.9, 112.9. HRMS (ESI) calculated C_17_H_12_Cl_2_NS [M+H]^+^ 331.9989, found 332.0073.

*(E)-4-(4-bromophenyl)-2-(3-methylstyryl)thiazole (**26**).* Yellow solid, yield 63.4%, MP: 174–176 °C, purity: 98.7%. ^1^H NMR (500 MHz, CDCl_3_) *δ* 7.83 (d, *J* = 8.5 Hz, 2H), 7.58 (d, *J* = 8.0 Hz, 2H), 7.46 (d, *J* = 16.0 Hz, 1H), 7.41 (d, *J* = 16.0 Hz, 1H), 7.40–7.34 (m, 3H), 7.30 (t, *J* = 7.5 Hz, 1H), 7.18 (d, *J* = 7.5 Hz, 1H), 2.41 (s, 3H); ^13^C NMR (125 MHz, CDCl_3_) *δ* 167.2, 155.0, 138.5, 135.6, 135.1, 133.3, 131.9, 129.9, 128.8, 128.0, 124.4, 122.2, 121.3, 112.4, 21.4. HRMS (ESI) calculated C_18_H_15_BrNS [M+H]^+^ 356.0030, found 356.0105.

*(E)-4-(4-bromophenyl)-2-(4-(trifluoromethyl)styryl)thiazole(****27****).* Yellow solid, yield 54.8%, MP: 102–103 °C, purity: 97.3%. ^1^H NMR (500 MHz, CDCl_3_) *δ* 7.84 (d, *J* = 8.5 Hz, 1H), 7.72 (d, *J* = 8.5 Hz, 1H), 7.67 (s, 4H), 7.59 (d, *J* = 8.5 Hz, 1H), 7.55 (d, *J* = 8.5 Hz, 1H), 7.51–7.43 (m, 1H), 7.39 (d, *J* = 18.0 Hz, 1H), 6.96 (d, *J* = 2.0 Hz, 1H); ^13^C NMR (125 MHz, CDCl_3_) *δ* 163.4, 155.4, 135.0, 133.0, 132.5, 132.3, 130.8 (q, *J* = 32.5 Hz), 128.3 (d, *J* = 3.3 Hz), 127.5, 125.7 (q, *J* = 3.8 Hz), 125.0 (d, *J* = 268.0 Hz), 123.0, 120.7, 119.8, 119.2. HRMS (ESI) calculated C_18_H_12_BrF_3_NS [M+H]^+^ 409.0748, found 409.9821.

*(E)-4-(4-bromophenyl)-2-(4-(tert-butyl)styryl)thiazole (**28**).* Yellow solid, yield 59.3%, MP: 89–90 °C, purity: 97.1%. ^1^H NMR (500 MHz, CDCl_3_) *δ* 7.83 (d, *J* = 8.0 Hz, 2H), 7.58 (d, *J* = 8.5 Hz, 2H), 7.52 (d, *J* = 8.5 Hz, 2H), 7.47 (d, *J* = 17.0 Hz, 2H), 7.42 (d, *J* = 16.5 Hz, 2H), 7.34 (d, *J* = 16.0 Hz, 1H), 1.37 (d, *J* = 6.5 Hz, 9H); ^13^C NMR (125 MHz, CDCl_3_) *δ* 167.5, 154.8, 152.5, 135.6, 135.0, 133.2, 132.9, 131.9, 128.7, 128.0, 127.0, 125.7, 122.3, 120.6, 112.3, 34.8, 31.3. HRMS (ESI) calculated C_21_H_21_BrNS [M+H]^+^ 398.0500, found 398.0576.

*(E)-4-(4-bromophenyl)-2-(4-fluorostyryl)thiazole (**29**).* Yellow solid, yield 45.4%, MP: 176–178 °C, purity: 98.4%. ^1^H NMR (500 MHz, CDCl_3_) *δ* 7.83 (d, *J* = 8.0 Hz, 2H), 7.58 (d, *J* = 8.5 Hz, 2H), 7.57–7.53 (m, 2H), 7.46 (d, *J* = 16.0 Hz, 1H), 7.42 (s, 1H), 7.11 (t, *J* = 8.5 Hz, 3H); ^13^C NMR (125 MHz, CDCl_3_) δ 159.5, 155.1, 147.5, 146.8, 145.8, 133.6, 131.9, 131.0, 128.8 (d, *J* = 8.0 Hz), 127.9, 122.2, 121.2, 116.0 (d, *J* = 22.0 Hz), 115.7, 112.5. HRMS (ESI) calculated C_17_H_12_BrFNS [M+H]^+^ 359.9780, found 359.9859.

*(E)-4-(4-bromophenyl)-2-(2-methoxystyryl)thiazole (**30**).* Yellow solid, yield 54.3%, MP: 110–112 °C, purity: 99.0%. ^1^H NMR (500 MHz, CDCl_3_) *δ* 7.83 (d, *J* = 8.5 Hz, 2H), 7.78 (d, *J* = 16.0 Hz, 1H), 7.60 (dd, *J* = 7.5, 1.5 Hz, 1H), 7.57 (d, *J* = 8.5 Hz, 2H), 7.46 (d, *J* = 16.0 Hz, 1H), 7.39 (s, 1H), 7.36–7.31 (m, 1H), 7.01–6.97 (m, 1H), 6.95 (d, *J* = 8.5 Hz, 1H), 3.94 (s, 3H; ^13^C NMR (125 MHz, CDCl_3_) *δ* 168.1, 157.6, 154.8, 133.4, 131.9, 130.3, 128.0, 127.8, 124.6, 122.2, 120.9, 112.3, 111.1, 55.6. HRMS (ESI) calculated C_18_H_15_BrNOS [M+H]^+^ 371.9979, found 372.0064.

*(E)-4-(4-bromophenyl)-2-(3-methoxystyryl)thiazole (**31**).* Yellow solid, yield 57.8%, MP: 112–113 °C, purity: 98.8%. ^1^H NMR (500 MHz, CDCl_3_) *δ* 7.82 (s, 2H), 7.58 (d, *J* = 8.5 Hz, 2H), 7.45 (d, *J* = 16.0 Hz, 1H), 7.42 (s, 1H), 7.39–7.30 (m, 1H), 7.37 (d, *J* = 16.5 Hz, 1H), 7.17 (d, *J* = 7.5 Hz, 1H), 7.12–7.09 (m, 1H), 6.91 (dd, *J* = 8.5, 2.5 Hz, 1H), 3.87 (s, 3H); ^13^C NMR (125 MHz, CDCl_3_) *δ* 167.0, 160.0, 155.1, 137.1, 134.8, 133.3, 131.9, 129.9, 128.0, 122.3, 121.7, 119.9, 115.0, 112.6, 112.1, 55.3. HRMS (ESI) calculated C_18_H_15_BrNOS [M+H]^+^ 371.9979, found 372.0062.

*(E)-4-(4-bromophenyl)-2-(4-methoxystyryl)thiazole (**32**).* Yellow solid, yield 63.1%, MP: 189–191 °C, purity: 98.3%. ^1^H NMR (500 MHz, CDCl_3_) *δ* 7.85–7.80 (m, 2H), 7.57 (dd, *J* = 9.0, 2.0 Hz, 2H), 7.54–7.50 (m, 2H), 7.43 (d, *J* = 16.0 Hz, 1H), 7.37 (s, 1H), 7.26 (d, *J* = 16.0 Hz, 1H), 6.94 (d, *J* = 8.5 Hz, 2H), 3.86 (s, 3H); ^13^C NMR (125 MHz, CDCl_3_) δ 167.5, 160.4, 154.8, 134.5, 133.4, 131.8, 128.6, 128.4, 128.0, 122.1, 119.3, 114.4, 112.0, 55.4, 29.7. HRMS (ESI) calculated C_18_H_15_BrNOS [M+H]^+^ 371.9979, found 372.0062.

#### 3.1.7. Synthesis of Compounds **33**–**35**

BBr3 (5.01 g, 20 mmol) was added dropwise to a cold solution (−5 to 0 °C) of (*E*)-4-(4-bromophenyl)-2-(2-methoxystyryl)thiazole **30** (1.86 g, 5 mmol) in 30 mL of anhydrous dichloromethane. The mixture was allowed to warm to room temperature and was stirred for 5 h. After that, the reaction was quenched using ice water, and extracted with ethyl acetate. The organic phase was washed with H_2_O and dried using anhydrous Na_2_SO_4_. After removal of the solvent under reduced pressure, the crude product was purified by preparative TLC (petroleum ether/ethyl acetate: 3/1) to afford **33** as a light-yellow solid. (E)-2-(2-(4-(4-bromophenyl)thiazol-2-yl)vinyl)phenol (**33**): 1.14 g, yield 63.6%, MP: 226–228 °C, purity: 99.1%. ^1^H NMR (500 MHz, DMSO-d_6_) *δ* 10.13 (s, 1H), 8.11 (s, 1H), 7.96 (d, *J* = 8.5 Hz, 2H), 7.75 (d, *J* = 16.0 Hz, 1H), 7.66 (m, 3H), 7.54 (d, *J* = 16.5 Hz, 1H), 7.22–7.16 (m, 1H), 6.94 (d, *J* = 8.0 Hz, 1H), 6.86 (t, *J* = 8.0 Hz, 1H); ^13^C NMR (125 MHz, DMSO-d_6_) *δ* 167.1, 155.8, 153.6, 133.2, 131.7, 130.1, 128.1, 127.9, 127.7, 122.1, 121.2, 120.7, 119.4, 116.0, 114.3. HRMS (ESI) calculated C_17_H_13_BrNOS [M+H]^+^ 357.9823, found 357.9904. Compounds **34** and **35** were synthesized according to the methods described above for the synthesis of **33**, using compounds **31** and **32**, respectively, as starting reactants. (E)-3-(2-(4-(4-bromophenyl)thiazol-2-yl)vinyl)phenol (**34**): 1.17 g, yield 65.3%, MP: 201–203 °C, purity: 98.8%. ^1^H NMR (500 MHz, DMSO-d_6_) *δ* 9.55 (d, *J* = 27.5 Hz, 1H), 8.10 (d, *J* = 44.5 Hz, 1H), 7.97 (d, *J* = 8.5 Hz, 1H), 7.89 (d, *J* = 6.5 Hz, 1H), 7.66 (d, *J* = 8.5 Hz, 1H), 7.62 (d, *J* = 9.0 Hz, 1H), 7.46 (d, *J* = 8.0 Hz, 1H), 7.22 (d, *J* = 15.5 Hz, 1H), 7.16 (d, *J* = 7.5 Hz, 1H), 7.04 (m, 1H), 6.95 (m, 1H), 6.82–6.78 (m, 1H); ^13^C NMR (125 MHz, DMSO-d_6_) *δ* 166.2, 163.5, 157.6, 153.7, 152.6, 136.9, 136.7, 135.4, 134.5, 133.1, 131.7, 129.8, 128.0, 119.2, 118.3, 116.3, 116.0, 115.6, 115.3, 114.8, 113.8. HRMS (ESI) calculated C_17_H_13_BrNOS [M+H]^+^ 357.9823, found 357.9909. (E)-4-(2-(4-(4-bromophenyl)thiazol-2-yl)vinyl)phenol (**35**): 1.34 g, yield 74.9%, MP: 204–205 °C, purity: 99.0%. ^1^H NMR (500 MHz, DMSO-d_6_) *δ* 9.86 (s, 1H), 8.07 (s, 1H), 7.95 (d, *J* = 8.5 Hz, 2H), 7.65 (d, *J* = 8.5 Hz, 2H), 7.56 (d, *J* = 8.5 Hz, 2H), 7.46 (d, *J* = 16.5 Hz, 1H), 7.32 (d, *J* = 16.5 Hz, 1H), 6.82 (d, *J* = 8.5 Hz, 2H); ^13^C NMR (125 MHz, DMSO-d_6_) δ 166.9, 158.6, 153.5, 134.6, 133.3, 131.7, 128.5, 126.5, 121.2, 117.9, 115.8, 113.9. HRMS (ESI) calculated C_17_H_13_BrNOS [M+H]^+^ 357.9823, found 357.9909 (Figures S33–S35).

#### 3.1.8. Synthesis of Compounds **36** and **37**

Potassium carbonate (2.07 g, 15 mmol) was added to a solution of (*E*)-3-(2-(4-(4-bromophenyl)thiazol-2-yl)vinyl)phenol (**34**) (1.79 g, 5 mmol) in 20 mL of anhydrous acetone, and the mixture stirred vigorously under refluxing for 1 h. After that, a solution of prenyl bromide (0.90 g, 6 mmol) in 5 mL of anhydrous acetone was added dropwise, and then stirred under refluxing for an additional 3 h. The mixture was cooled to room temperature, then filtrated and evaporated; the residue was purified by preparative TLC (petroleum ether/ethyl acetate: 5/1) to give **36** as a light-yellow solid. (E)-4-(4-bromophenyl)-2-(3-((3-methylbut-2-en-1-yl)oxy)styryl)thiazole (**36**): 1.33 g, yield 62.4%, MP: 122–123 °C, purity: 98.7%. ^1^H NMR (500 MHz, CDCl_3_) *δ* 7.84–7.81 (m, 2H), 7.60–7.56 (m, 2H), 7.44 (d, *J* = 16.0 Hz, 1H), 7.41 (s, 1H), 7.34 (d, *J* = 13.5 Hz, 1H), 7.33–7.29 (m, 1H), 7.16 (d, *J* = 7.5 Hz, 1H), 7.13–7.12 (m, 1H), 6.94–6.91 (m, 1H), 5.57–5.52 (m, 1H), 4.57 (d, *J* = 7.0 Hz, 2H), 1.84 (s, 3H), 1.79 (s, 3H); ^13^C NMR (125 MHz, CDCl_3_) *δ* 167.0, 159.3, 155.0, 138.4, 137.0, 134.9, 133.2, 131.9, 129.8, 128.0, 122.2, 121.6, 119.9, 119.5, 115.8, 112.6, 64.8, 25.9, 18.3. HRMS (ESI) calculated C_22_H_21_BrNOS [M+H]^+^ 426.0449, found 426.0527. Compound **37** was synthesized according to the above method using compound **35** as the starting reactant. (E)-4-(4-bromophenyl)-2-(4-((3-methylbut-2-en-1-yl)oxy)styryl)thiazole (**37**): 1.24 g, yield 58.1%, MP: 134–135 °C, purity: 99.2%. ^1^H NMR (500 MHz, CDCl_3_) *δ* 7.84–7.80 (m, 2H), 7.59–7.55 (m, 2H), 7.51 (d, *J* = 8.5 Hz, 2H), 7.43 (d, *J* = 16.0 Hz, 1H), 7.37 (s, 1H), 7.23 (d, *J* = 16.0 Hz, 1H), 6.95 (d, *J* = 8.5 Hz, 2H), 5.57–5.48 (m, 1H), 4.56 (d, *J* = 6.5 Hz, 2H), 1.83 (s, 3H), 1.78 (s, 3H); ^13^C NMR (125 MHz, CDCl_3_) *δ* 167.6, 159.7, 154.8, 138.5, 134.6, 133.4, 131.8, 128.6, 128.3, 128.0, 122.1, 119.3, 115.1, 112.0, 64.9, 25.9, 18.2. HRMS (ESI) calculated C_22_H_21_BrNOS [M+H]^+^ 426.0449, found 426.0527 (Figures S36 and S37).

### 3.2. Bioassay of Top1-Mediated Relaxation Assay

The compounds were tested for their Top1 inhibitory activity using a Top1-mediated relaxation assay [35]. Briefly, the reaction mixture (20 μL) with 0.5 μg of supercoiled pBR322 DNA in relaxation buffer (10 mM Tris-HCl, pH 7.5, 50 mM KCl, 5 mM MgCl_2_, 15 μg/mL BSA, 40 μg/mL DTT) was incubated with 1 unit of calf thymus Top1 in the absence or in the presence of the compound for 30 min at 37 °C. Then, the reaction solution was added with 6 × loading buffer (4 μL) and was analyzed using a 0.8% agarose gel in TBE buffer at 4.6 V/cm for 1.5 h. Gel was stained with 1 × GelRed for 30 min and subsequently visualized with a UV transilluminator.

### 3.3. Molecular Modeling

The PDB file for the X-ray crystal structure of Top1 was obtained using the protein data bank code 1K4T [36]. The ternary complex ligand centroid coordinates for docking were defined using the ligand in the Top1−DNA−topotecan crystal structure as the center of the binding pocket. The protein structure was cleaned and inspected for errors and missing residues, hydrogens were added and the water molecules and the ligand were deleted. The compound to be modeled (compound **8)** was constructed and optimized using ChemDraw, saved in SDF file formats and corrected using MOE software. Hydrogens were added, and the ligand was minimized by the conjugate gradient method using the MMFF94x force field with MMFF94 charges, a distance-dependent dielectric function and a 0.01 kcal.mol^−1^ Ǻ^−1^ energy gradient convergence criterion. Induced fit was used for docking with the default parameters. The top 30 docking poses of the ligand were inspected visually following the docking runs. The highest-ranked pose was merged into the crystal structure. Energy minimizations were performed for the highest-ranked pose for the ligand. The AMBER force field was utilized within the MOE software (Version 2014; https://www.chemcomp.com, accessed on 9 January 2022) for energy minimization.

### 3.4. Cell Culture and MTT Assay

The cells were cultured on RPMI 1640 Medium at 37 °C in a humidified atmosphere with 5% CO_2_. The cytotoxicities of the target compounds were evaluated through MTT assay against two human cancer cell lines (human breast cancer MCF-7 and human colon cancer HCT116). For the compound treatment experiments, the cancer cells were treated with the compounds (pre-dissolved in DMSO) via a five-dose assay ranging from 10^−8^ to 10^−4^ M. After incubation for 72 h at 37 °C, MTT solution (20 μL, 2.5 mg/mL) in PBS (PBS without MTT as the blank) was added to each well of the culture plate. After incubation for 4 h, the formazan crystal formed in the well was dissolved in 100 μL of DMSO for an optical density of 570 nm [37]. The IC_50_ value was calculated using nonlinear regression analysis (GraphPad Prism). Every experiment was conducted three times, independently.

## 4. Conclusions

In summary, a series of thiazole-based stilbene analogs were designed and synthesized. Top1-mediated assays revealed that the synthesized compounds could act as Top1 inhibitors, and compound **8** exhibited the highest Top1 inhibitory activity (++++), comparable to that of CPT. A possible binding mode of compound **8** with Top1–DNA complex was further provided by molecular docking. The synthesized compounds were also evaluated for their cytotoxic activities by MTT assay, and the majority of them showed high cytotoxicity against MCF-7 and HCT116 cancer cell lines, with IC_50_ values at micromolar concentrations. Compound **8** exhibited the highest cytotoxicity against MCF-7, with an IC_50_ value of 0.78 μM, and compound **11** showed the highest cytotoxicity against HCT116 with an IC_50_ value of 0.62 μM. In addition, the preliminary structure–activity relationship was also discussed. These results indicate that the thiazole-based stilbene scaffold is a novel chemotype for developing new Top1 inhibitors with high cytotoxicity, which offers potential for the discovery of anticancer drugs.

## Figures and Tables

**Table 1:** Top1 inhibitory activities of synthesized compounds.

Comp.	X	R	Overall Yield (%)	Relaxation Assay ^a^	Comp.	X	R	Overall Yield (%)	Relaxation Assay ^a^
6	F	H	20.3	++	22	Cl	3-Me	28.1	++
7	F	2-Me	24.8	++	23	Cl	4-*tert*-Bu	25.9	+
8	F	3-Me	25.0	++++	24	Cl	2-OMe	33.9	++
9	F	4-Me	24.7	+	25	Cl	2-Cl	31.9	+
10	F	4-CF_3_	26.4	+++	26	Br	3-Me	29.1	++
11	F	4-*tert*-Bu	24.5	+++	27	Br	4-CF_3_	25.2	++
12	F	2-OMe	22.2	++	28	Br	4-*tert*-Bu	27.2	+
13	F	3-OMe	22.9	+++	29	Br	4-F	20.8	++
14	F	4-OMe	25.8	+	30	Br	2-OMe	24.9	+
15	F	4-F	21.4	+++	31	Br	3-OMe	26.5	+
16	F	2-Cl	25.2	++	32	Br	4-OMe	29.0	+
17	F	4-Cl	21.2	+	33	Br	2-OH	15.8	+
18	F	2-Br	19.8	++	34	Br	3-OH	17.3	+
19	F	3-Br	21.2	+++	35	Br	4-OH	21.7	+
20	F	2,4-di-Cl	19.6	+	36	Br	3-O-prenyl	10.8	++
21	Cl	2-Me	30.2	+	37	Br	4-O-prenyl	12.6	+

^a^ Top1 inhibitory activity was semi-quantitatively expressed relative to CPT at 50 μM as follows: ++++, more than 80% activity; +++, between 40% and 79% activity; ++, between 10% and 39% activity; +, less than 10% activity. Every experiment was repeated at least twice, independently.

**Table 2:** Cytotoxicity of synthesized compounds.

Comp.	MCF-7	HCT116	HEK293T	Comp.	MCF-7	HCT116	HEK293T
	Cytotoxicity (IC_50_, μM)				Cytotoxicity (IC_50_, μM)		
6	22.16 ± 5.01	5.78 ± 1.89	- ^b^	23	7.22 ± 0.40	2.87 ± 0.26	18.67 ± 1.37
7	31.42 ± 4.21	6.47 ± 0.62	-	24	>100	49.42 ± 3.49	-
8	0.78 ± 0.12	1.48 ± 0.16	10.15 ± 0.68	25	48.52 ± 6.67	50.98 ± 3.70	-
9	65.52 ± 3.11	21.87 ± 4.29	-	26	10.79 ± 2.42	2.09 ± 0.15	-
10	17.27 ± 1.62	22.60 ± 2.41	-	27	24.27 ± 5.62	34.90 ± 5.66	-
11	6.31 ± 0.51	0.62 ± 0.09	12.02 ± 2.45	28	3.78 ± 1.66	1.39 ± 0.16	21.78 ± 3.80
12	49.53 ± 9.08	17.80 ± 1.16	-	29	>100	63.25 ± 6.17	-
13	9.85 ± 0.47	4.99 ± 0.80	25.16 ± 1.52	30	>100	>100	-
14	>100	>100	-	31	45.82 ± 4.47	40.30 ± 7.80	-
15	4.14 ± 0.40	10.22 ± 1.07	-	32	>100	>100	-
16	15.20 ± 1.18	8.42 ± 1.10	-	33	39.18 ± 1.79	4.15 ± 0.76	-
17	43.27 ± 1.42	27.09 ± 0.41	-	34	22.79 ± 1.40	9.99 ± 0.19	-
18	20.04 ± 2.07	12.29 ± 1.10	-	35	38.01 ± 5.82	2.86 ± 0.46	-
19	11.45 ± 1.74	6.95 ± 0.33	-	36	>100	>100	-
20	72.43 ± 2.15	54.79 ± 0.73	-	37	>100	>100	-
21	39.51 ± 1.06	41.72 ± 1.92	-	CPT ^a^	0.34 ± 0.026	0.012 ± 0.001	0.10 ± 0.003
22	>100	>100	-	vp-16 ^a^	24.68 ± 3.12	18.95 ± 1.35	-

^a^ Positive control; ^b^ not tested.

## Data Availability

The data presented in this study is available in the article or Supplementary Materials.

## Supplementary Materials

The ^1^H NMR and ^13^C NMR spectra of the title compounds can be downloaded.
