# Supplementary material for: Design, Synthesis and Cytotoxicity of Thiazole-Based Stilbene Analogs as Novel DNA Topoisomerase IB Inhibitors
Source: Molecules. 2022 Feb 2;27(3):1009. doi: 10.3390/molecules27031009 (PMC8838847; doi:10.3390/molecules27031009)
Supplement: Supplementary file 1 [file molecules-27-01009-s001.zip › molecules-1531971-supplementary.pdf]

molecules

Supporting Information

Design, Synthesis and Cytotoxicity of Thiazole-based Stilbene Analogs as Novel DNA Topoisomerase IB Inhibitors

Jin-Chuan Liu, Bo Chen, Jia-Lin Yang, Jian-Quan Weng *, Qian Yu * and De-Xuan Hu

Table of Contents

1. ^{1}H-NMR spectrum of intermediates **5b** and **5b'** S2
2. ^{1}H-NMR and ^{13}C NMR spectrum of compounds **6~37** S3

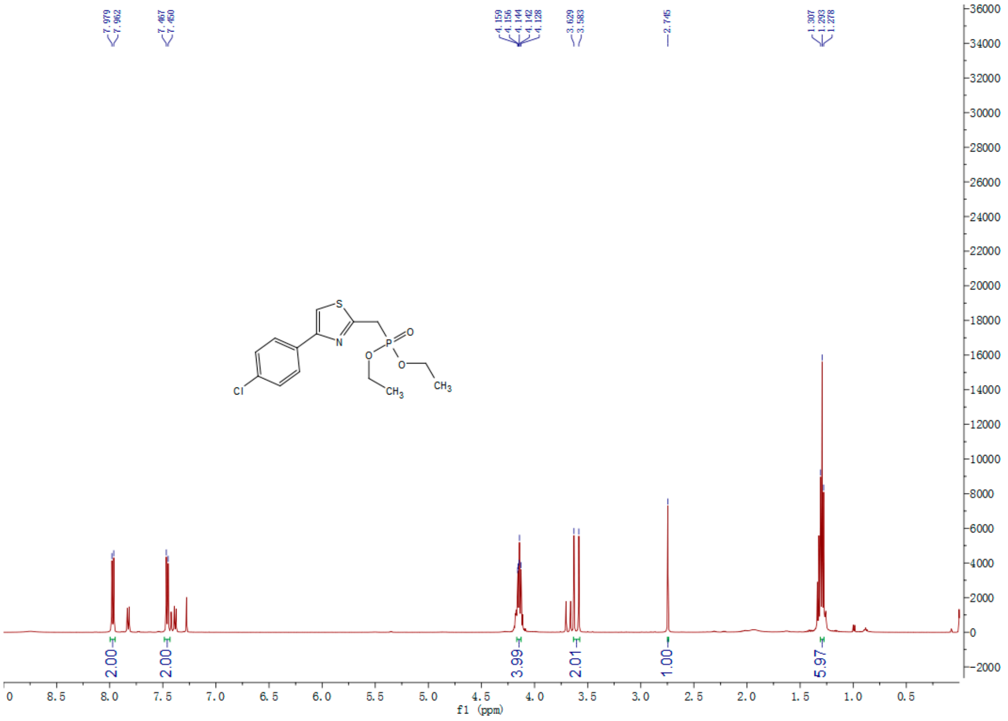

Figure S1. ^{1}H NMR of 5b.

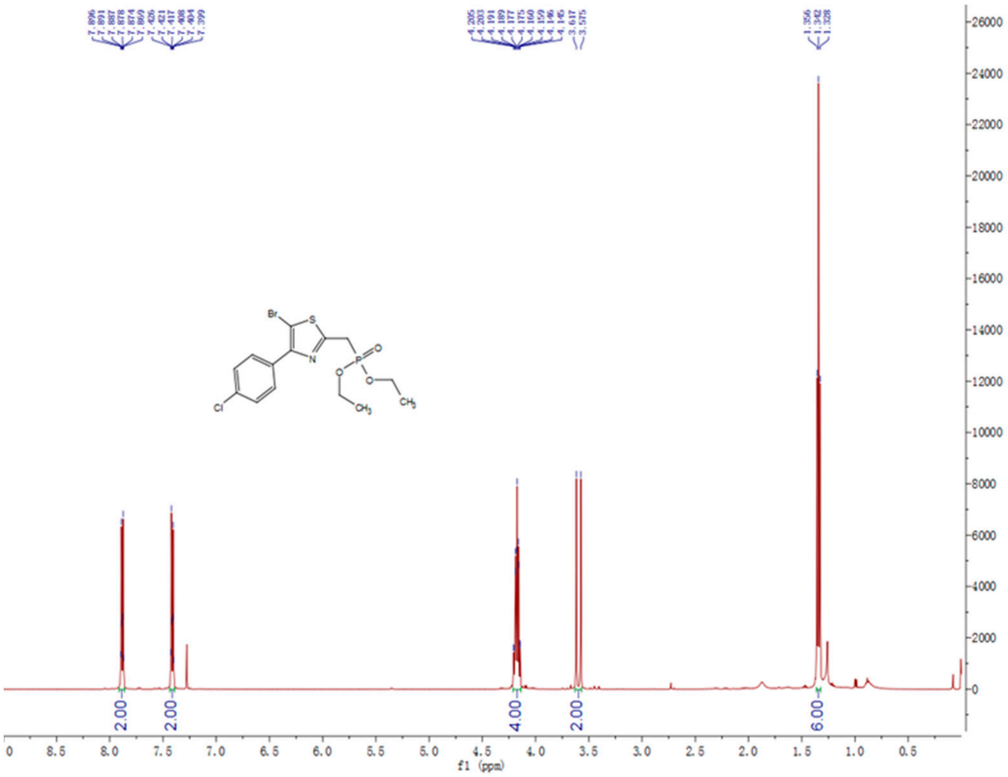

Figure S2. ^{1}H NMR of 5b′.

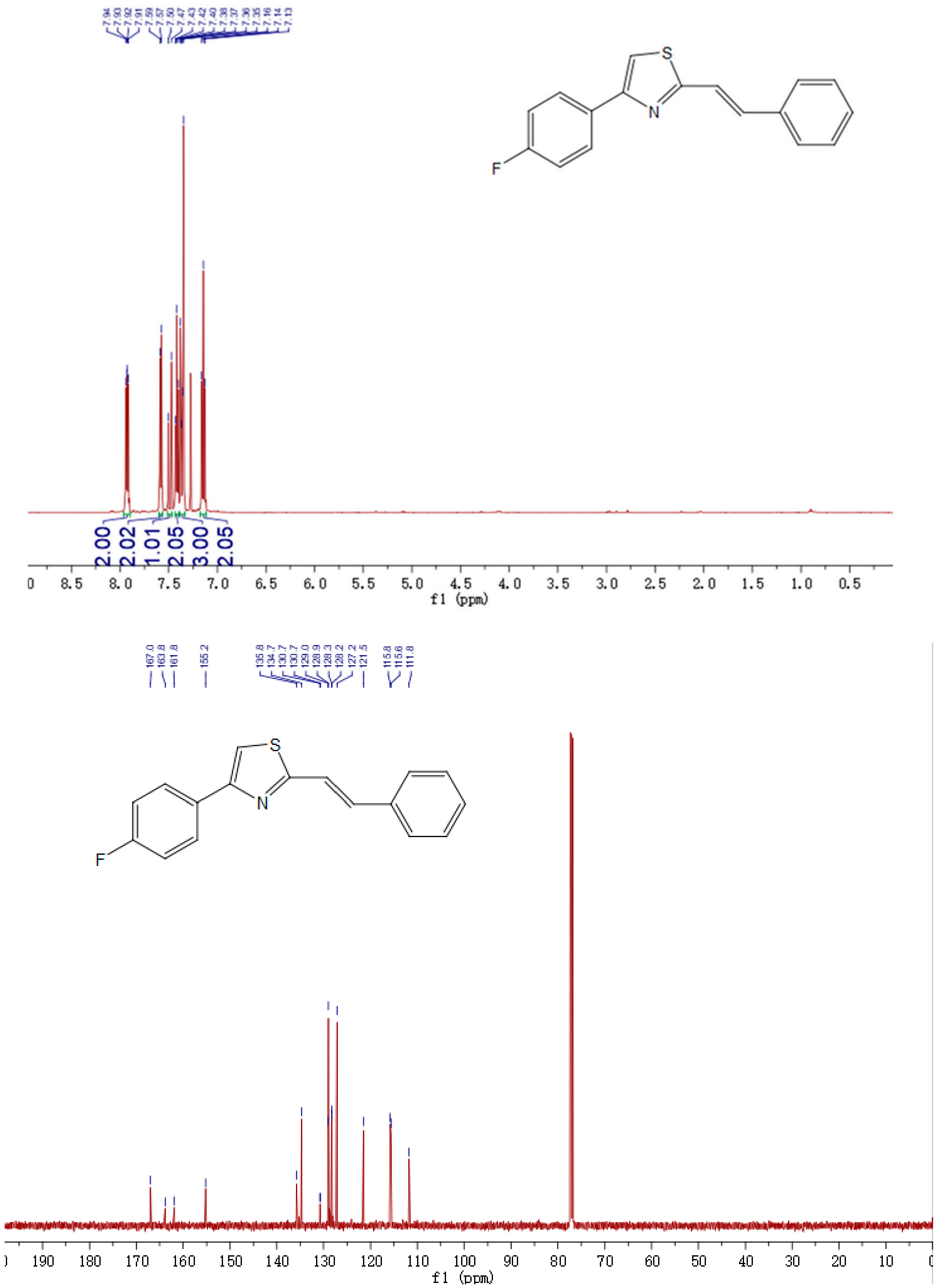

Figure S3. ^{1}H NMR and ^{13}C NMR of **6.**

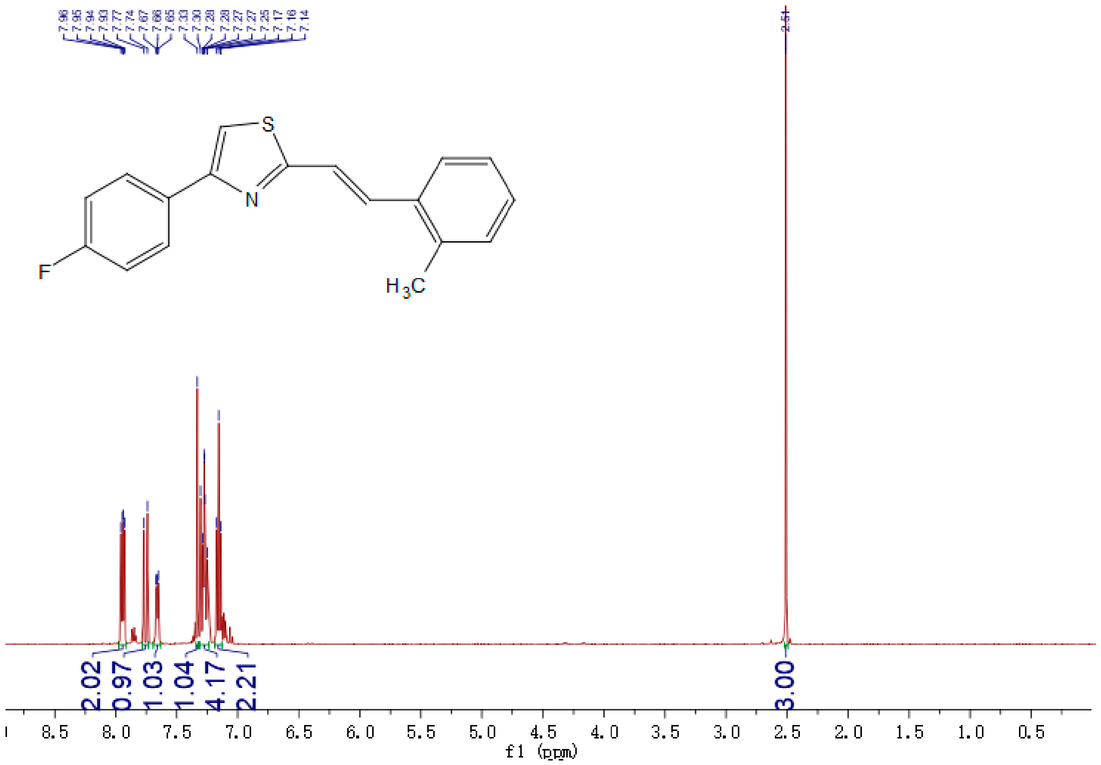

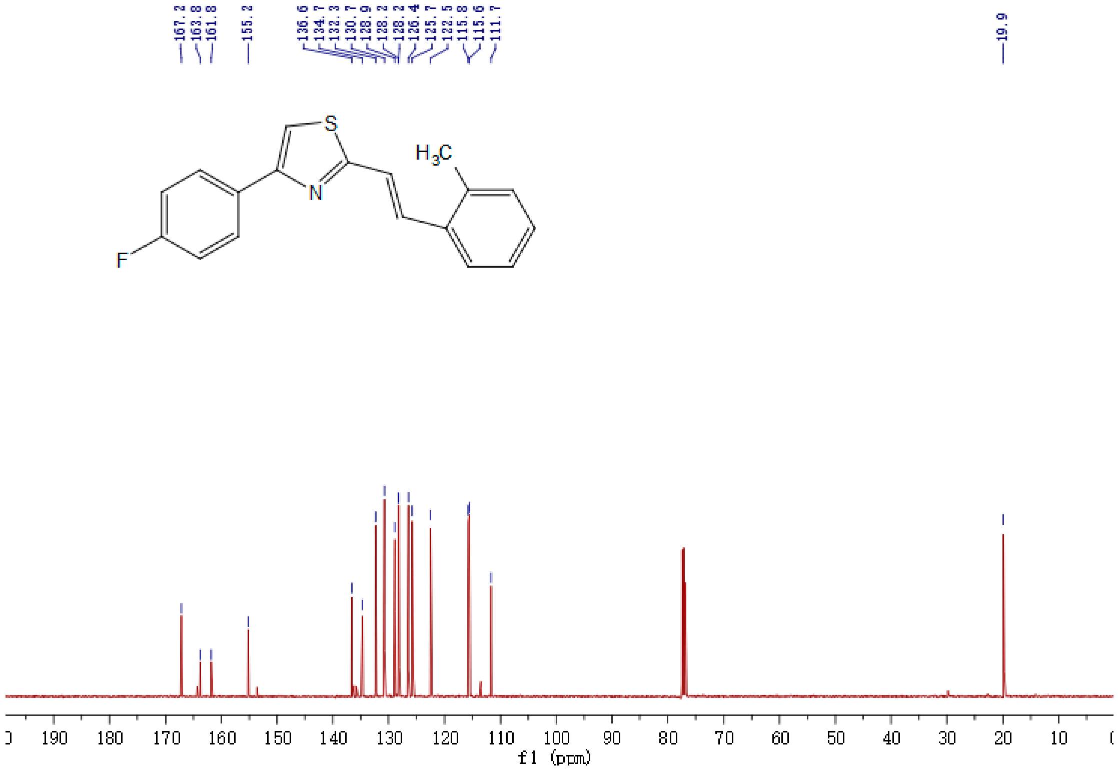

Figure S4. ^1H NMR and ^{13}C NMR of **7.**

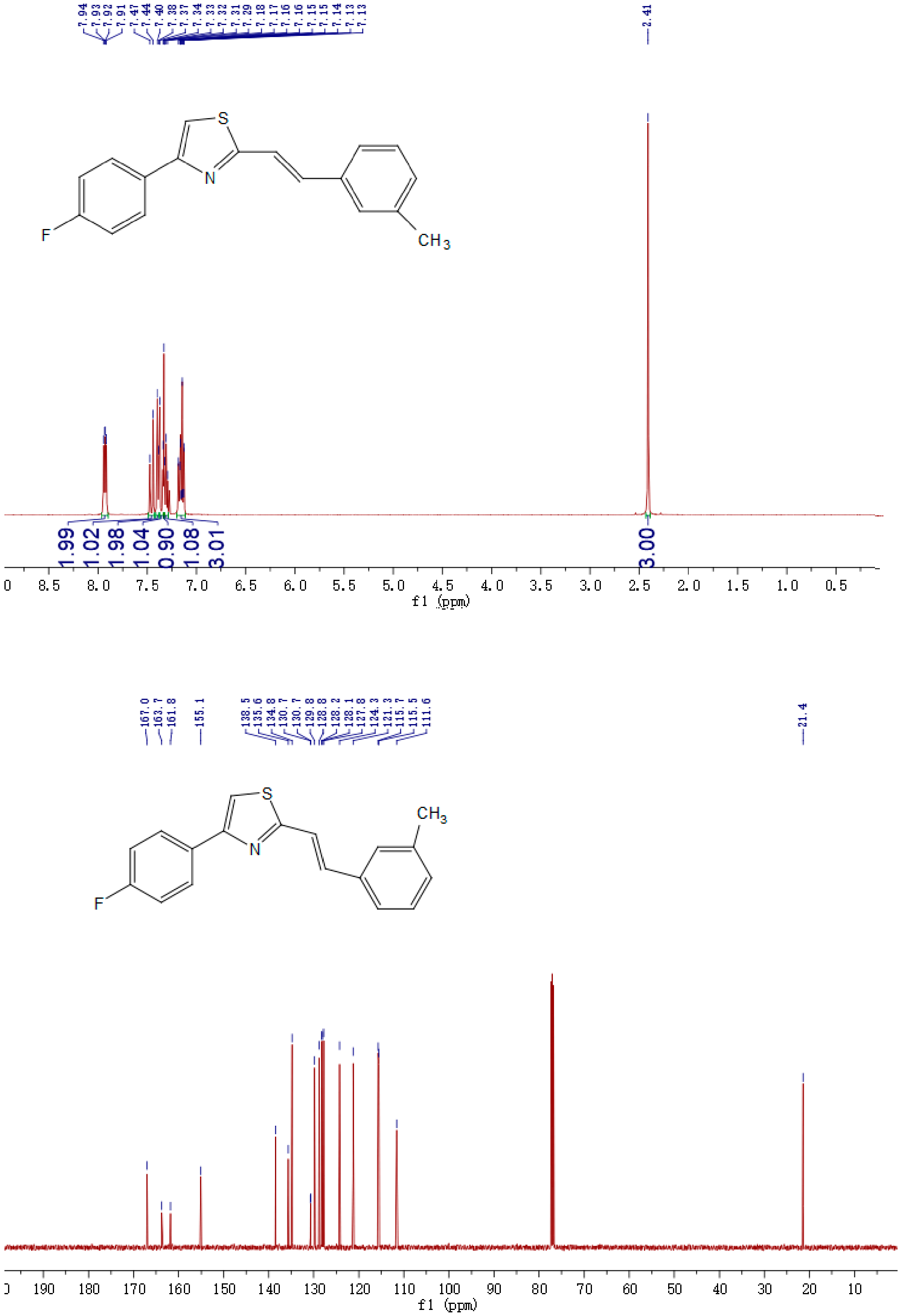

Figure S5. ¹H NMR and ¹³C NMR of **8.**

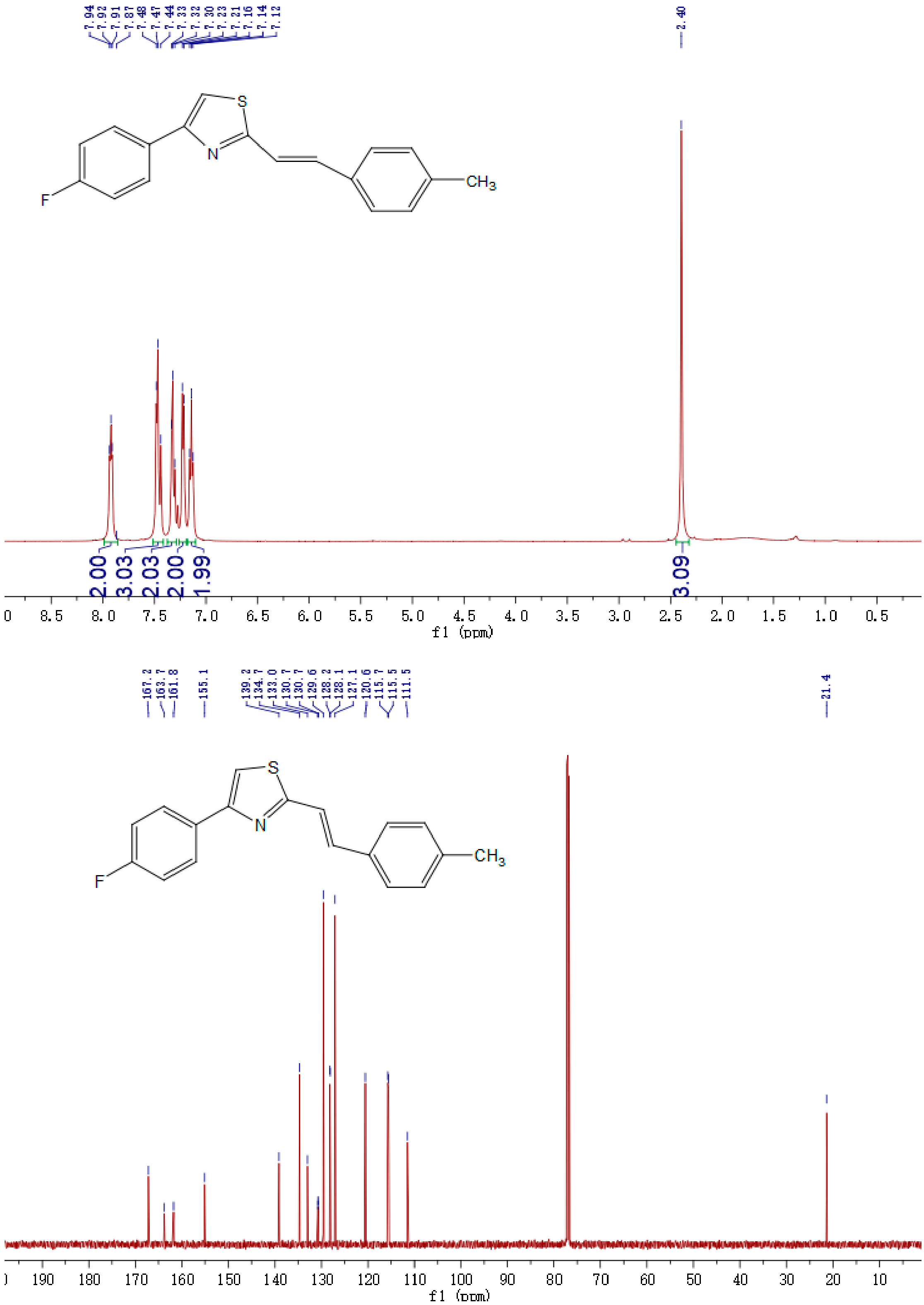

Figure S6. ^{1}H NMR and ^{13}C NMR of **9.**

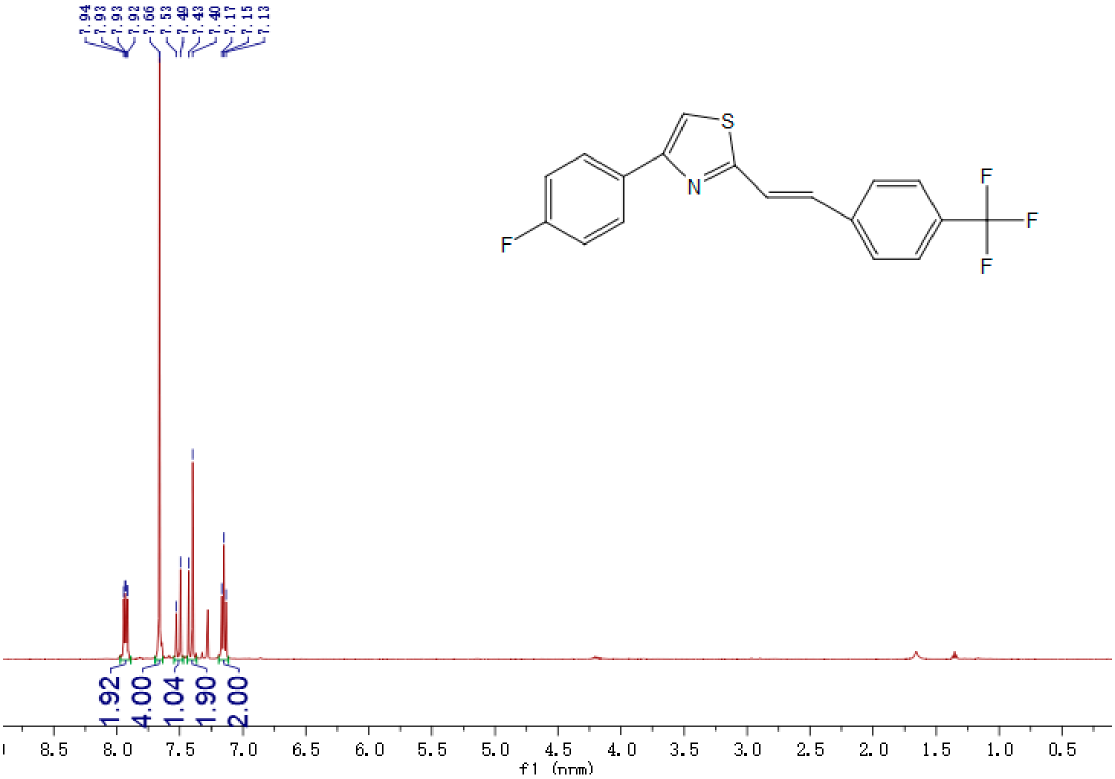

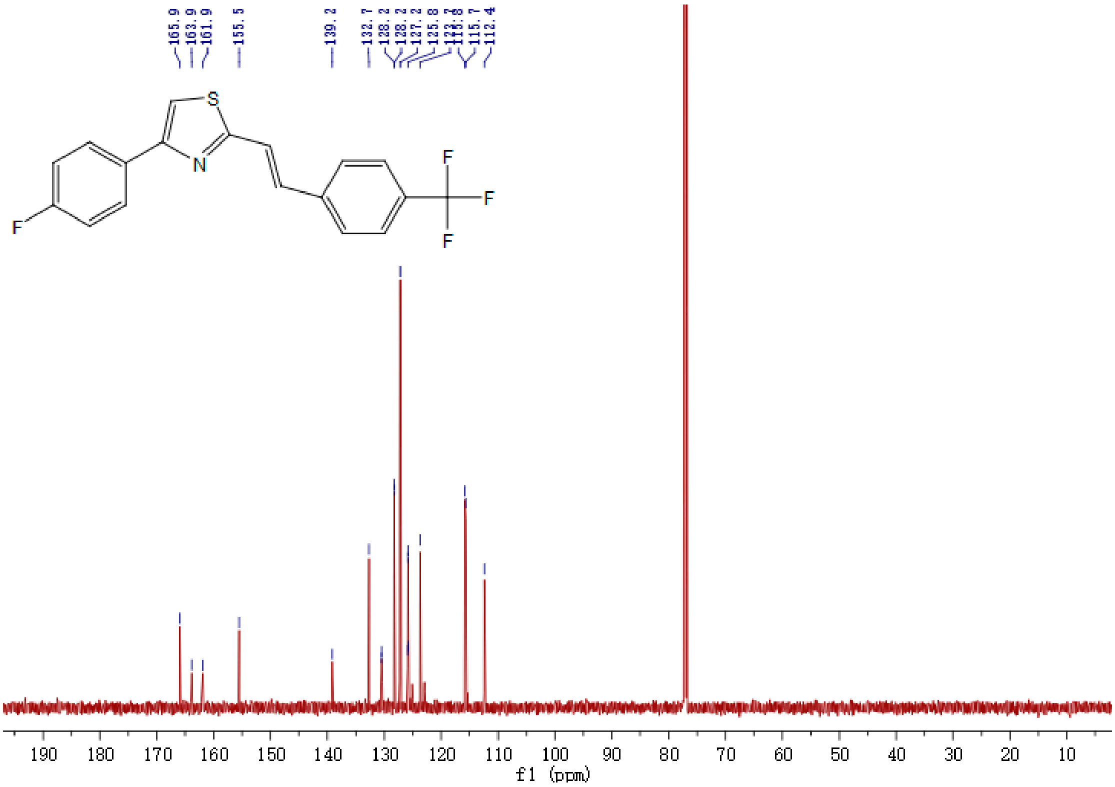

Figure S7. ^{1}H NMR and ^{13}C NMR of **10.**

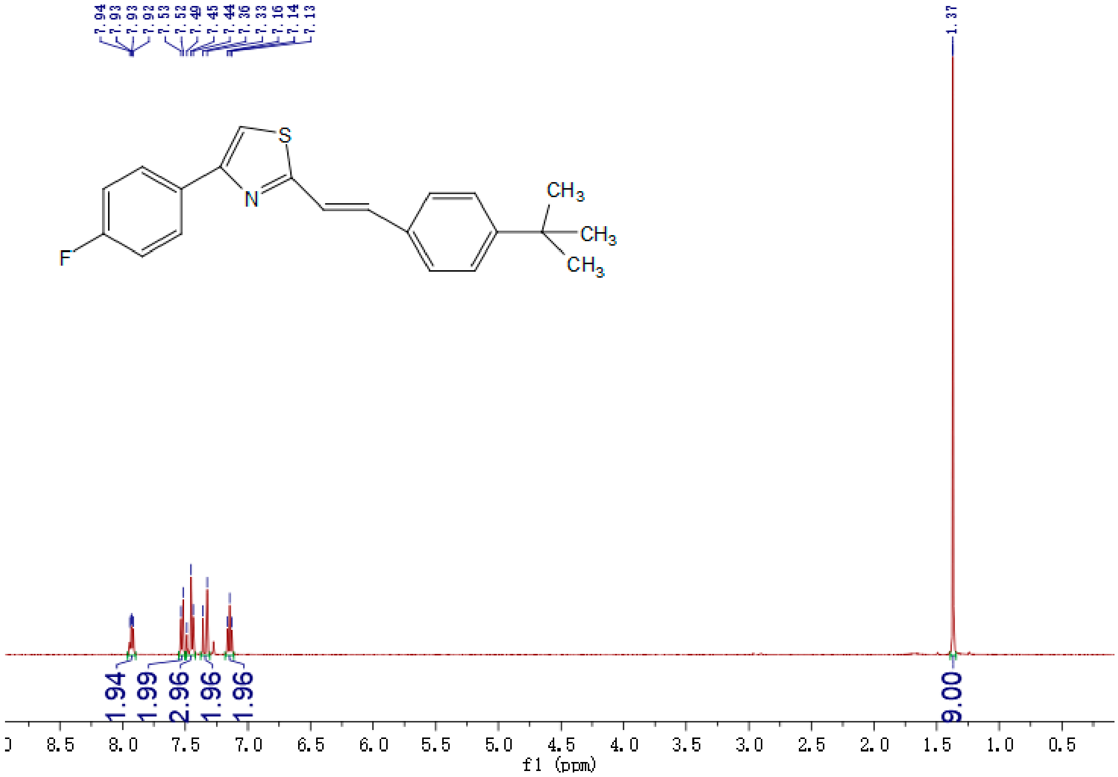

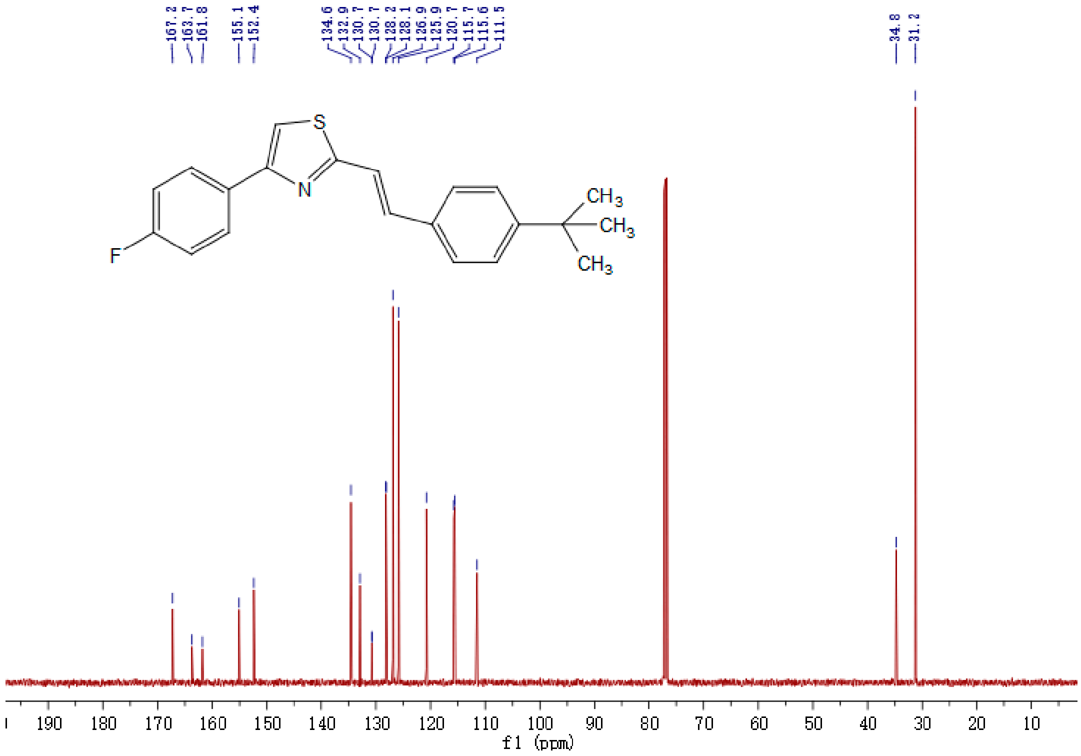

Figure S8. ^{1}H NMR and ^{13}C NMR of **11.**

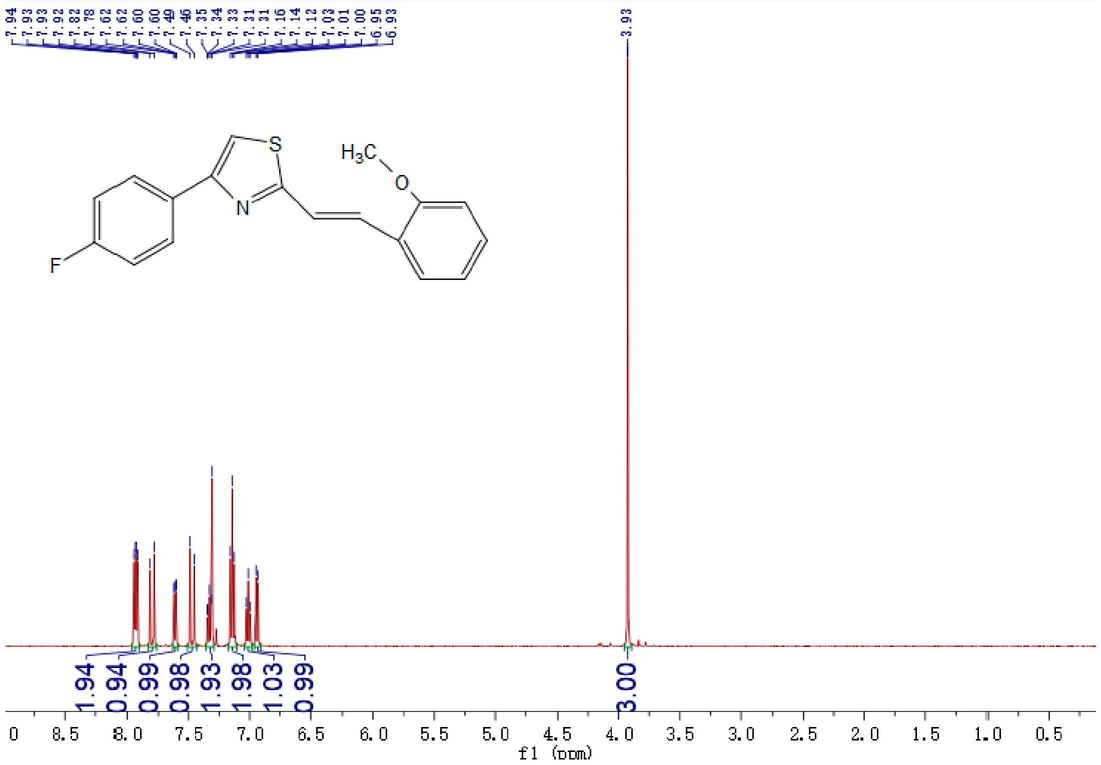

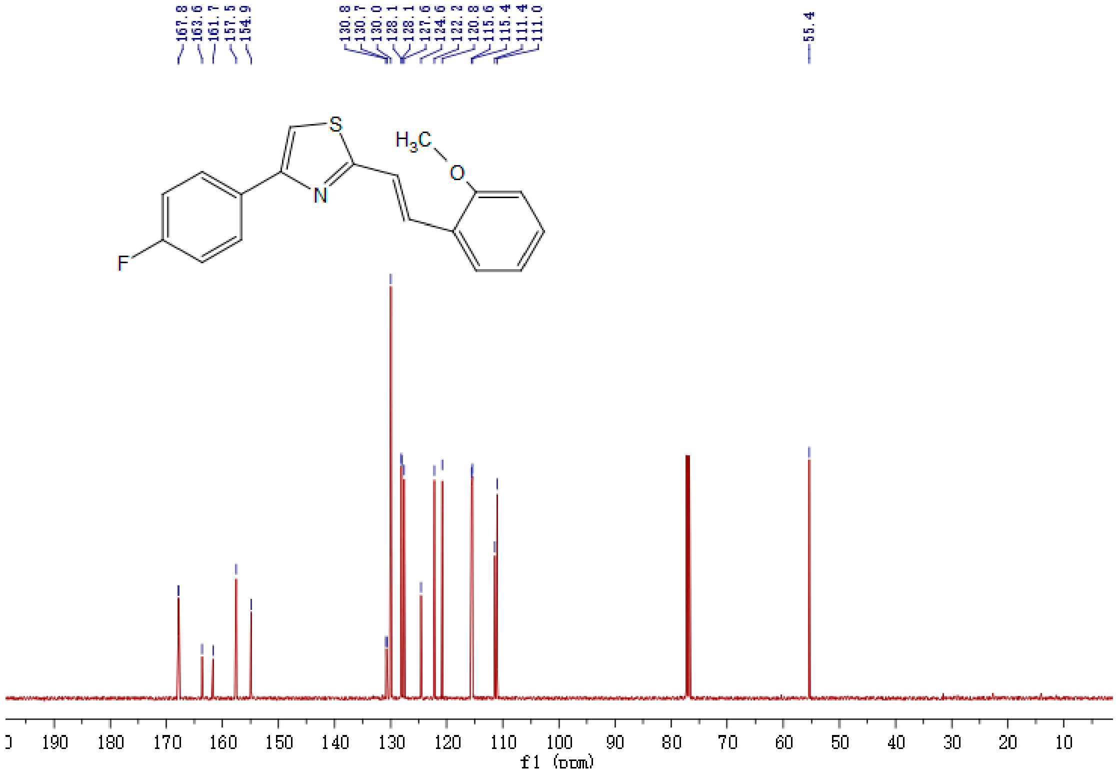

Figure S9. ^{1}H NMR and ^{13}C NMR of **12.**

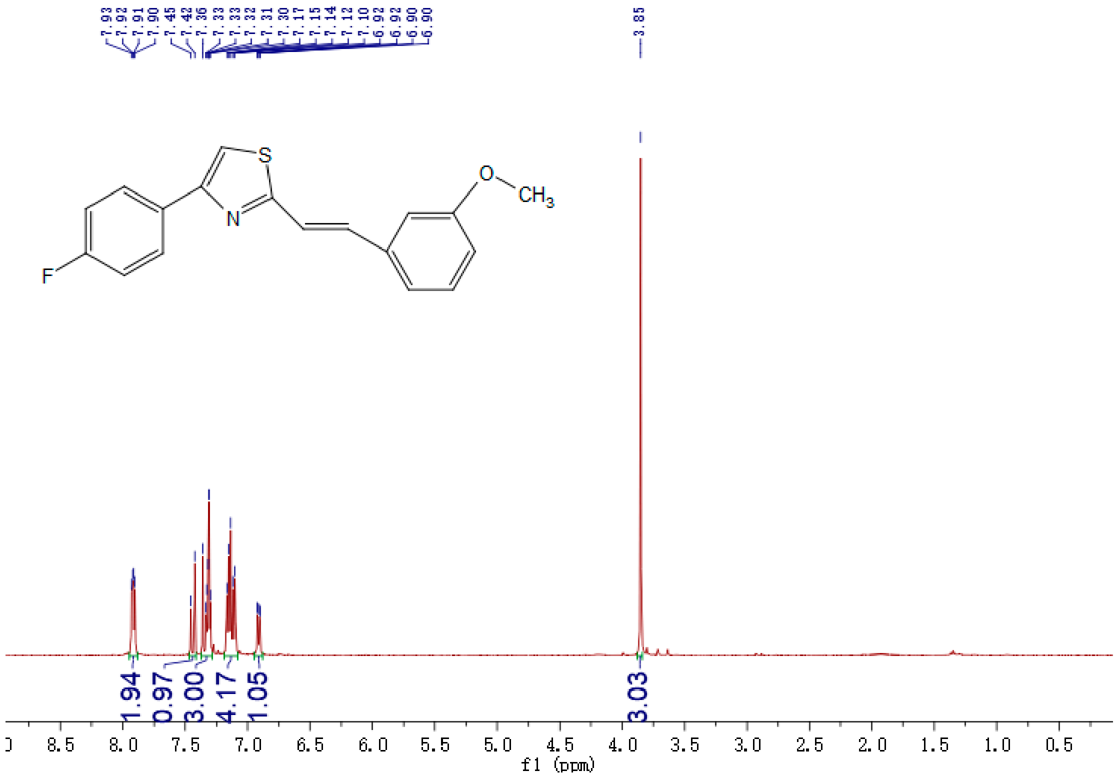

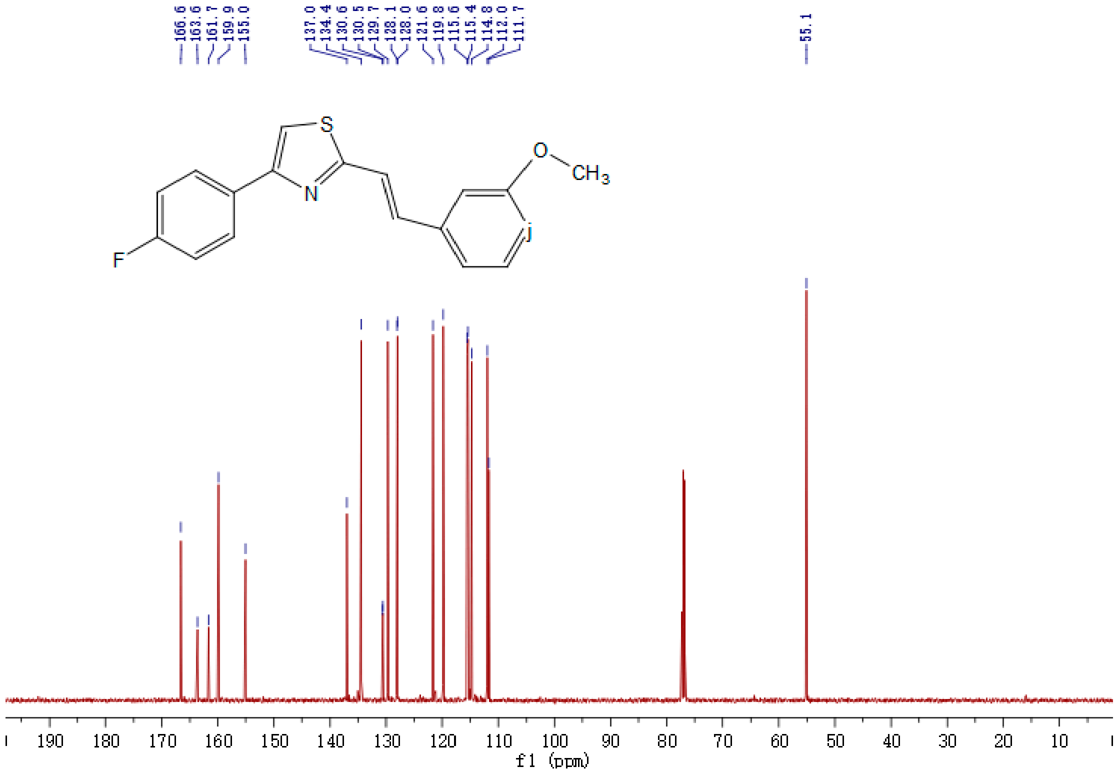

Figure S10. ^{1}H NMR and ^{13}C NMR of **13.**

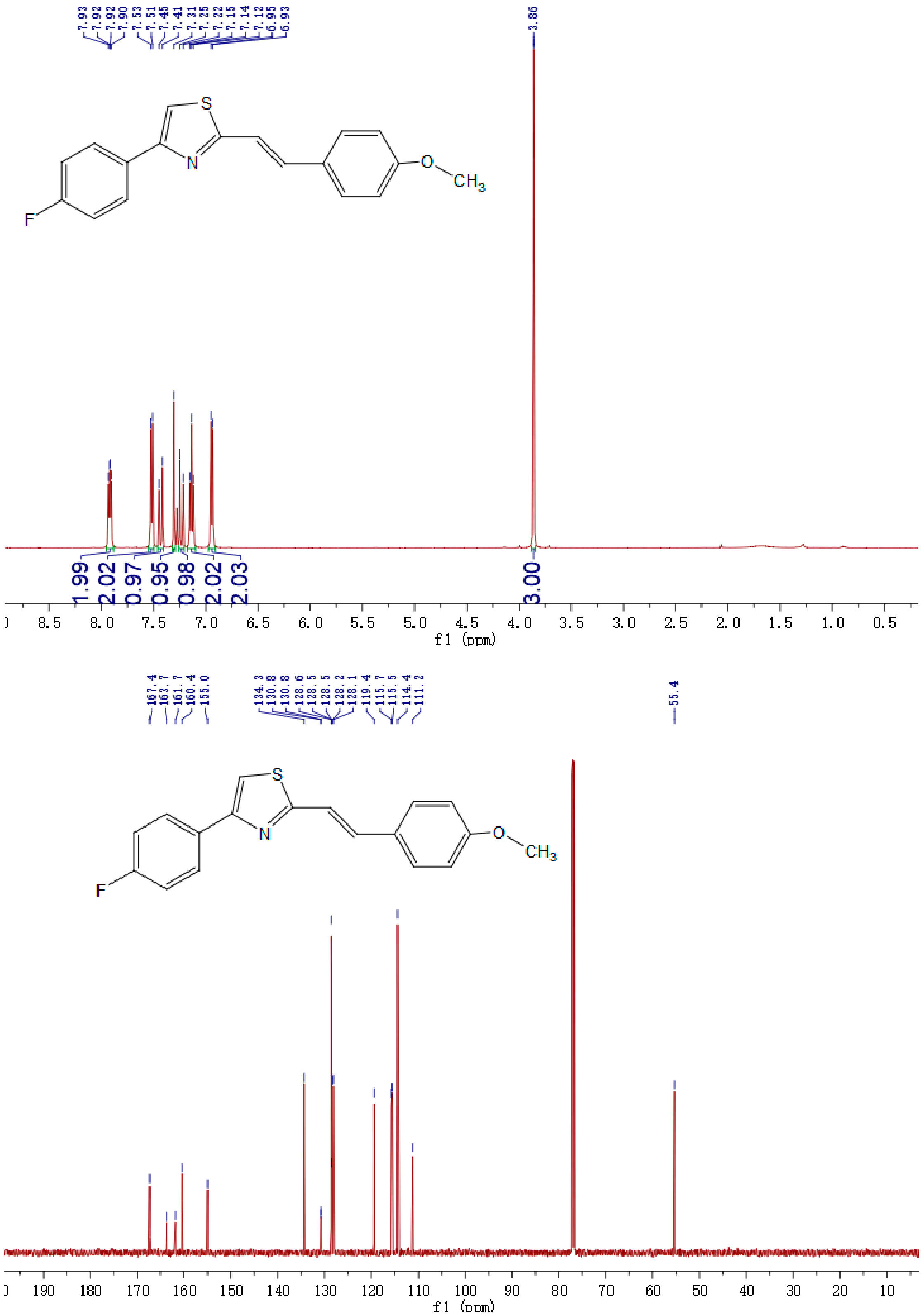

Figure S11. ^{1}H NMR and ^{13}C NMR of **14.**

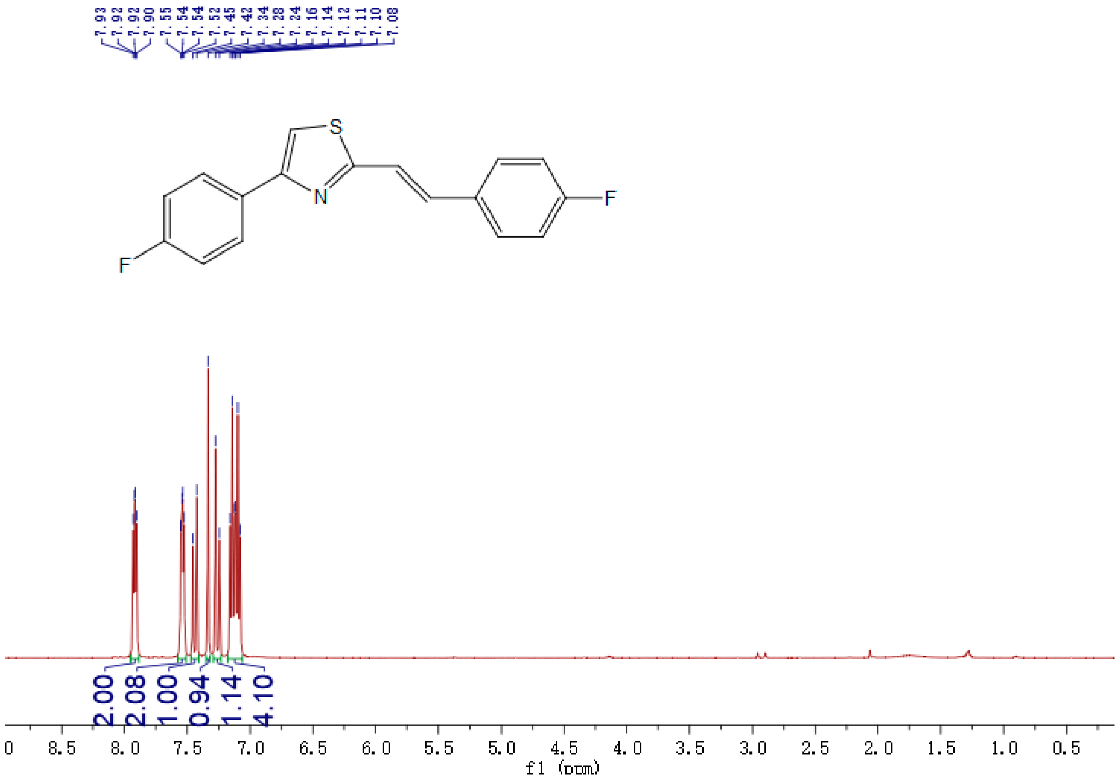

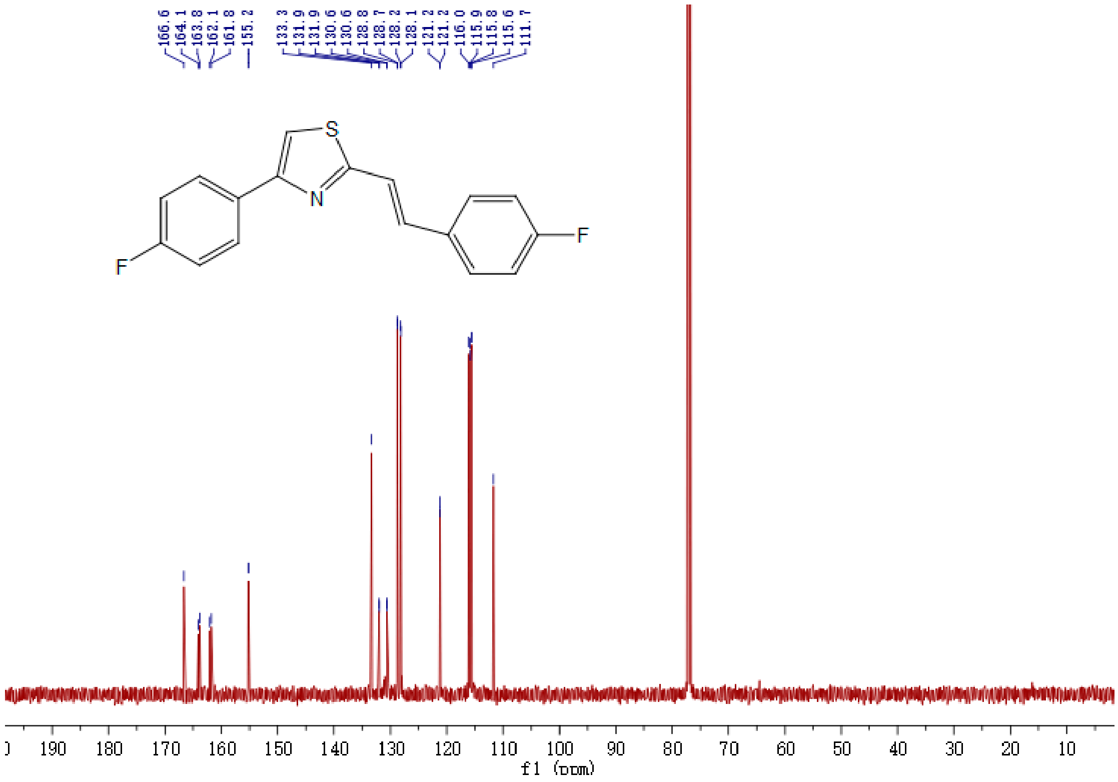

Figure S12 ^{1}H NMR and ^{13}C NMR of **15**

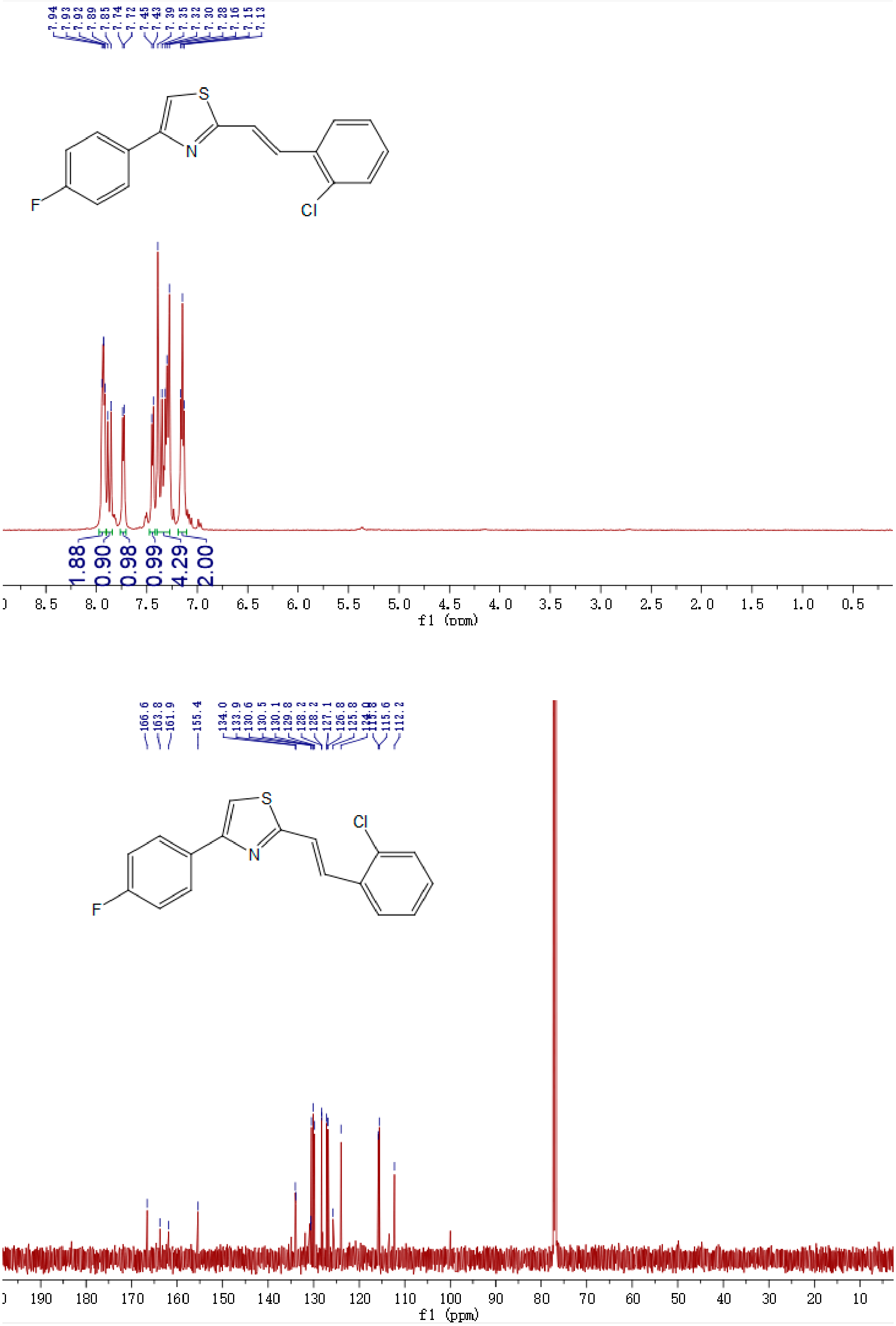

Figure S13. ^{1}H NMR and ^{13}C NMR of **16.**

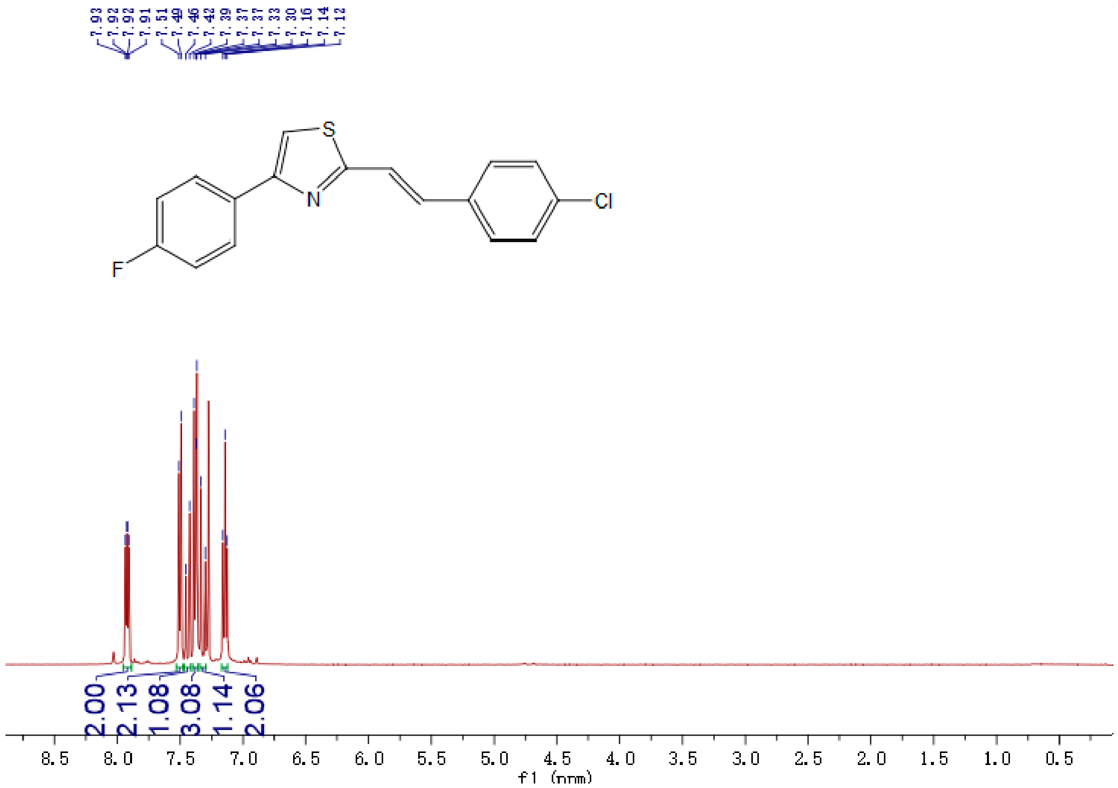

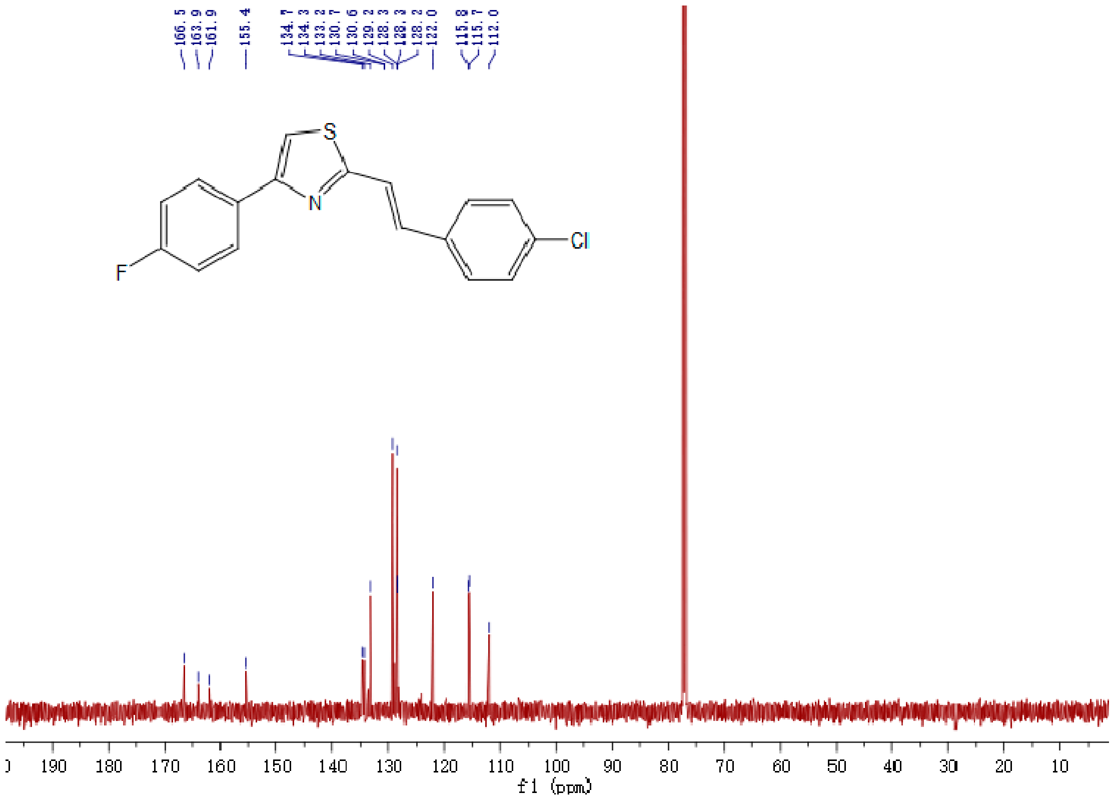

Figure S14. ^{1}H NMR and ^{13}C NMR of **17.**

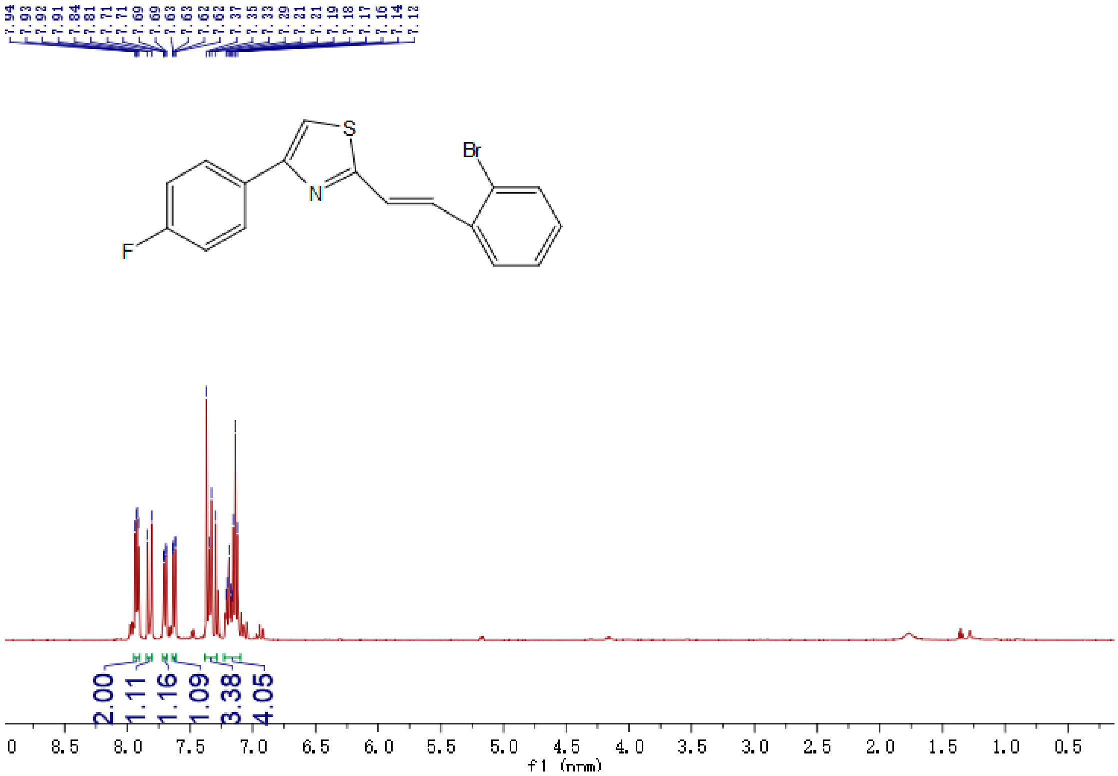

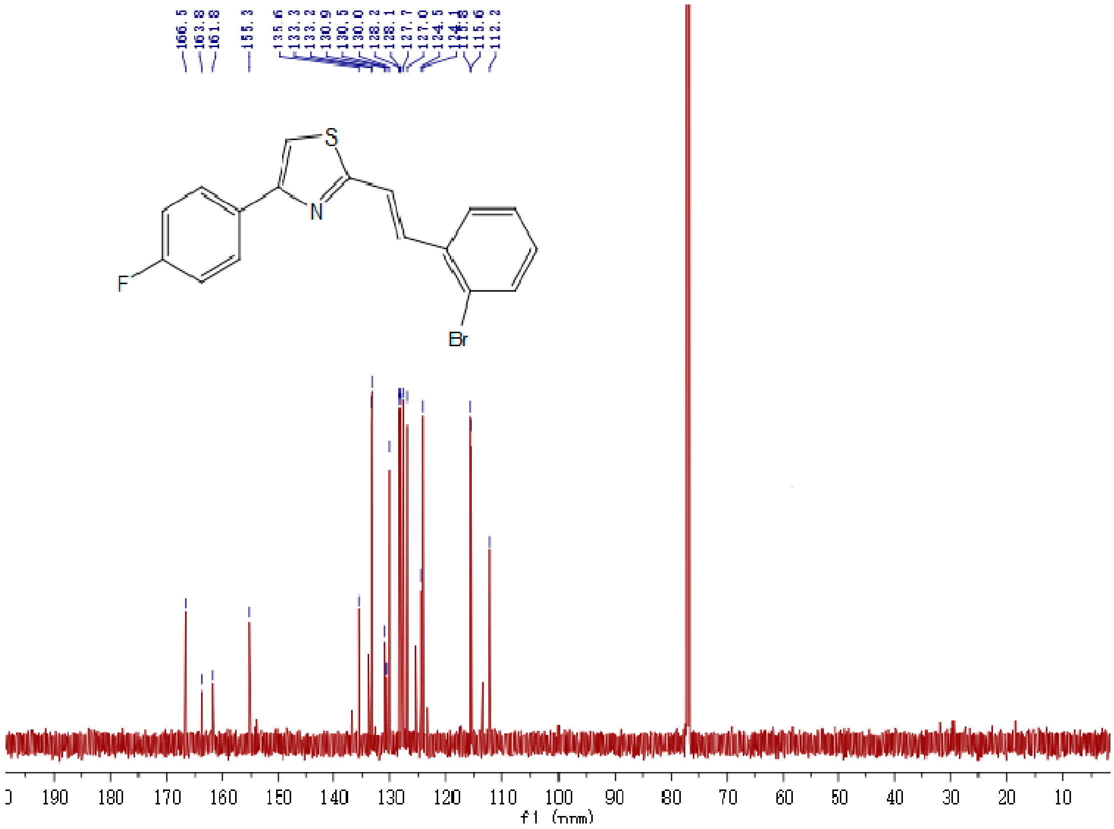

Figure S15. ^{1}H NMR and ^{13}C NMR of **18.**

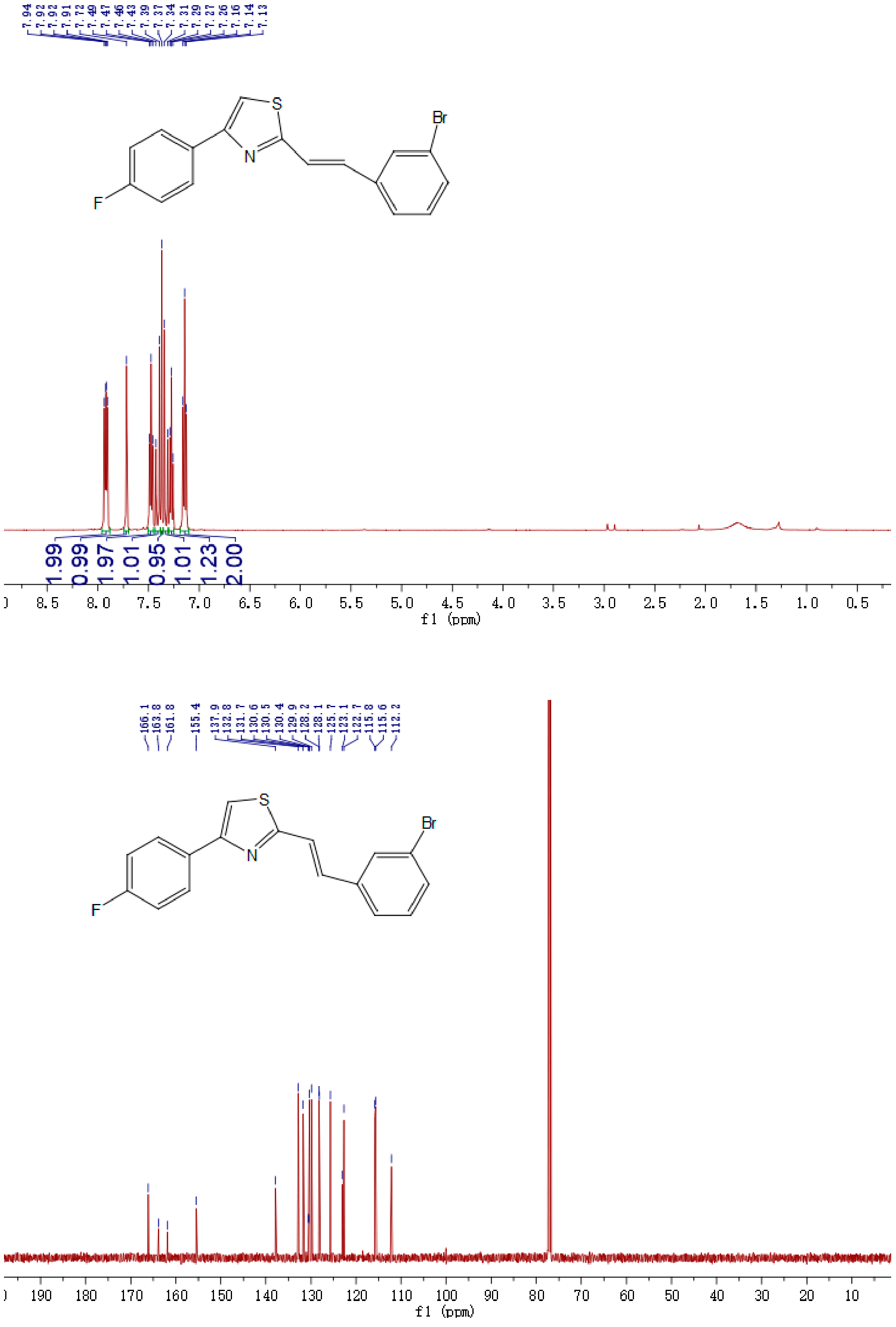

Figure S16. ^{1}H NMR and ^{13}C NMR of **19.**

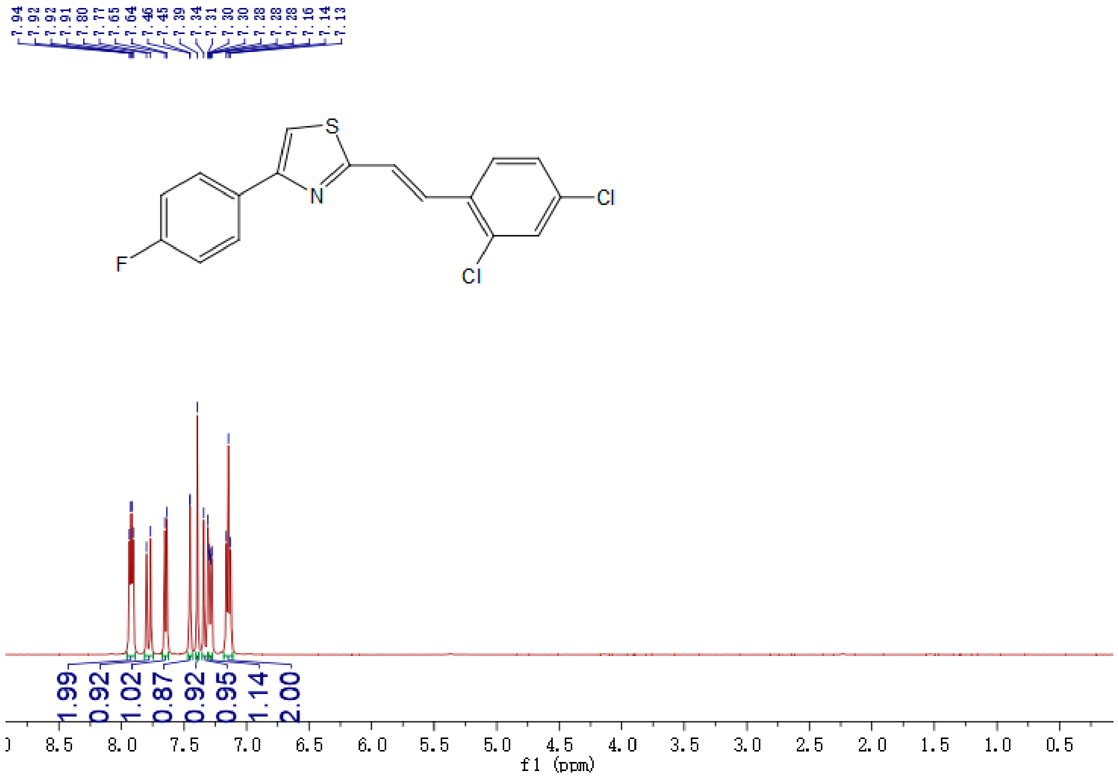

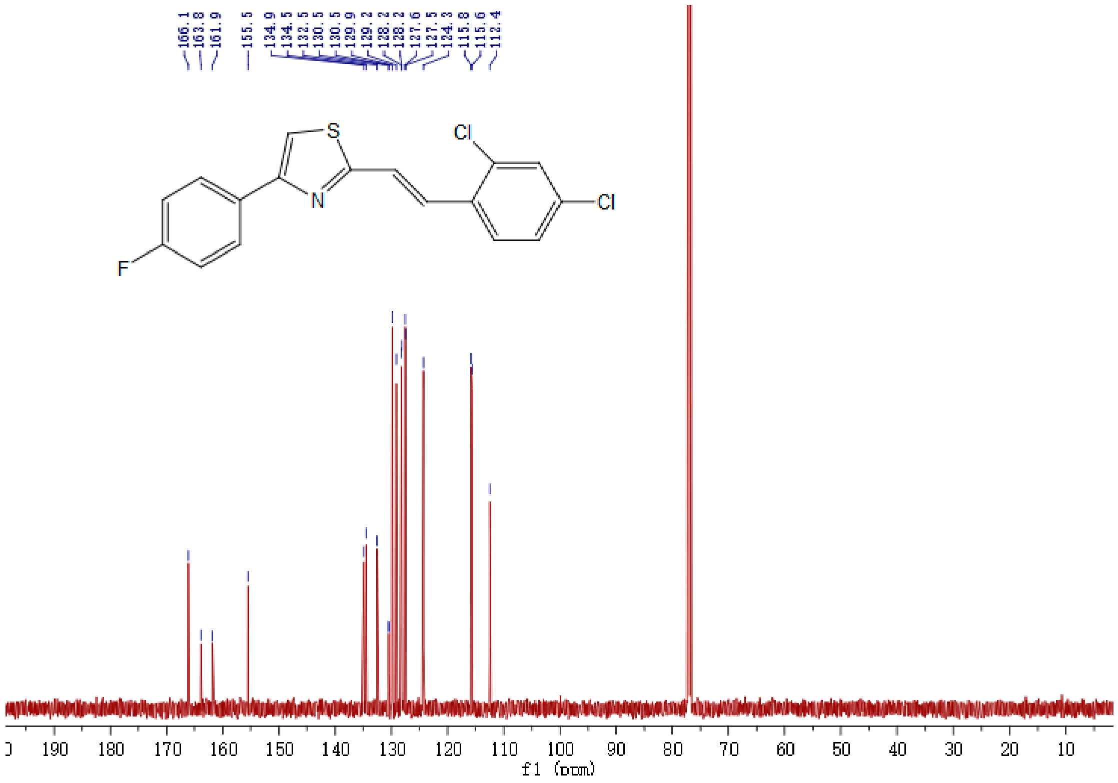

Figure S17. ^{1}H NMR and ^{13}C NMR of **20.**

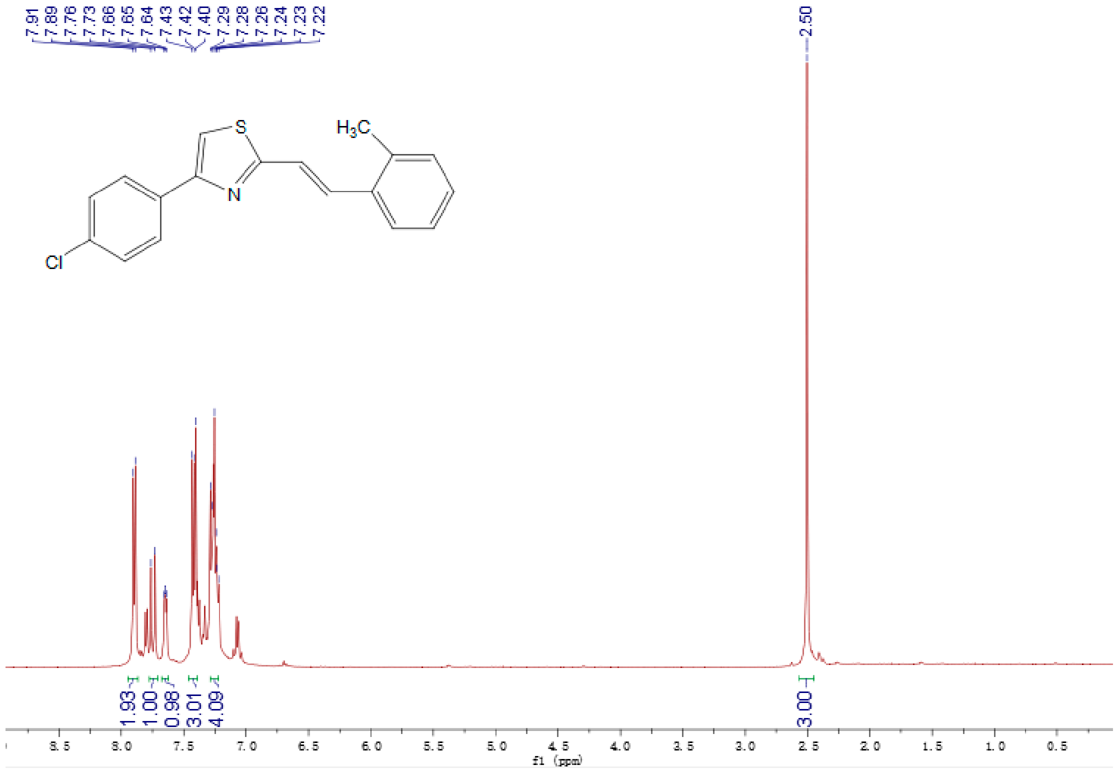

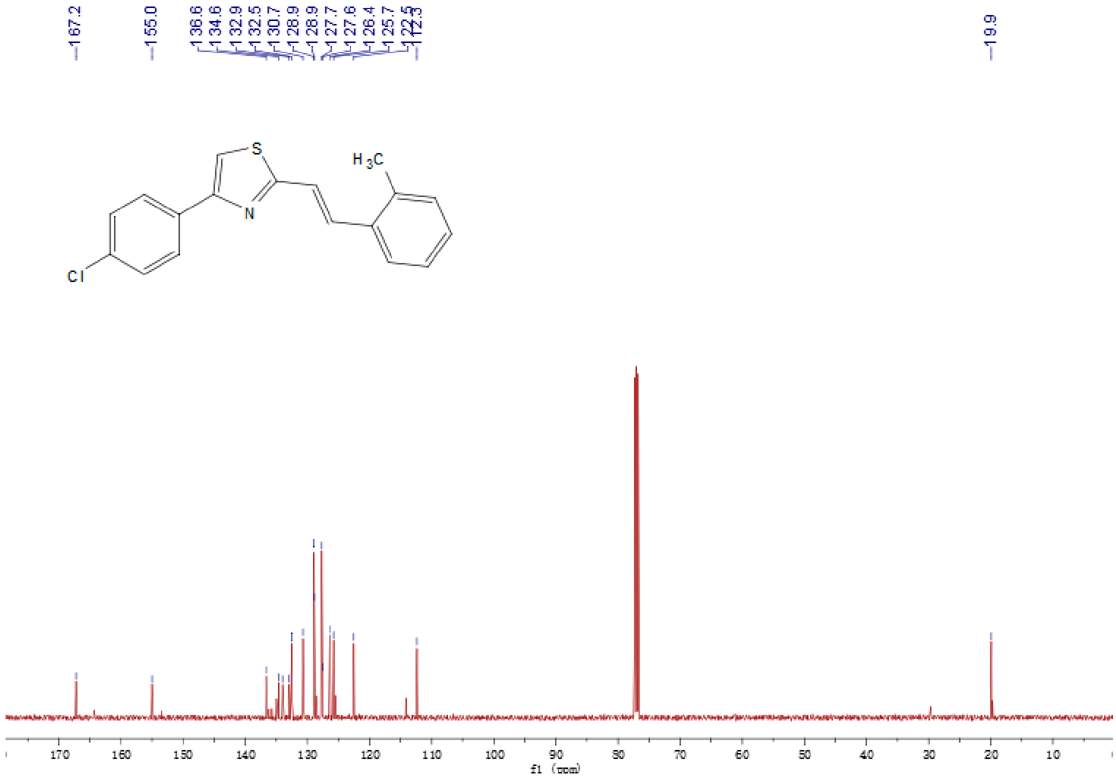

Figure S18. ^{1}H NMR and ^{13}C NMR of **21.**

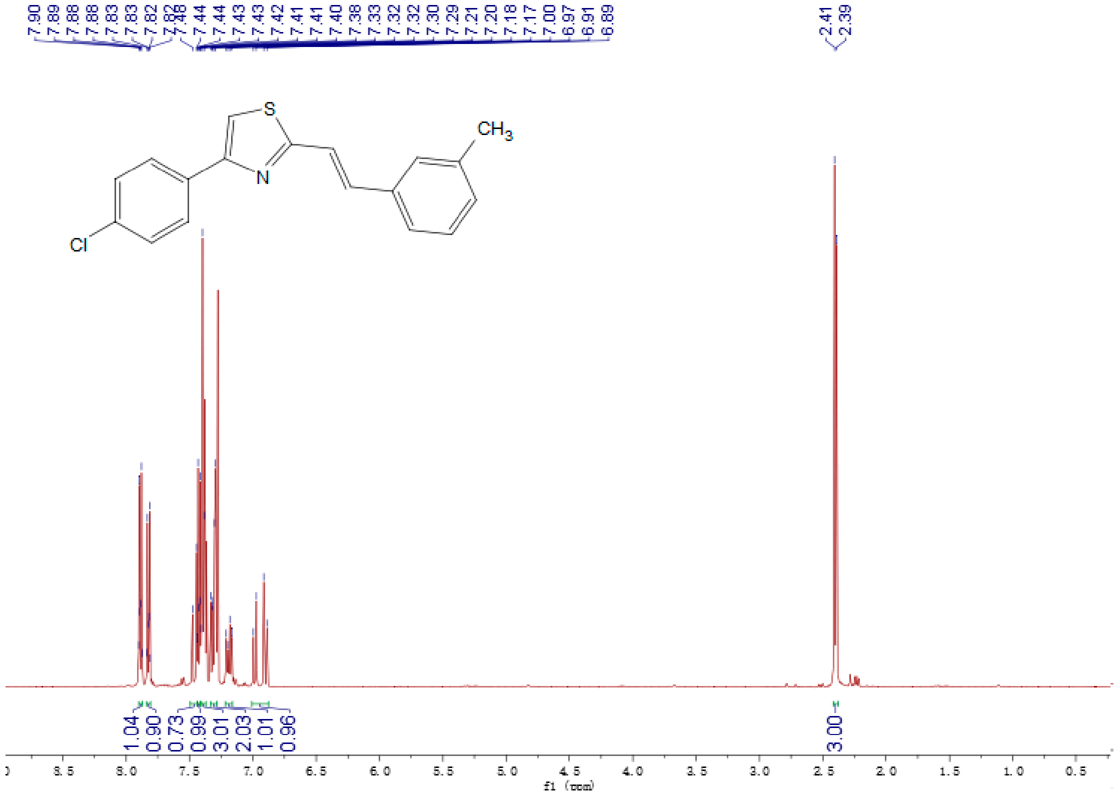

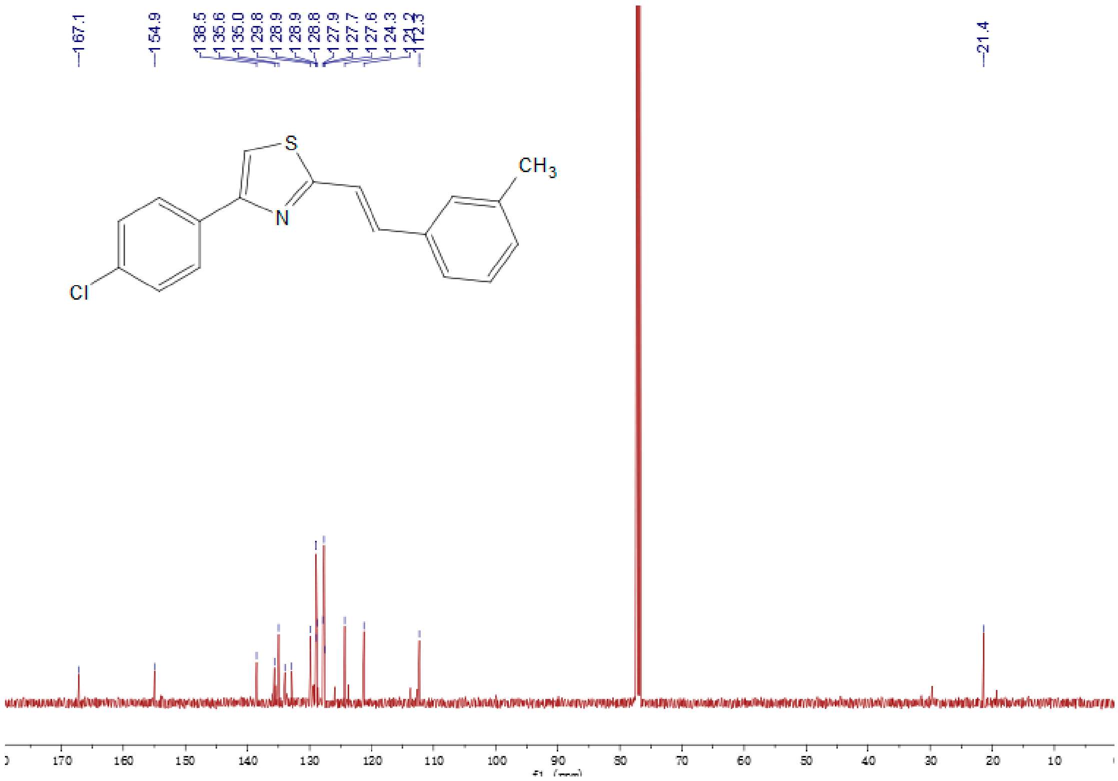

Figure S19. ^{1}H NMR and ^{13}C NMR of **22.**

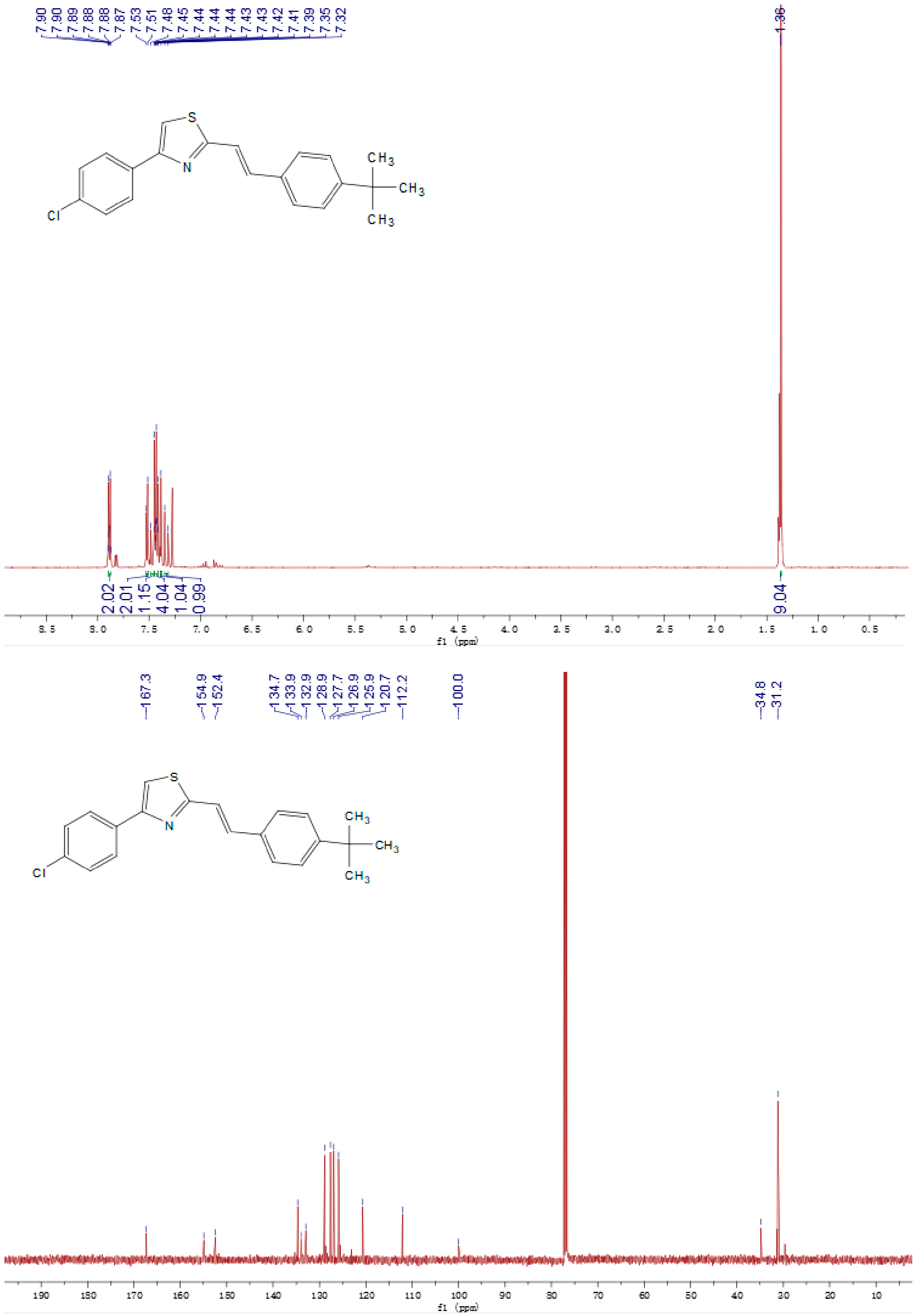

Figure S20. ^{1}H NMR and ^{13}C NMR of **23.**

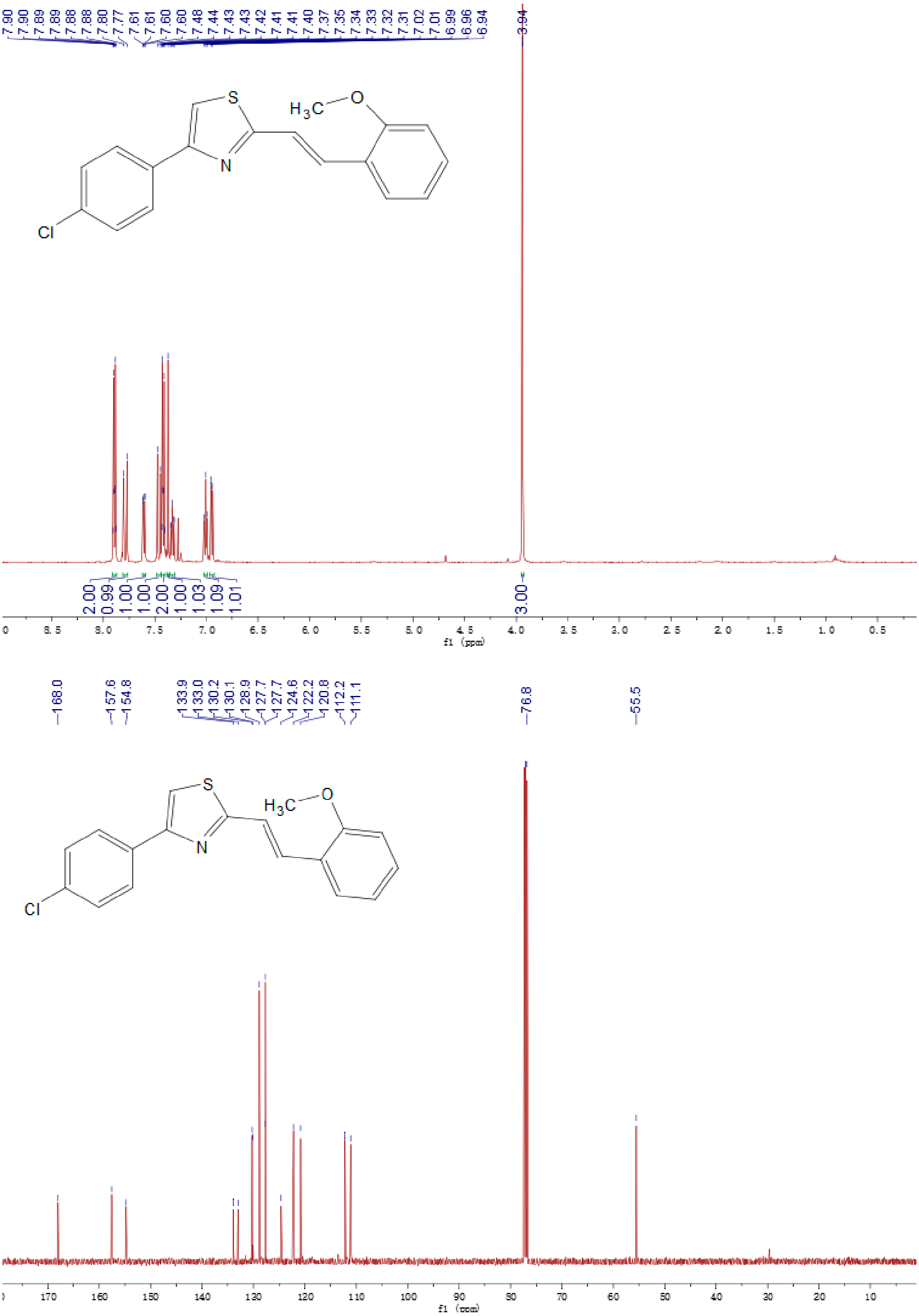

Figure S21. ^{1}H NMR and ^{13}C NMR of **24.**

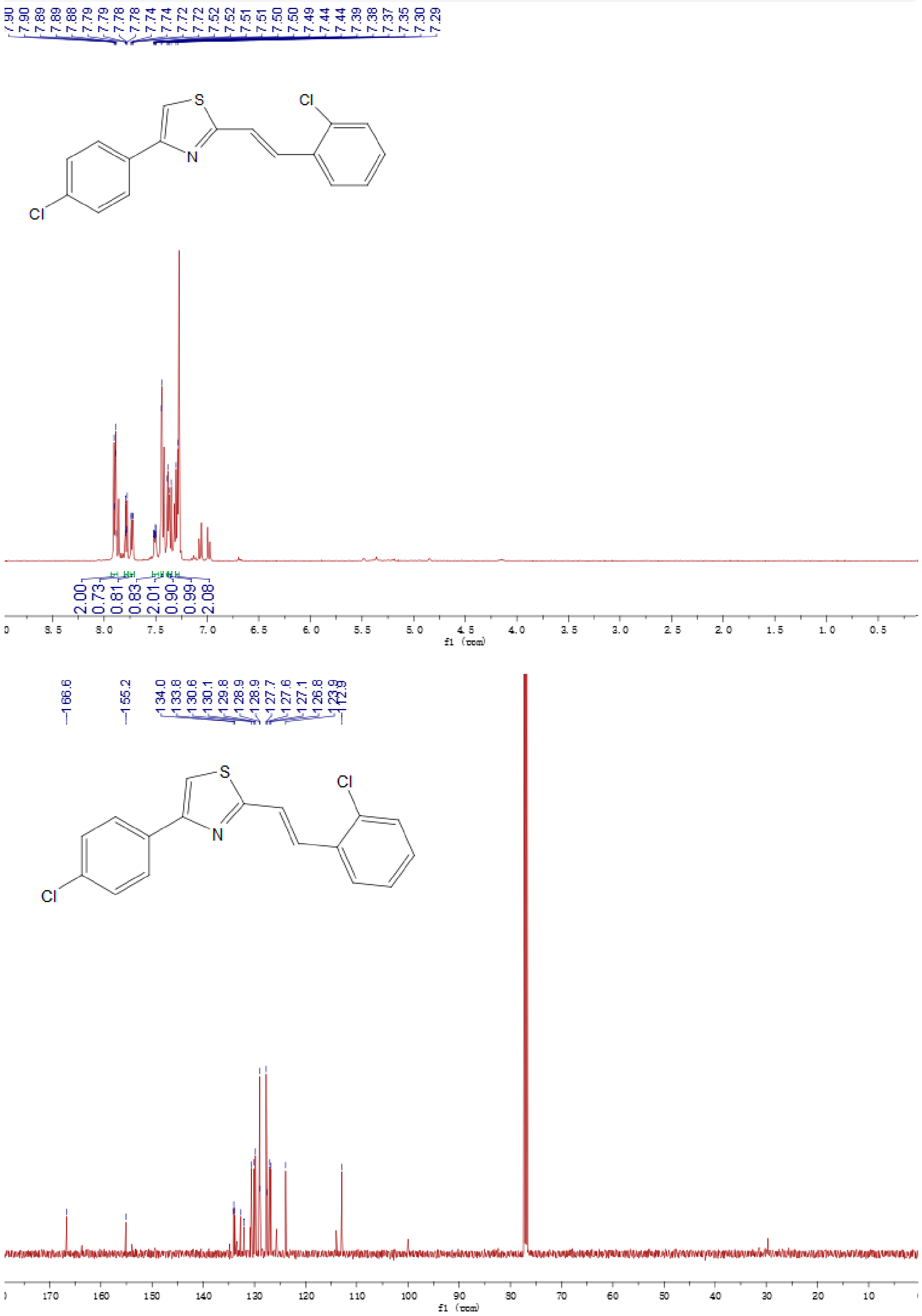

Figure S22. ^{1}H NMR and ^{13}C NMR of **25.**

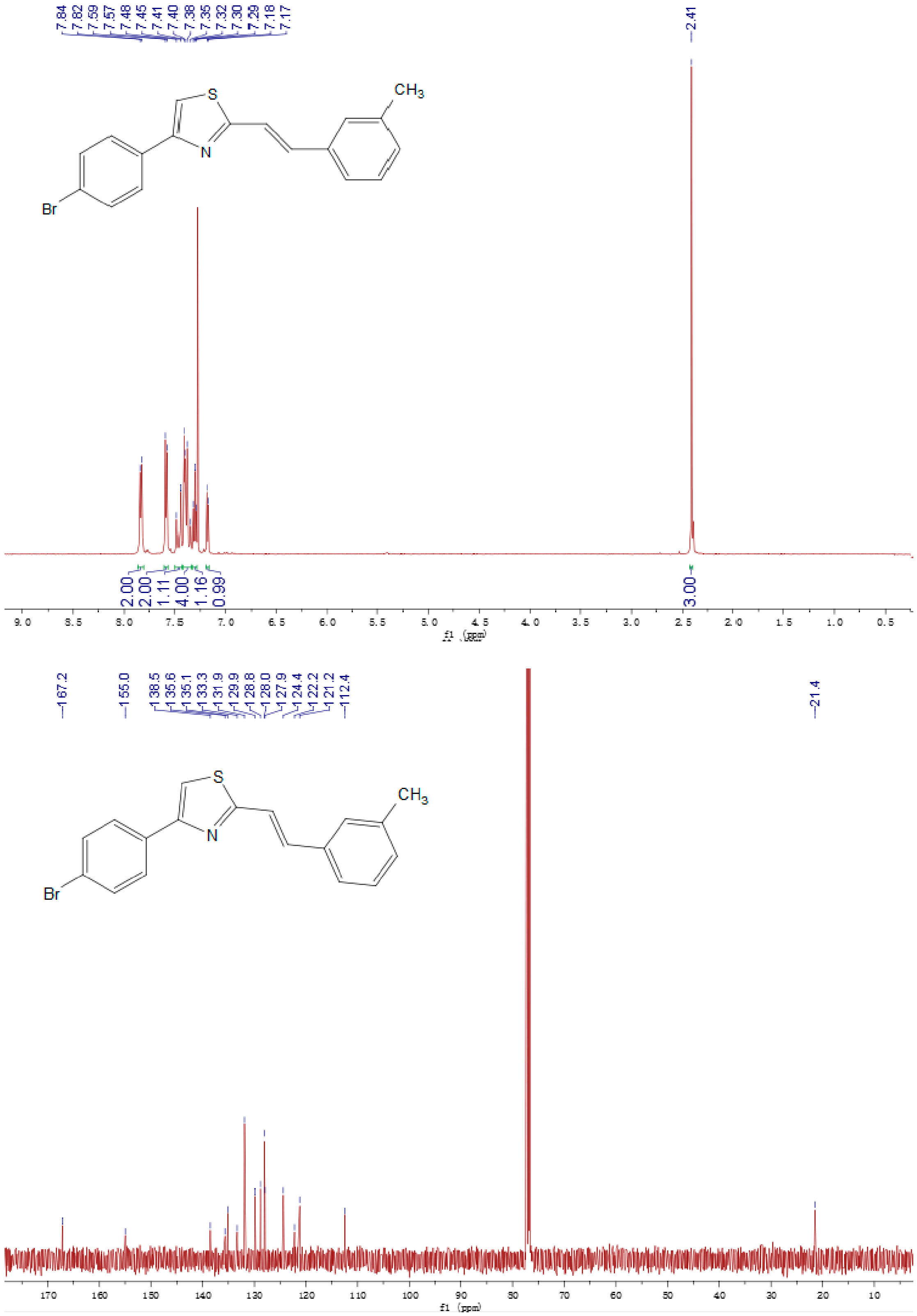

Figure S23. ^{1}H NMR and ^{13}C NMR of **26.**

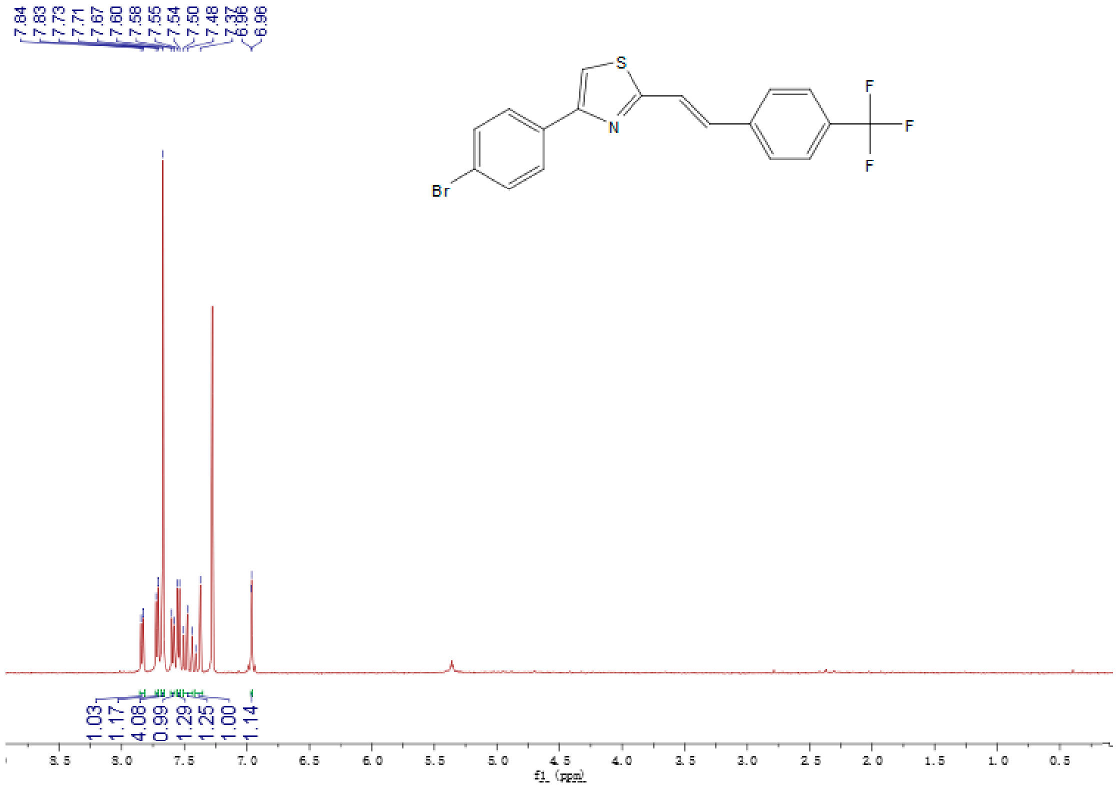

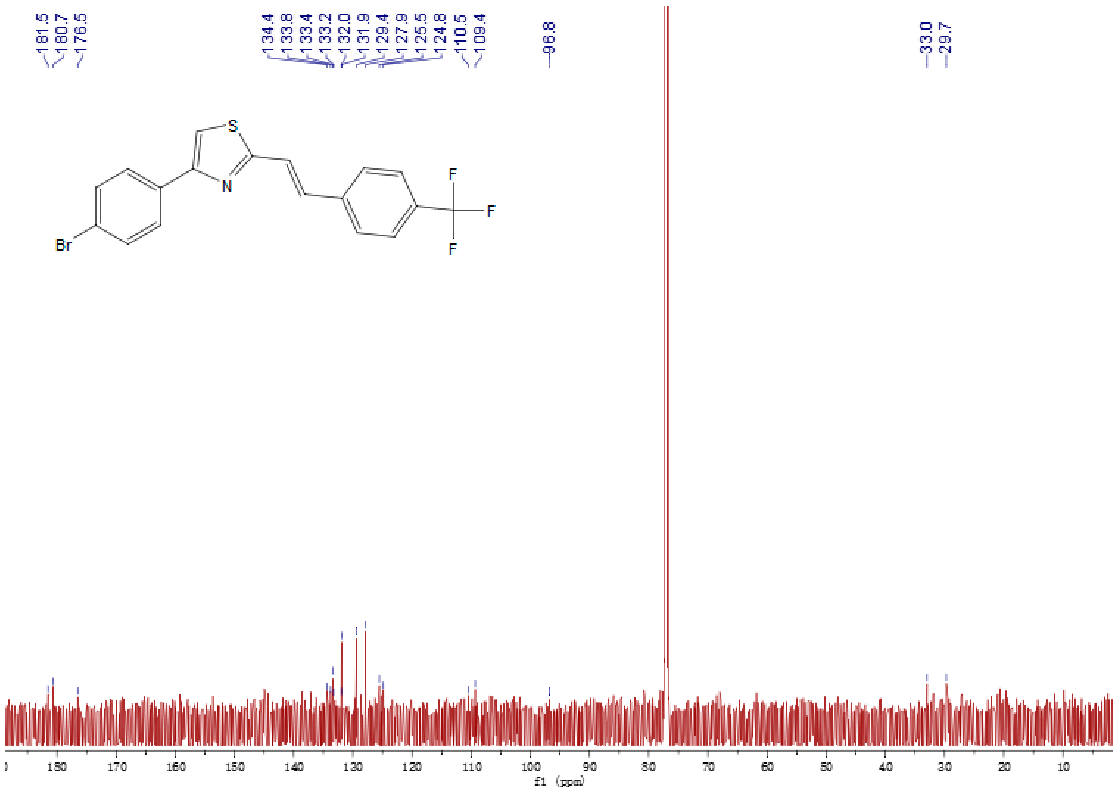

Figure S24. ^{1}H NMR and ^{13}C NMR of **27.**

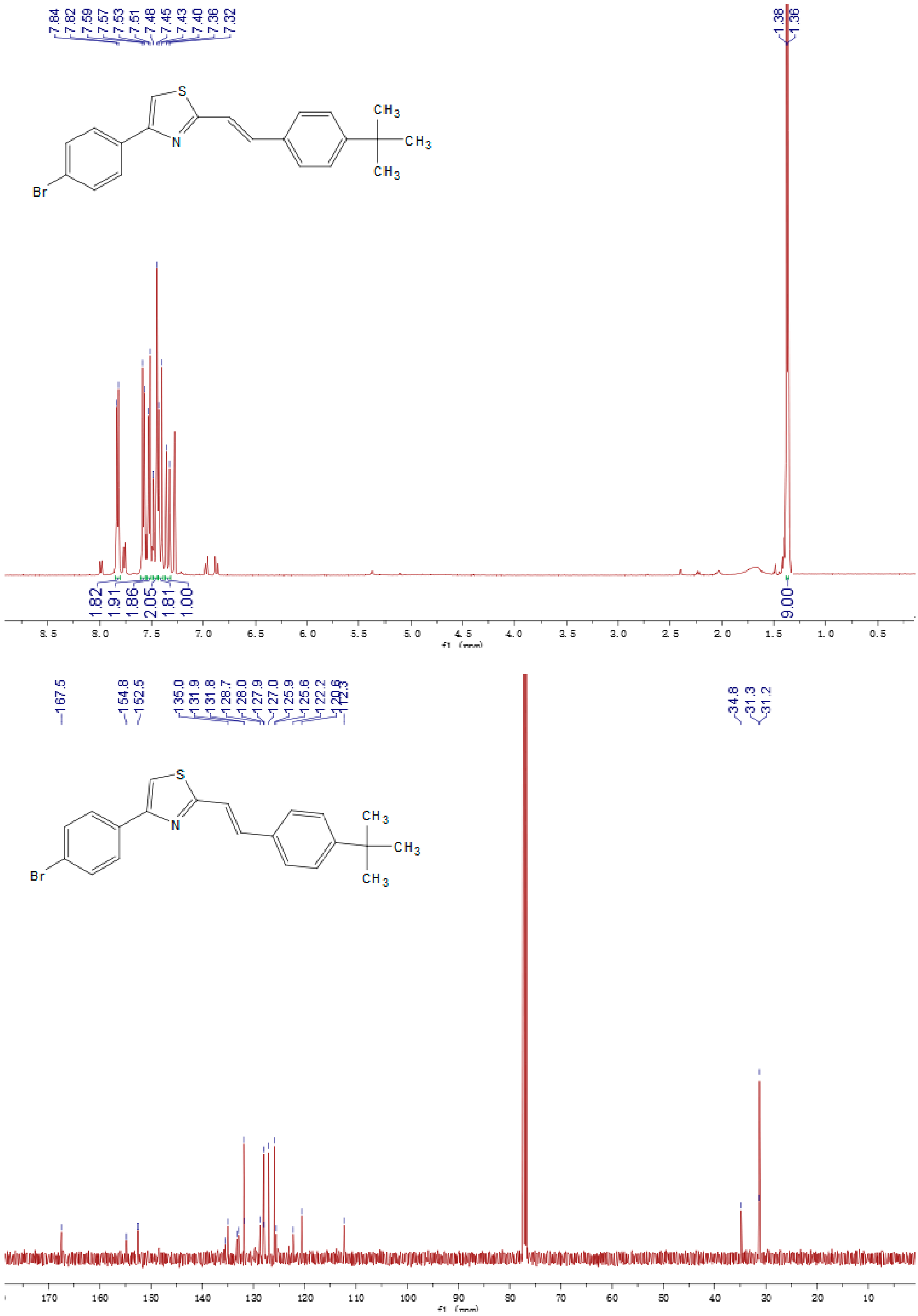

Figure S25 ^{1}H NMR and ^{13}C NMR of **28**

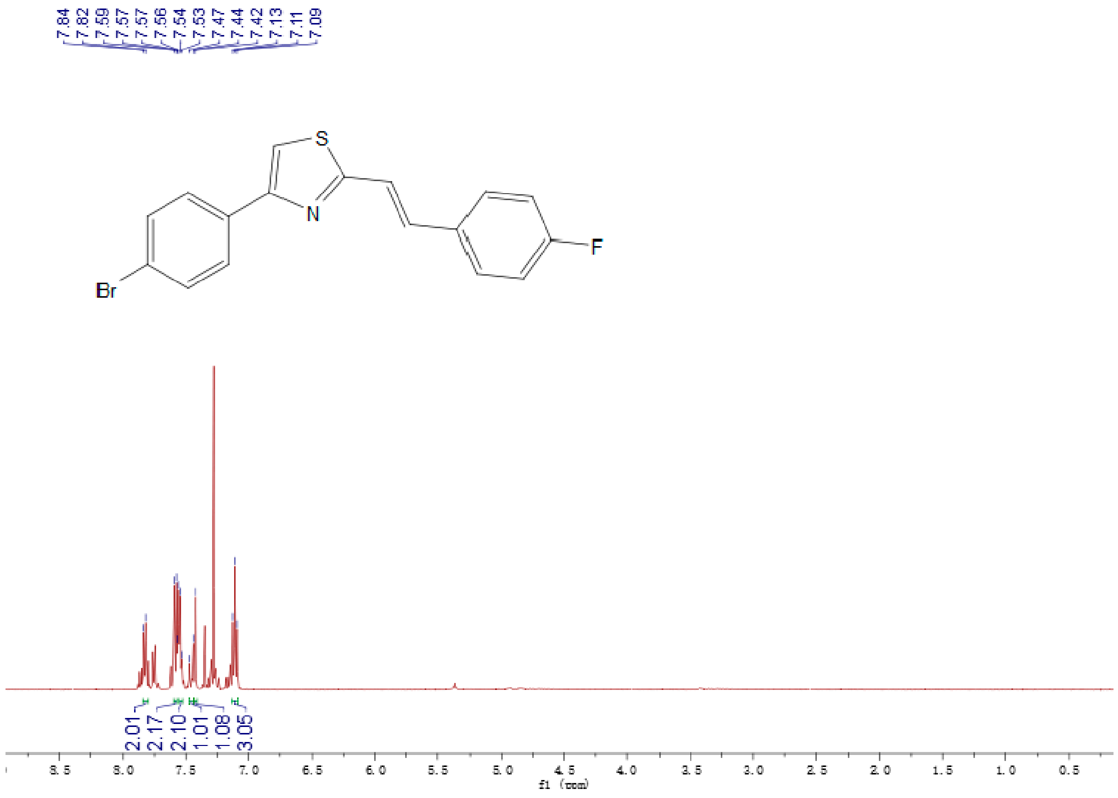

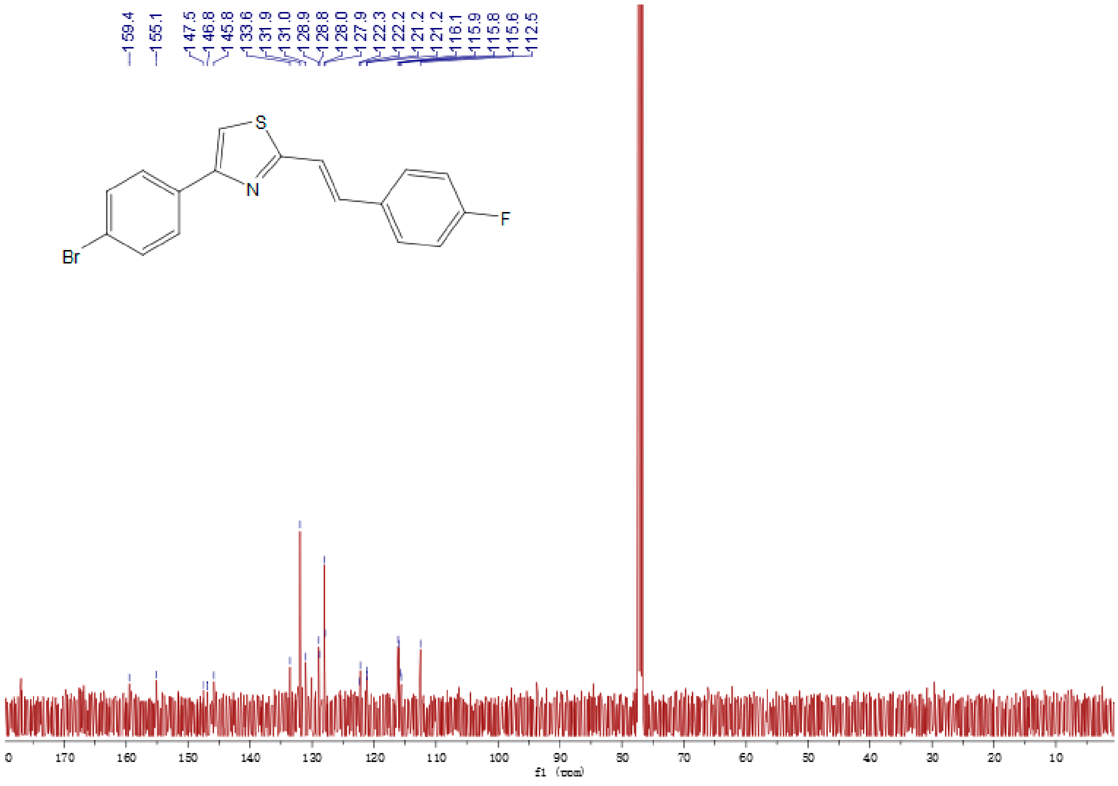

Figure S26. ^{1}H NMR and ^{13}C NMR of **29.**

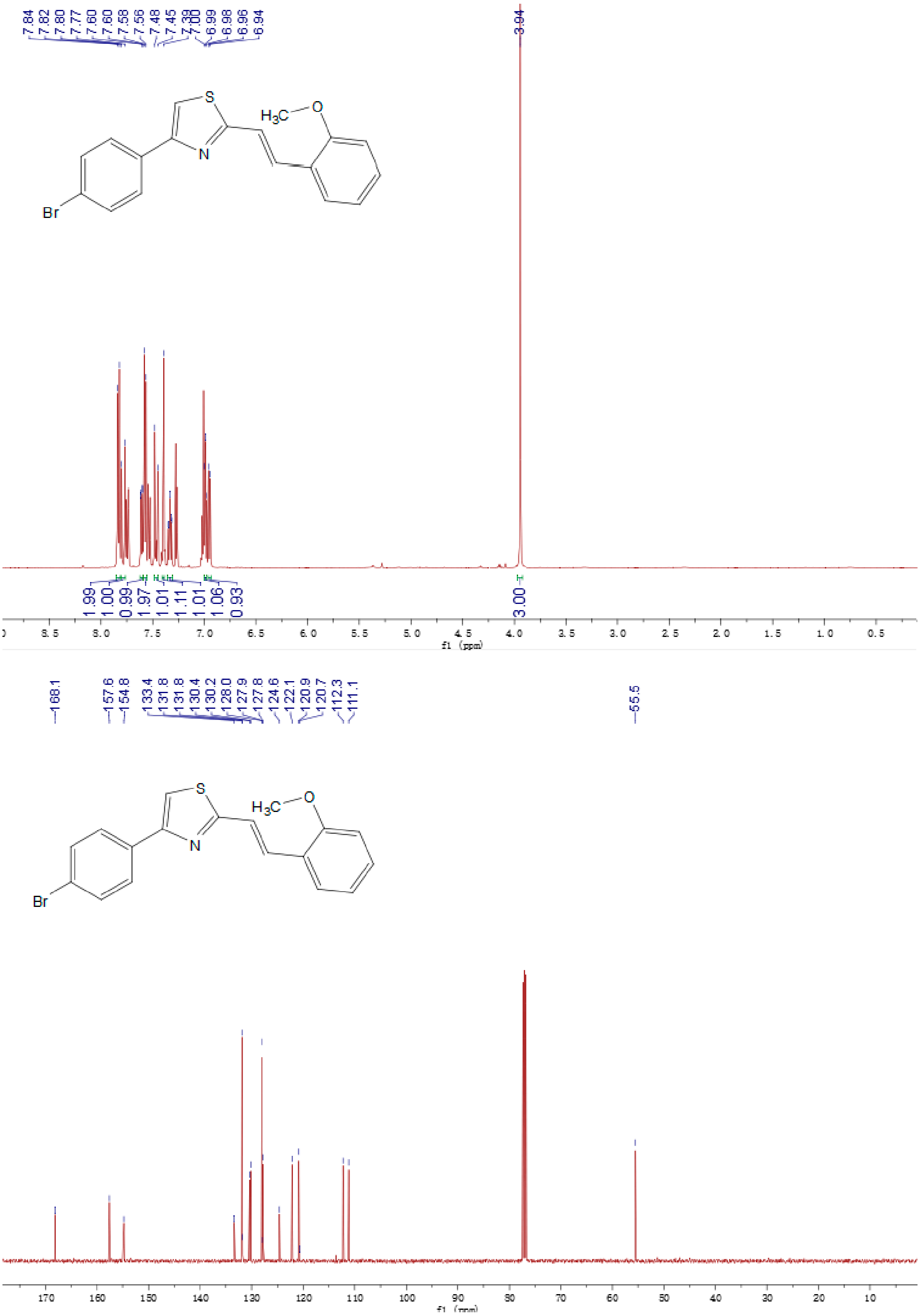

Figure S27. ^{1}H NMR and ^{13}C NMR of **30.**

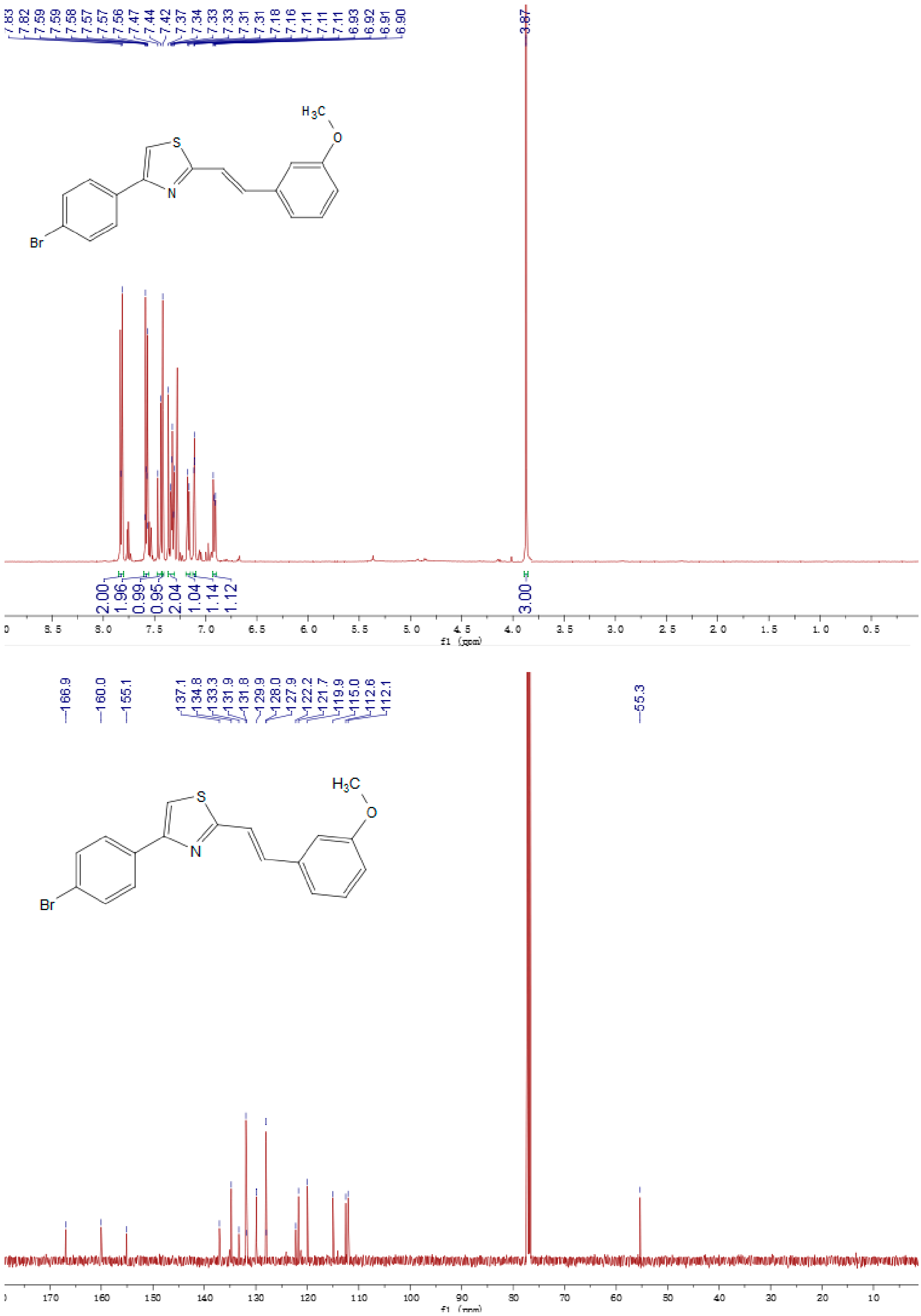

Figure S28. ^{1}H NMR and ^{13}C NMR of **31.**

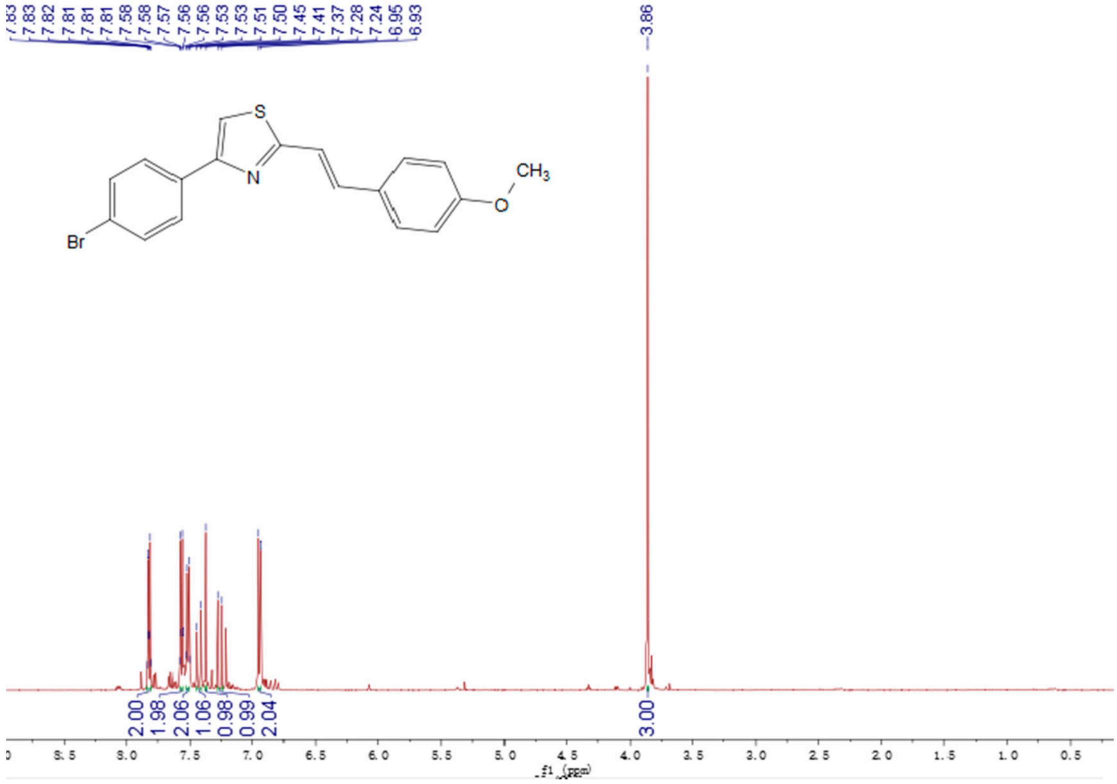

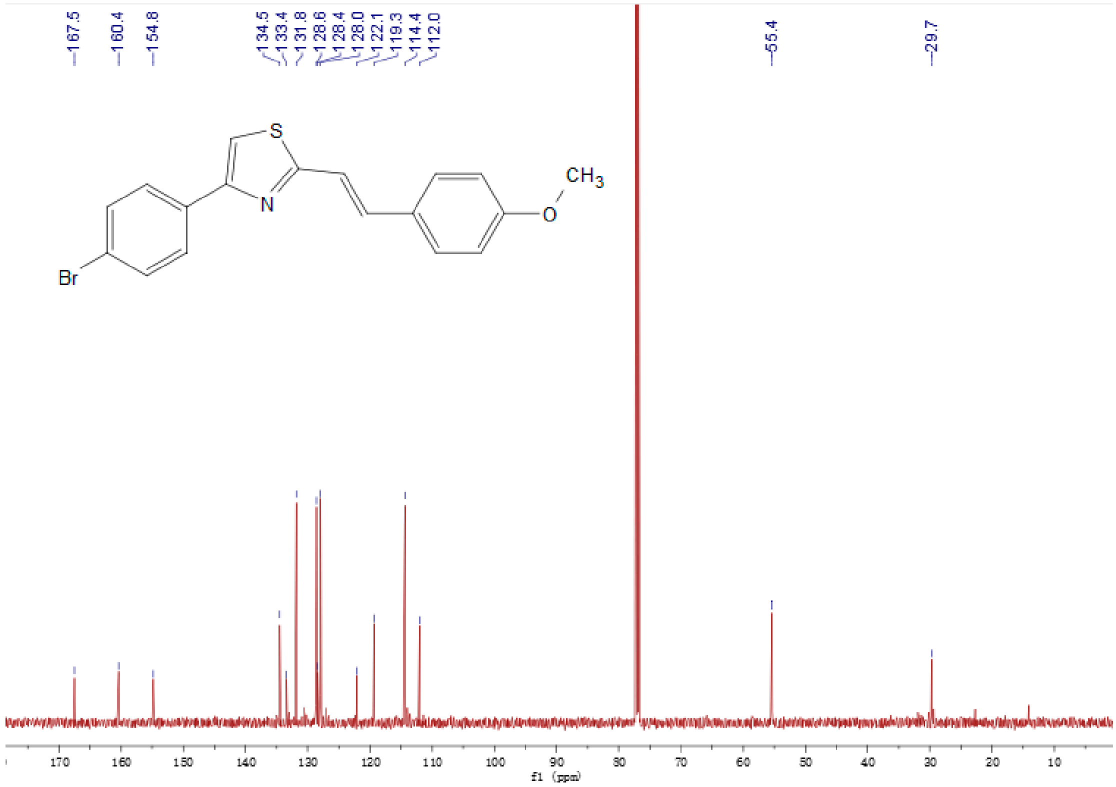

Figure S29. ^{1}H NMR and ^{13}C NMR of **32.**

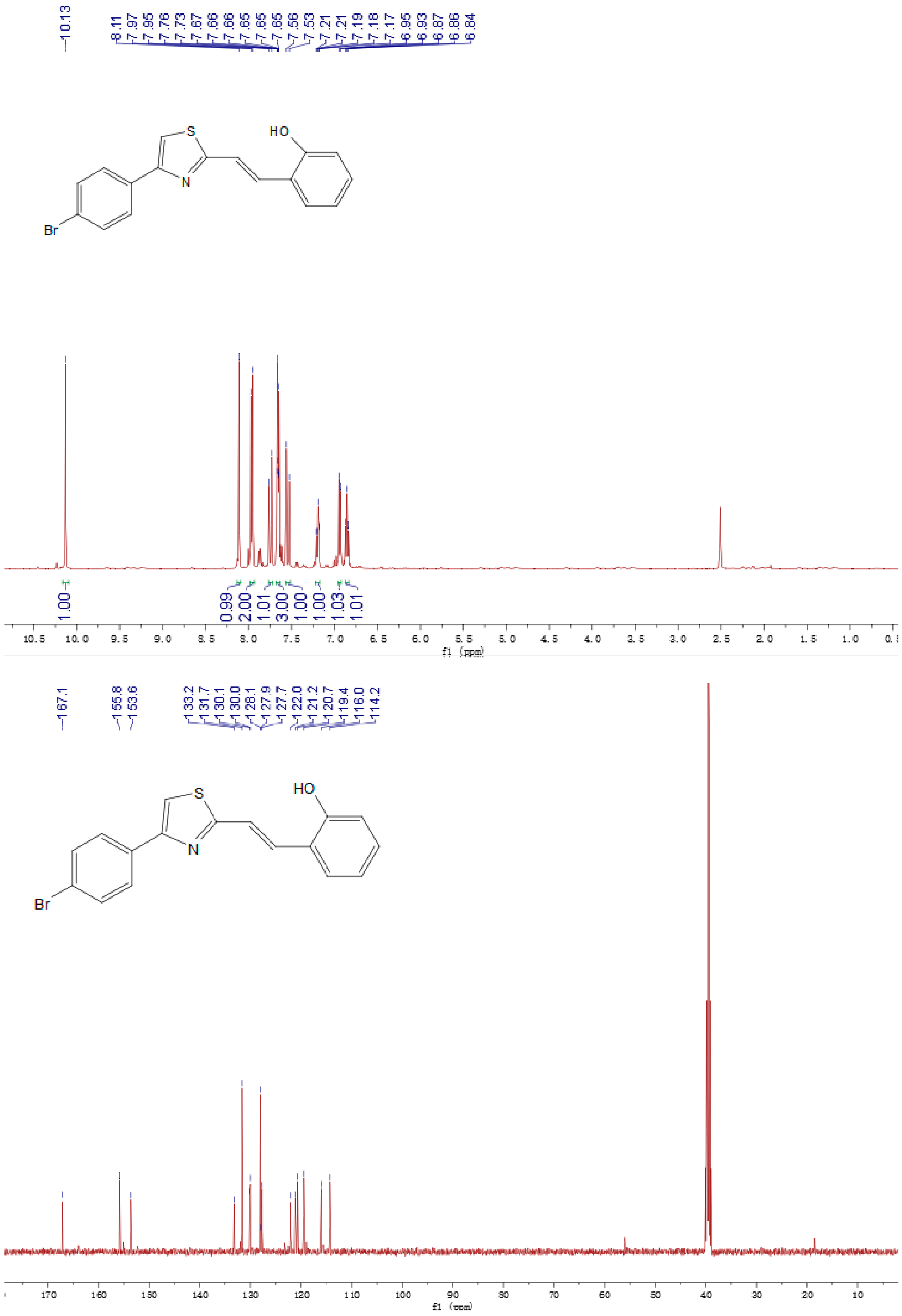

Figure S30. ^{1}H NMR and ^{13}C NMR of **33.**

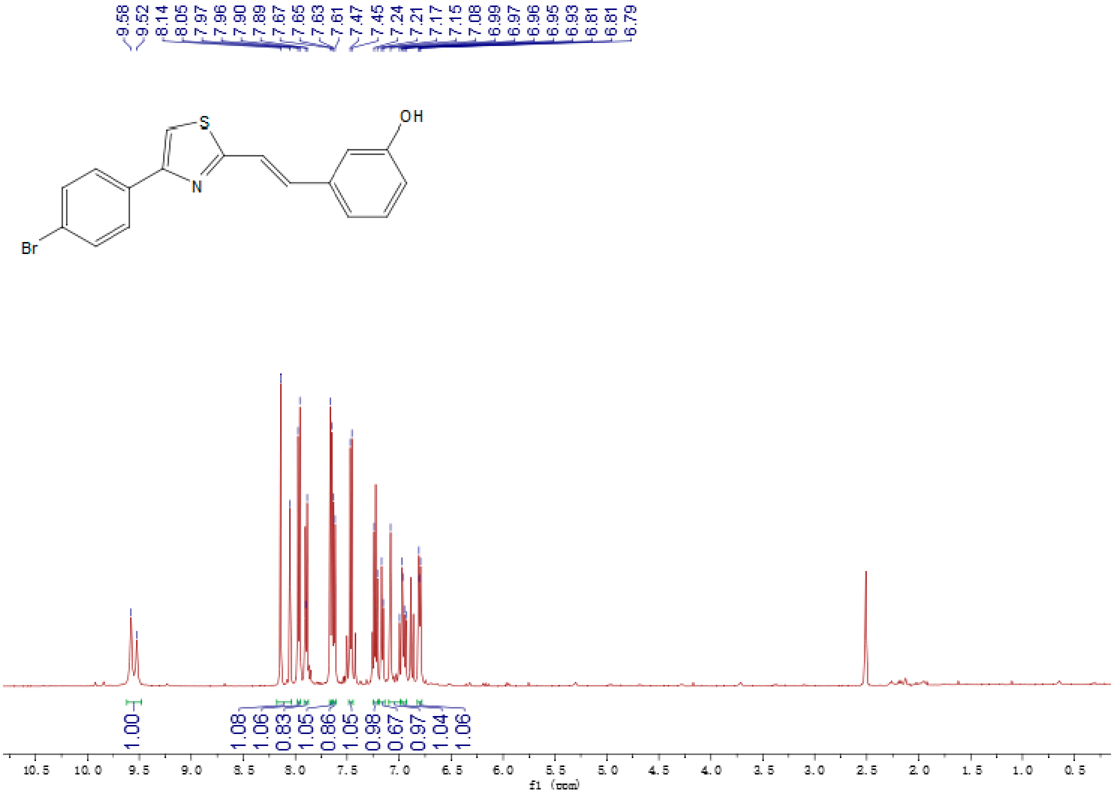

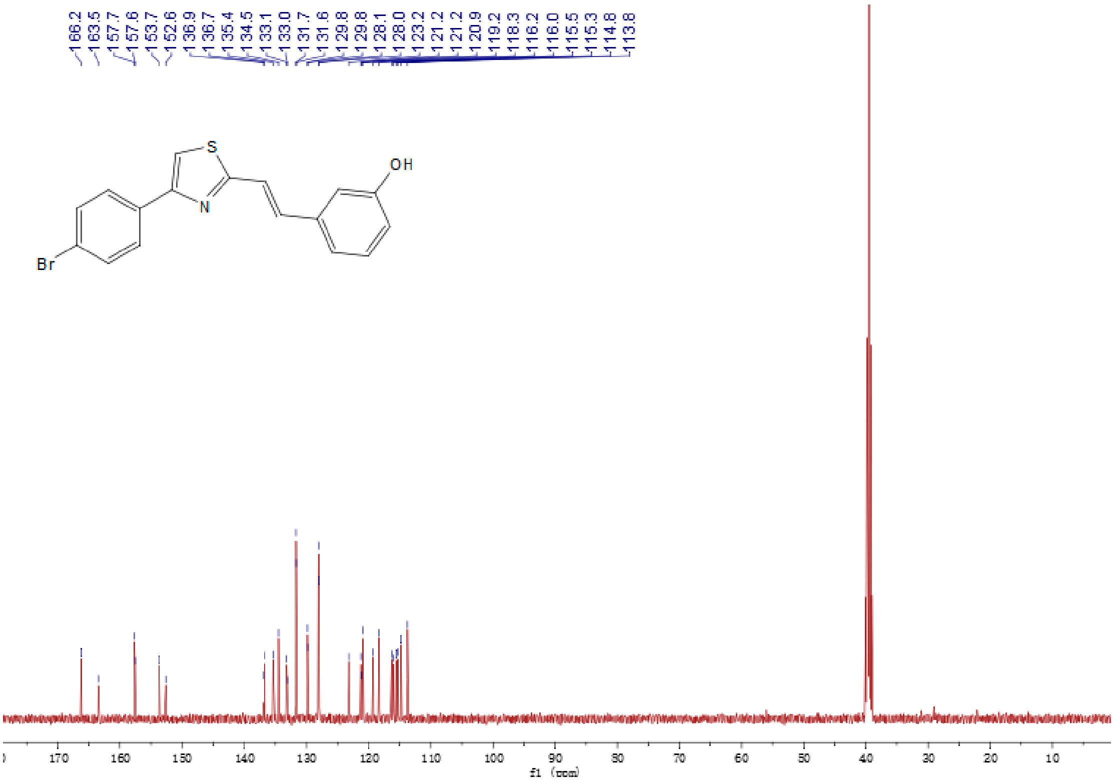

Figure S31. ^{1}H NMR and ^{13}C NMR of **34.**

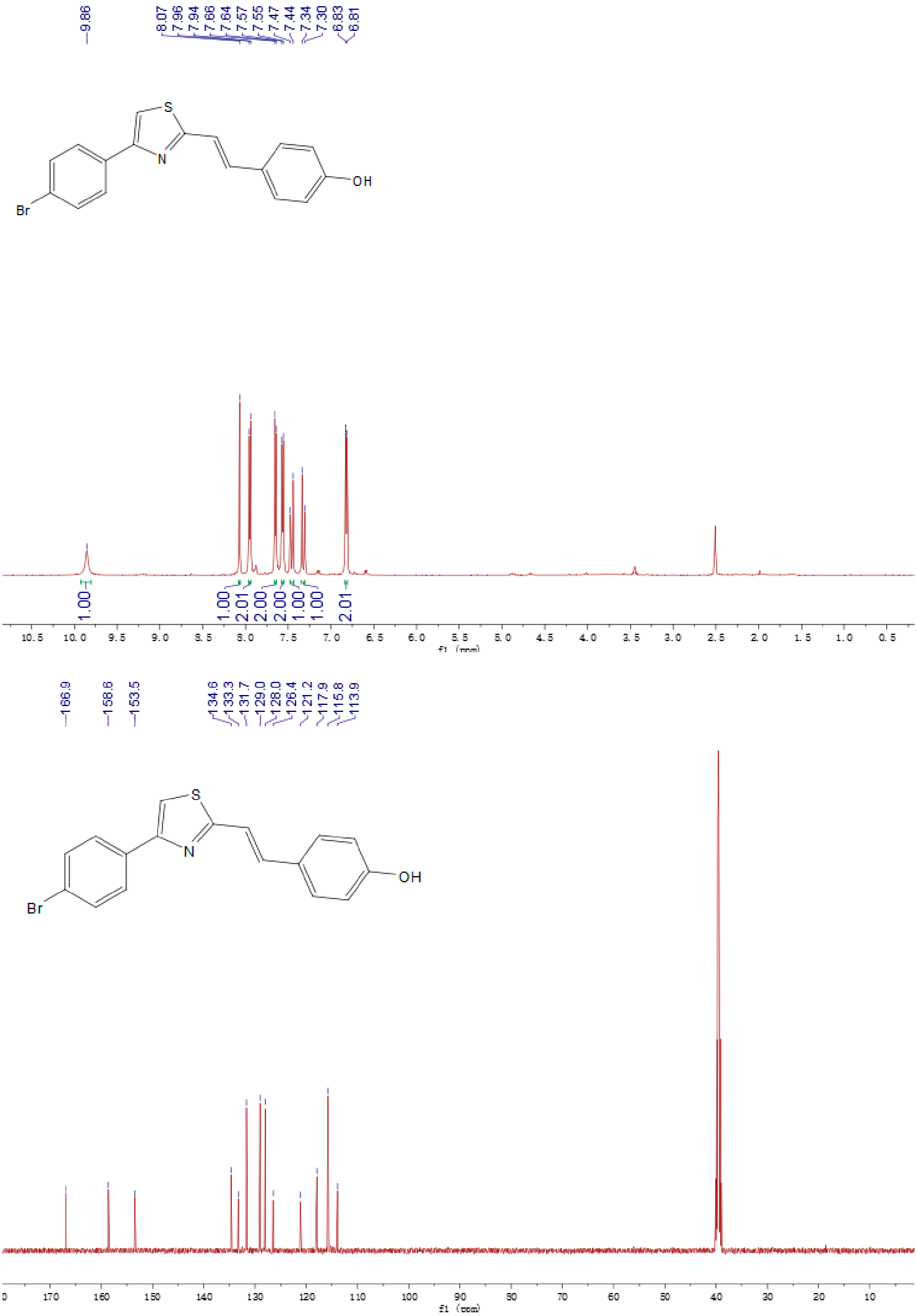

Figure S32. ^{1}H NMR and ^{13}C NMR of **35.**

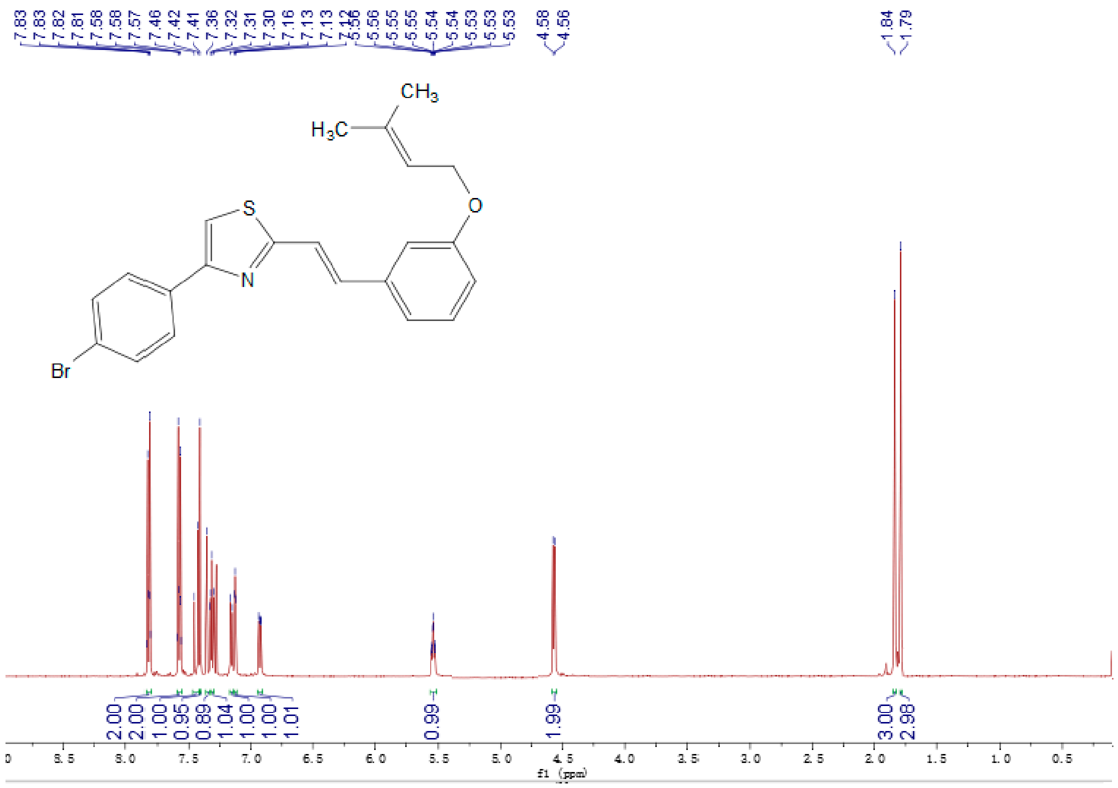

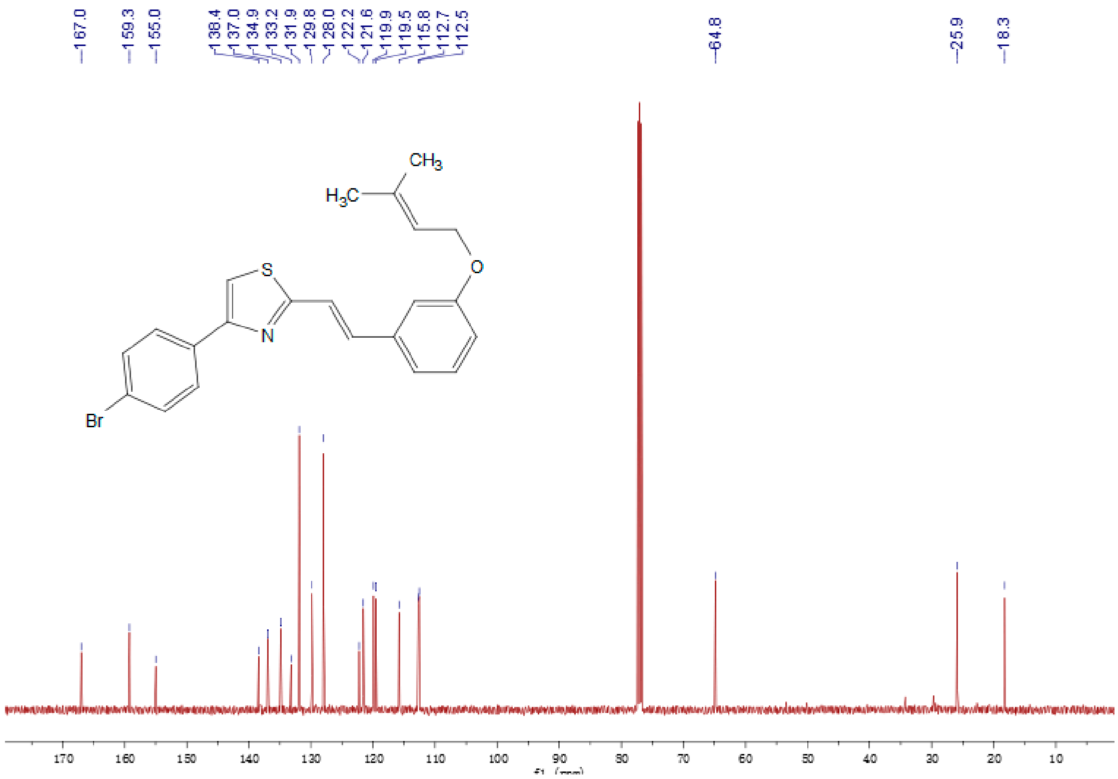

Figure S33. ^{1}H NMR and ^{13}C NMR of **36.**

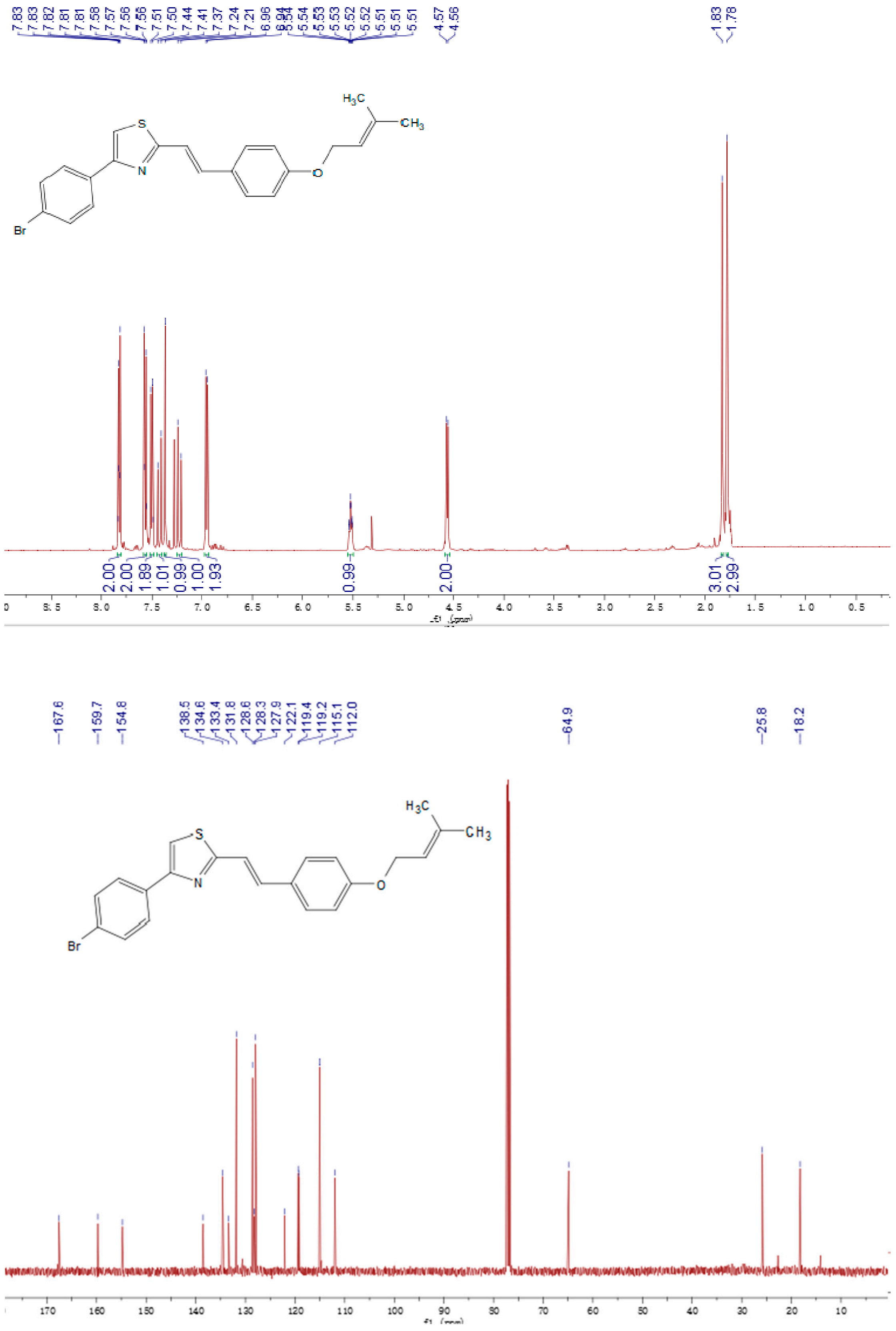

Figure S34. ^{1}H NMR and ^{13}C NMR of **37.**
